# Identification, Expression, and Interaction Analysis of Ovate Family Proteins in *Populus trichocarpa* Reveals a Role of PtOFP1 Regulating Drought Stress Response

**DOI:** 10.3389/fpls.2021.650109

**Published:** 2021-04-20

**Authors:** Hemeng Wang, Jin-Gui Chen, Ying Chang

**Affiliations:** ^1^Northeast Agricultural University, Harbin, China; ^2^Biosciences Division, Oak Ridge National Laboratory, Oak Ridge, TN, United States

**Keywords:** OFP, *Populus trichocarpa*, transcription factor, drought stress, *Arabidopsis*

## Abstract

Ovate family proteins (OFPs) are a family of plant growth regulators that play diverse roles in many aspects of physiological processes. OFPs have been characterized in various plant species including tomato, *Arabidopsis*, and rice. However, little is known about OFPs in woody species. Here, a total of 30 *PtOFP* genes were identified from the genome of *Populus trichocarpa* and were further grouped into four subfamilies based on their sequence similarities. Gene expression analysis indicated that some members of the *PtOFP* gene family displayed tissue/organ-specific patterns. Analysis of *cis*-acting elements in the promoter as well as gene expression by hormone treatment revealed putative involvement of *PtOFPs* in hormonal response. Furthermore, PtOFP1 (Potri.006G107700) was further experimentally demonstrated to act as a transcriptional repressor. Yeast two-hybrid assay showed physical interactions of PtOFP1 with other proteins, which suggests that they might function in various cellular processes by forming protein complexes. In addition, overexpression of *PtOFP1* in *Arabidopsis* conferred enhanced tolerance to PEG-induced drought stress at seedling stage, as well as a higher survival rate than the wild type at mature stage. These results provide a systematic analysis of the *Populus OFP* gene family and lay a foundation for functional characterization of this gene family.

## Introduction

Ovate family proteins (OFPs), which contain a 70-amino acid (aa) conserved domain at the C-terminal region named *OVATE* or DUF623 (the Domain of Unknown Function 623), are previously characterized as the novel plant-specific growth regulators (Hackbusch et al., 2005). The first *OVATE* gene was identified as a main quantitative trait locus (QTL) in controlling fruit appearance in tomato. A single mutation of *OVATE* leading to a premature stop codon caused the tomato fruit shape to shift from round- to pear-formed fruit or the elongated fruit shape (Liu et al., 2002). Subsequent studies revealed that OFPs are ubiquitously present in the plant kingdom. By using the amino acid sequences of OFPs in *Arabidopsis* and the OVATE protein in tomato to search genomes of 13 land plants including *Solanum lycopersicum*, *Solanum tuberosum*, *Mimulus guttatus* (asterid clade of core eudicots), *Arabidopsis thaliana*, *Vitis vinifera*, *Populus trichocarpa*, *Prunus persica*, *Carica papaya* (the rosid clade), *Aquilegia coerulea* (the basal eudicots), *Oryza sativa*, *Zea mays* (monocots), *Selaginella moellendorffii*, and *Physcomitrella patens*, Liu et al. (2014) found that OFPs are distributed in all the plants examined, including the seedless vascular plant *S. moellendorffii* (lycophytes seedless vascular plants) and the non-vascular plant *P. patens* (Liu et al., 2014). In addition, OFP family in four types of early land plants including *Marchantia polymorpha* (Mp), *P. patens* (Pp), *S. moellendorffii* (Sm), and *Sphagnum fallax* (Sf) were investigated specifically to provide insights into evolutionary history (Dangwal and Das, 2018).

Remarkably, most of the OFPs are proposed to function as transcription factors (TFs) and regulate plant development broadly. Wang et al. (2011) reported that OFPs in *Arabidopsis* function as transcriptional repressors and are involved in various aspects of plant growth and development (Wang et al., 2011). For instance, AtOFP1 was identified as a transcriptional repressor that regulates cell elongation by directly controlling the expression of *AtGA20ox1* (Wang et al., 2007). KNAT7–OFP4 protein interactions enhanced KNAT7-mediated transcriptional repression to its downstream target genes that participate in the regulation of secondary cell wall formation (Li et al., 2011). Subcellular localization analysis revealed that OFP proteins in rice (OsOFPs) are predominantly localized in the nucleus, which implied that OsOFPs may act as transcriptional regulators during seed development (Yu et al., 2015). In general, TFs serve as regulators of cellular processes by interacting with other proteins. Hackbusch et al. (2005) reported that nine members of AtOFPs tend to interact with TALE (3-aa loop extension) homeodomain proteins and regulate plant meristem fundamental function and leaf development by forming protein complexes (Hackbusch et al., 2005). OsOFP2 overexpression led to reduced plant height, altered leaf, seed morphology, and stem vascular development by modulating KNOX-BELL function (Schmitz et al., 2015). The ATH1–OFP1 protein complex was proposed to be involved in the regulation of flowering transition, stem growth, and the formation of flower basal boundary (Zhang L. et al., 2018). Besides TALE protein, OFPs interact with other types of proteins. In a subsequent study, it was demonstrated that AtOFP1 is able to interact with the AtKu70 protein, a protein that is involved in the non-homologous end-joining (NHEJ) pathway (Wang et al., 2010).

Accumulating evidence suggested that OFPs are involved in a range of biological processes, and their functions are often found to be associated with plant hormones and environmental stresses (Liu et al., 2015; Tang et al., 2018; Wu et al., 2018). In rice, OsOFP1, OsOFP8, and OsOFP19 play pivotal roles in modulating brassinosteroid (BR) signaling pathway by determining cell division pattern (Yang et al., 2016, 2018; Xiao et al., 2017). In addition, available experimental evidence suggested that OFPs play multiple roles in responses to diverse abiotic stresses. Within Rosaceae species, five *PbrOFP* genes were significantly upregulated following PEG treatment in Chinese pear (*Pyrus bretschneideri*), while the expression levels of *MdOFP04* and *MdOFP20* were higher under NaCl treatment in apple (*Malus domestica*) compared with the control group (Ding et al., 2020).

Despite the fact that much is known about OFPs in herbaceous plants, only a few studies were related to *OFP* genes in woody species (Liu et al., 2014). At present, there is no systematic analysis of the *P. trichocarpa OFP* gene family. As an important tree species of shelterbelt and timber forest and as a promising feedstock for biofuel conversion and production, poplar trees have enormous economic and ecological benefits, as well as unique biological properties of scientific interest. Thanks to the completion of the *P. trichocarpa* genome sequence in 2006, *Populus* has also become a model tree for other tree species (Tuskan et al., 2006). In this study, we report the comprehensive genome-wide identification and phylogenetic analysis and gene expression profiles of all 30 members of the *OFP* gene family in *Populus*.

## Results

### Identification of OFP Family in *P. trichocarpa*

A total of 30 genes were identified in the *P. trichocarpa* genome, which we designated as PtOFP1-PtOFP30 (Table 1). The amino acid sequence alignment indicated that the OVATE domain is mostly present at the C-terminus of these proteins (Supplementary Figure 1). The characteristic parameters of these predicted PtOFP proteins, including the length of the CDS (Coding Sequence), the protein length, molecular weight (MW), theoretical pI, and grand average of hydropathicity (GRAVY) score, are listed in Table 1. PtOFP amino acid sequences varied in length from 79 to 474 aa, and with an average of 269.6 aa. Among these 30 PtOFP proteins, PtOFP23 (Potri.014G181300) was the smallest protein with 9.61 kDa, whereas the largest one was PtOFP4 (Potri.016G134200) with 53.73 kDa. In addition, their isoelectric points ranged from 4.49 (Potri.010G241500) to 9.99 (Potri.005G125200). GRAVY values are defined as the sum of the hydropathy values of all amino acids divided by the protein length. All PtOFPs are hydrophilic as indicated by the negative GRAVY values. The subcellular localization of a protein is closely related to its functional involvement. In this study, the prediction of PtOFPs subcellular localization indicated that most members are localized in the nucleus, whereas only a few are predicted to localize in the mitochondrial or chloroplast.

**TABLE 1:** The *OFP* gene family in *Populus trichocarpa* along with their molecular characteristics.

Gene ID	Gene accession number	Description		Physicochemical parameters			Subcellular localization
		CDS(bp)	Length (aa)	MW (kDa)	pI	GRAVY	
***PtOFP1***	Potri.006G107700.1	1362	453	51.498	9.46	–0.845	Nucleus
***PtOFP2***	Potri.006G158800.1	822	273	31.316	9.68	–0.726	Nucleus
***PtOFP3***	Potri.006G205500.1	825	274	30.040	4.52	–0.39	Extracellular
***PtOFP4***	Potri.016G134200.1	1425	474	53.737	9.19	–0.788	Nucleus
**PtOFP5**	Potri.016G072800.1	771	256	28.956	8.69	–0.591	Nucleus
***PtOFP6***	Potri.016G072900.1	801	266	28.797	4.68	–0.336	Extracellular/Chloroplast
***PtOFP7***	Potri.013G155200.1	1299	432	49.859	9.38	–0.754	Chloroplast
***PtOFP8***	Potri.019G128500.1	1161	386	44.032	9.26	–0.809	Nucleus
***PtOFP9***	Potri.004G062100.1	1296	431	49.690	9.3	–0.982	Nucleus
***PtOFP10***	Potri.004G003700.1	471	156	18.449	8.43	–0.715	Nucleus
***PtOFP11***	Potri.004G200500.1	543	180	19.921	5.45	–0.737	Nucleus
***PtOFP12***	Potri.018G080800.1	879	292	33.683	9.74	–0.636	Nucleus
***PtOFP13***	Potri.002G262500.1	1035	344	38.861	9.57	–0.565	Nucleus
***PtOFP14***	Potri.007G028000.1	1086	361	41.518	9.83	–0.809	Nucleus
***PtOFP15***	Potri.005G125200.1	1077	358	41.043	9.99	–0.775	Nucleus
***PtOFP16***	Potri.005G211300.1	741	246	26.805	4.92	–0.423	Nucleus
***PtOFP17***	Potri.009G161700.1	687	228	25.774	6.81	–0.481	Nucleus
***PtOFP18***	Potri.009G161600.1	666	221	25.331	5.5	–0.465	Extracellular
***PtOFP19***	Potri.008G153500.1	636	211	23.613	5.71	–0.415	Nucleus/Chloroplast
***PtOFP20***	Potri.008G017500.1	645	214	23.854	4.69	–0.399	Cytoplasmic/Nucleus
***PtOFP21***	Potri.010G087200.1	1084	218	24.137	5.95	–0.296	Extracellular
***PtOFP22***	Potri.010G241500.1	633	210	23.656	4.49	–0.561	Cytoplasmic
***PtOFP23***	Potri.014G181300.1	240	79	9.615	6.27	–0.229	Mitochondrial/Nucleus
***PtOFP24***	Potri.015G004300.1	312	103	11.439	4.99	–0.058	Nucleus
***PtOFP25***	Potri.001G180400.1	888	295	33.215	5.92	–0.758	Nucleus
***PtOFP26***	Potri.002G051200.1	771	256	28.035	5.13	–0.466	Nucleus
***PtOFP27***	Potri.006G205400.1	615	204	22.996	8.88	–0.726	Nucleus
***PtOFP28***	Potri.013G155300.1	561	186	21.989	9.84	–0.458	Mitochondrial
***PtOFP29***	Potri.019G128400.1	573	190	22.344	9.66	–0.348	Mitochondrial/Nucleus
***PtOFP30***	Potri.018G080100.1	879	292	33.683	9.74	–0.636	Nucleus

### Evolutionary Analysis and Microsynteny Analysis of PtOFP Genes

**FIGURE 1:**
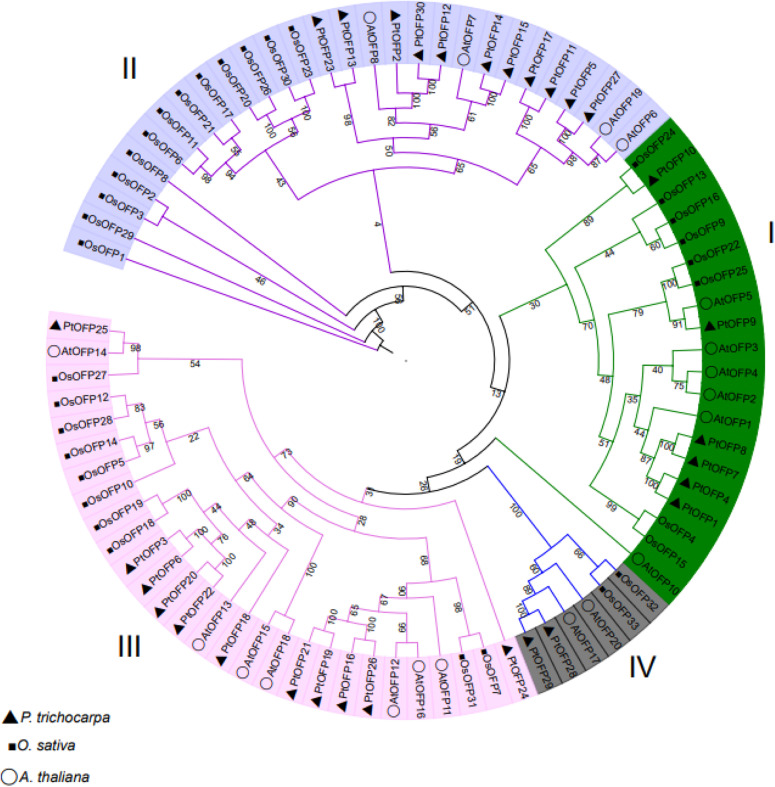
Phylogenetic analysis of OFPs in *A. thaliana, O. sativa*, and *P. trichocarpa. A. thaliana*, *O. sativa*, and *P. trichocarpa* OFPs were aligned using ClustalX, and the tree were constructed by using the neighbor-joining (NJ) method with MEGA 7.0. The tree was divided into four subfamilies according to bootstrap support values and evolutionary distances. The bootstrap analysis was performed using 1000 replicates.

PtOFP protein was subjected to phylogenetic analysis to examine their grouping pattern and genetic relationships using the full-length amino acid sequence of 30 PtOFPs, 33 OsOFPs, and 19 AtOFPs. As shown in Figure 1, PtOFPs were divided into four major subfamilies, designated as I–IV, corresponding to four groups in *Arabidopsis* as defined by Wang et al. (2011), which is also largely consistent with the phylogenetic relationship of OFPs in rice (Yu et al., 2015). Subfamily II and subfamily III were the two largest subfamilies, and both contained 11 *PtOFP* genes, whereas subfamily I and subfamily IV contained only six and two OFP members, respectively.

Microsynteny have been used to examine the evolutionary origins and orthologous relationships among plant species by their whole-genome sequences (Lin et al., 2014; Wang et al., 2015). In order to explore the molecular history of the chromosomal regions in which they reside, microsynteny analysis of two dicotyledons (*P. trichocarpa* and *A. thaliana*) and one monocotyledon (rice) was performed to clarify the relationship of the *OFP* genes between eudicots and monocots. OFP family members in these three species were used as anchor genes. Through pairwise comparisons of flanking genes in the chromosomal regions containing *OFP* genes, it was found that there were 30 pairs of *OFP* orthologous genes between *A. thaliana* and *P. trichocarpa* and 26 pairs between *O. sativa* L and *P. trichocarpa*, whereas 33 pairs of orthologous gene pairs were found between *A. thaliana* and *O. sativa* L (Supplementary Figure 2). These results implies that during species divergence, 33 rice *OFP* and 30 *P. trichocarpa OFP* genes were derived from *Arabidopsis*.

### Chromosomal Localization, Gene Structure, and Protein Motif Analysis

We mapped the 30 *PtOFP* genes onto the 19 chromosomes of *Populus* linkage groups (LG) and found that they are unevenly distributed on 15 of the 19 chromosomes. LG6 contained the largest number of *OFP* family genes (four genes), whereas the lowest number of *OFPs* was found on LG 1, 7, 14, and 15, which only contains one *PtOFP* gene in each of these chromosomes. In addition, three *PtOFP* genes were located in LG4 and LG16. No *OFP* family member gene was found on the four remaining *Populus* chromosomes including LG3, LG11, LG12, and LG17 (Supplementary Figure 3). The distribution of *PtOFPs* among the chromosomes was not uniform.

**FIGURE 2:**
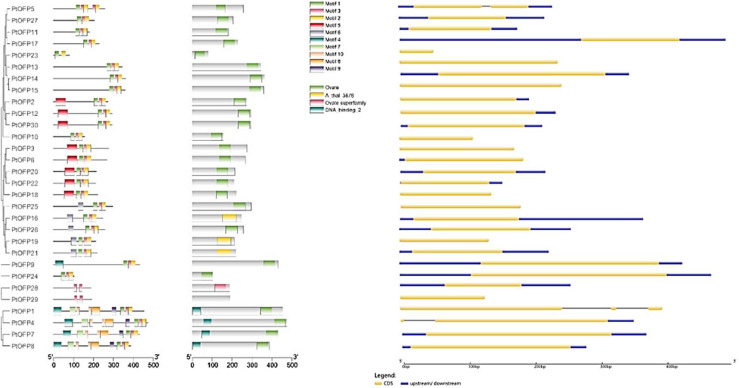
*PtOFP* schematic representation motifs and genes structure. The motifs were identified by online MEME. Different-colored boxes show different motifs (Motifs 1 to 10) and their position in each PtOFP sequence. For exon/intron organization of *PtOFP* genes, yellow boxes represent exons and black lines with the same length represent introns. The upstream/downstream regions of *PtOFP* genes are indicated in blue boxes. The length of exons can be inferred by the scale at the bottom.

In order to examine the gene structure of the *PtOFP* genes, we performed an analysis of the number and distribution of exon–intron and found that the majority of *PtOFP* genes (27/30) were intron-less. *PtOFP4* (Potri.016G134200) and *PtOFP5* (Potri.016G072800) each contains one intron, and *PtOFP1* (Potri.006G107700) has two introns (Figure 2). This result is consistent with the findings from other angiosperm species. For instance, seven members in the SlOFP family are intron-containing genes among 31 tomato *OFPs* (Huang et al., 2013). In addition, 32 of all the 33 *OsOFP* genes are intronless (Yu et al., 2015). Among the 15 peach *OFP* (*PpOFP*) genes, 14 of them did not contain introns (Li et al., 2019). No more than two introns are present in the intron-containing *OFPs* in *P. persica, Z. mays, O. sativa, S. lycopersicum, A. thaliana*, and *C. melo* (Yu et al., 2015).

To characterize the architecture of OFP proteins in the *P. trichocarpa*, motifs shared among the proteins within this family were analyzed by submitting all the PtOFP amino acid sequences to the MEME website. The detail motif logos and sequences are listed in Supplementary Figure 4. A total of 10 conserved motifs were identified and designated as motif 1 to motif 10 (Figure 2). Some motifs were common to most members, while the others were unique in one or few subclasses. For example, the common motifs distributed diffusely at the C-terminal are motifs 1 and 2, which were found in 28 out of 30 (93.3%) *P. trichocarpa* OFPs. Motifs 4, 9, and 10 were unique to subfamily I, whereas PtOFP28 and PtOFP29 only possess motif 3, which belong to subfamily IV. These results implied that PtOFP protein members within the same subfamily are likely to share similar function.

### PtOFP Genes Expression Patterns

**FIGURE 3:**
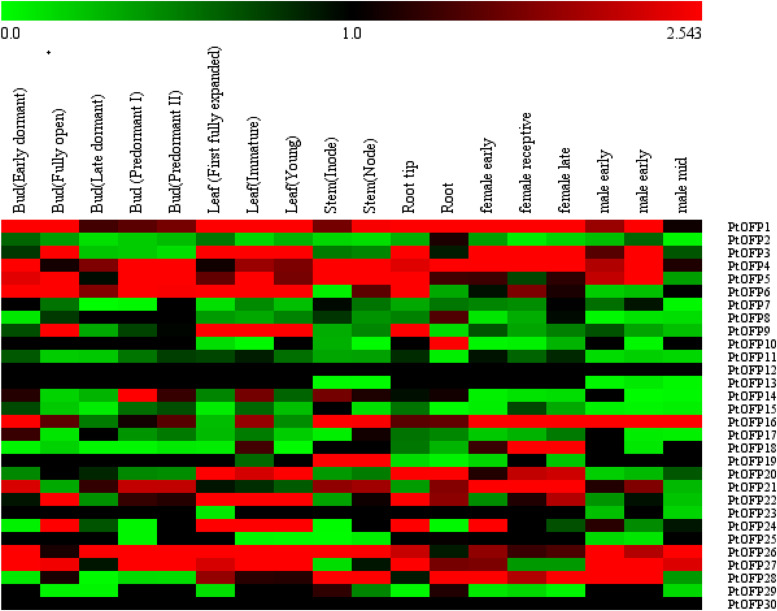
Heatmap of the expression levels of *PtOFP* family genes during different developmental stages in different tissues and organs. The tissue types are shown on the top, and the genes are shown on the right. Scale bars at the top represent log^2^-transformed FPKM values. Red indicates high expression level and green indicates low expression level.

To analyze the expression profiles of *PtOFPs* across tissues and organs and developmental stages, the expressed values of *PtOFP* genes were compiled from RNA-seq data in the *P. trichocarpa* Gene Atlas Study at Phytozome^1^. Normalized fragments per kilobase of transcript per million mapped reads (FPKM) values were compared to determine gene expression in different tissues. Then, we generated a heatmap image of 30 *PtOFP* genes collected from 18 different samples under standard conditions, which includes two root samples (root and root tip), two stem samples (internode and node), three leaf samples (immature, first fully expanded, and young), five bud samples (early dormant, fully open, late dormant, predormant I, and predormant II stage), female flower buds (early, receptive, and late), and male flower buds (early, receptive, and late). Half of *PtOFP* genes exhibited low (FPKM < 1) or undetectable expression (FPKM = 0) in the tested tissues or organs. Most *PtOFP* genes showed expression across all tissues. In addition, gene expression tends to be lower in reproductive tissues than in vegetative organs. A few *PtOFP* genes exhibited tissue-specific expression patterns. For example, *PtOFP18* (Potri.009G161600) was specifically expressed at the female reproductive stage (Figure 3).

Gene expression profiles could provide clues for functional studies. The Gene Atlas dataset analysis revealed that some *PtOFP* genes showed tissue-specific expression. Therefore, 16 *PtOFPs* whose gene expression level showed higher (FPKM > 2) in the root, stem and leaf from Gene Atlas Study data were chosen for qRT-PCR validation using gene-specific primers (Supplementary Table 1). As expected, *PtOFP5* and *PtOFP22* were detected across roots, leaf, and stems. *PtOFP1*, *PtOFP4*, and *PtOFP20* showed high expression levels in the primary root than in other tissues. Three genes (*PtOFP3, PtOFP9*, and *PtOFP24*) displayed higher expression in leaf than in other tissues (Supplementary Figure 5). Taken together, the results from the qRT-PCR analysis were largely consistent with the Gene Atlas RNAseq data.

### Analysis of *Cis*-Elements in the Promoter Regions and Hormone-Induced Expression Profiles of PtOFP Genes

Previous studies have shown that the expression of *OFP* genes responds to various plant hormone treatments (Liu et al., 2015; Schmitz et al., 2015; Xiao et al., 2017). Therefore, we wanted to determine whether there are plant hormone-related *cis*-acting elements within the promoter region of *PtOFP* genes. A genomic sequence 2000 bp upstream of the start codon of *PtOFP* genes was scanned to detect known *cis*-acting elements related to plant hormones. It has been shown that most *cis*-elements bound by TFs are typically present within 2000-bp upstream regions of the start codon of target genes (Fang et al., 2008).

As shown in Supplementary Figure 6, five hormone-responsive regulatory elements including ABRE, TGA-element, TATC-box, TGACG-motif, and TCA-element, associated with abscisic acid (ABA), auxin (IAA), gibberellin (GA), methyl jasmonate (MeJA), and salicylic acid (SA) responses, respectively, were identified in the promoter region of *PtOFPs*. Different types and numbers of regulatory elements were present in the promoter regions of individual *PtOFP* genes, implying that *PtOFP* genes may be involved in the response to various plant hormone treatments.

**FIGURE 4:**
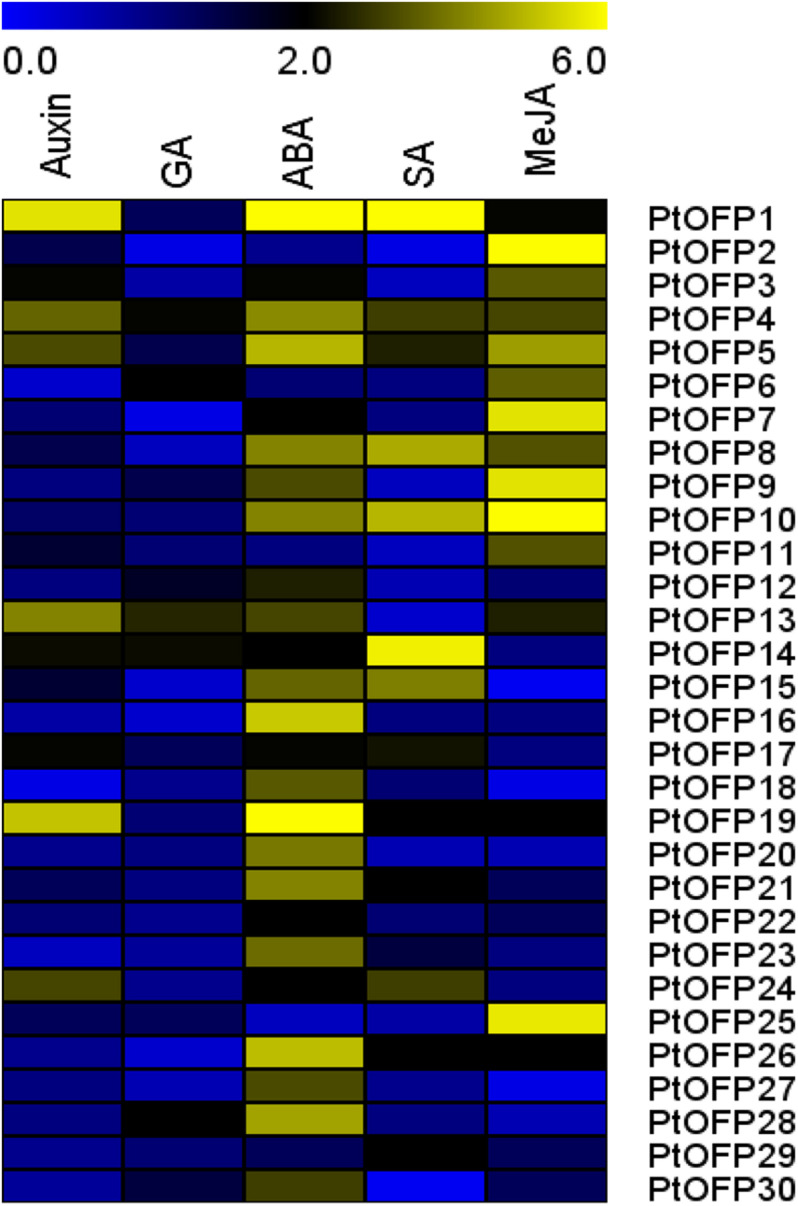
Expression profiles of the 30 *PtOFP* genes upon different plant hormone treatment. 717-1B4 *P. trichocarpa* seedlings were treated with 1 μM IBA, 1 μM GA3, 100 μM ABA, 5 mM SA, or 10 μM MeJA for 3 h. *PtUBC* was used as a reference gene. The relative expression levels were calculated using the 2^− ΔΔCt^ method. The heatmap was created using MEV. Color scale represents fold changes.

To further characterize the potential mechanism between *PtOFP* genes and hormone signaling, qRT-PCR was employed to examine the expression profile of each *PtOFP* gene in response to IAA, GA, ABA, MeJA, and SA. The results showed that for the members who belong to Class III (PtOFP28 and PtOFP29), except for *PtOFP28*, which were upregulated with ABA, they did not respond to the given hormone treatments. Other groups of *PtOFP* genes responded to at least one hormone treatment, but each gene illustrated different expression patterns (Figure 4). For example, few *PtOFP* genes showed obvious change when treated by GA, while most of the *PtOFP* genes were induced to different degrees under ABA treatment. Particularly, *PtOFP1* showed the highest expression level change under ABA treatment. For SA treatment, the transcript profiles of *PtOFP* genes showed the upregulation of *PtOFP1*, *4*, *5*, *8*, *10*, *14*, *15*, *17*, *19*, *21*, *24*, *26*, and *29*, and the rest of the *PtOFP* genes were inhibited or not significantly affected during the treatment period. After MeJA treatment, the expression of most *PtOFP*s from *PtOFP1* to *PtOFP11*, as well as *PtOFP13, 19, 25*, and *26* were remarkably upregulated. On the other hand, the gene expressions of *PtOFP15*, *PtOFP18*, and *PtOFP27* were almost undetectable.

Notably, we found that most gene expression in subfamily I (*PtOFP1, PtOFP4, PtOFP8, PtOFP7, PtOFP9*, and *PtOFP10*) were affected by multiple plant hormone treatments.

### PtOFP1 Functions as a Transcriptional Repressor

**FIGURE 5:**
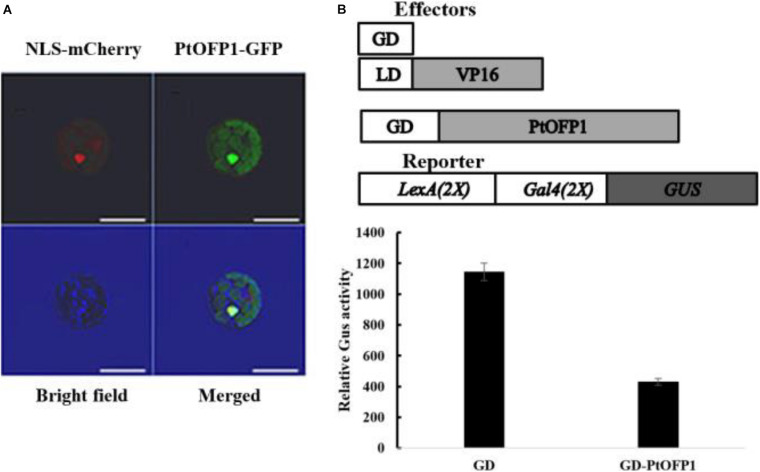
PtOFP1 is a transcriptional repressor. **(A)** Subcellular localization of PtOFP1. The nuclear marker NLS-mCherry was used as an indicator for the nucleus. Scale bars = 10 μm. **(B)** PtOFP1 transcriptional activity analysis. Plasmids of GD-PtOFP1 or GD alone (as a control) were co-transfected with a LexA-Gal4-GUS reporter into the protoplasts isolated from the *Populus* leaves. Transfected protoplasts were incubated in the darkness for 20–22 h before GUS activities were measured. Data represent mean ± SD of three replicates. Effectors and reporter used were diagrammed on the top of the figure.

A previous study has shown that OFP proteins can function as transcriptional repressors in *A. thaliana*, but little is known about molecular function and subcellular localization of OFP proteins in poplar. qRT-PCR analysis of the hormone-induction experiment above showed that 25 out of 30 *PtOFPs* were upregulated by ABA, among which, *PtOFP1* showed the highest expression upon ABA treatment (more than sevenfold). Because ABA is a plant stress hormone that plays an important role in drought stress response (Li et al., 2018), we hypothesized that PtOFP1 may play a role in drought stress response and selected PtOFP1 for further analysis. Therefore, we selected PtOFP1 (Potri.006G107700.1) as a representative to examine its subcellular localization. As shown in Figure 5A, PtOFP1 was localized at the nucleus.

To examine the transcriptional activity of PtOFP1 proteins, the *Populus* leaf mesophyll protoplast transient expression system (Guo et al., 2012) was used to assess the potential biochemical properties of PtOFP1. As shown in Figure 5B, co-transfection of the known LD–VP16 transactivator gene and an effector gene encoding only the Gal4 DBD (GD) resulted in activation of the GUS reporter gene, but when co-transfected with the GD–PtOFP1 effectors, the LexA-Gal4-GUS reporter showed a strong repression, indicating that similar to most AtOFPs, PtOFP1 can act as a transcriptional repressor.

### PtOFP1 Protein Interaction Networks

**FIGURE 6:**
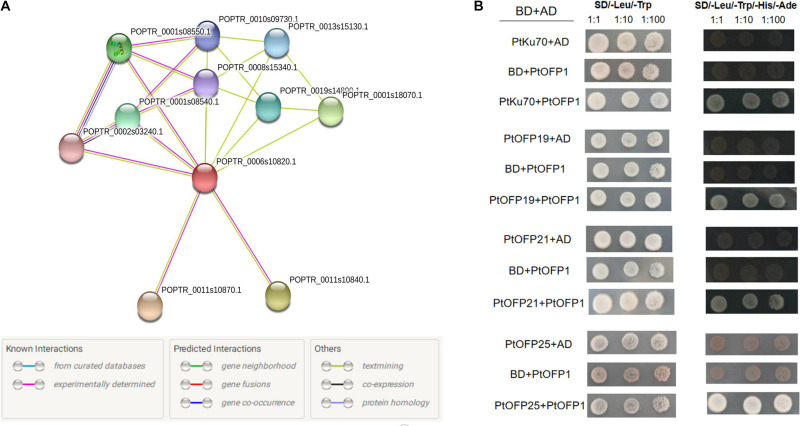
Functional protein association network and yeast two-hybrid assay. **(A)** Protein interaction network analysis revealed 10 potential proteins that may interact with PtOFP1. Line colors indicate the different kinds of evidence used to predict the network of protein interactions. **(B)** Yeast two-hybrid analysis of protein–protein interactions of PtOFP1 proteins. AD and BD represent empty pGADT7 and pGBKT7 vectors, respectively. SD/-Leu/-Trp represents the synthetic dextrose media (SD) lacking Leu and Trp. SD/-Leu/-Trp/Ade/-His/-Ade indicates SD medium lacking Leu, Trp, Ade and His.

Previous studies showed that the OFPs tend to interact with other proteins as a protein complex to regulate plant growth and development. In this study, the STRING software was used to find out potential proteins interacting with PtOFP1. The results are graphically represented in Figure 6A. The PtOFP1 protein was found to interact with 10 different proteins: six uncharacterized protein (POPTR_0011s10840.1, POPTR_0002s03240.1, POPTR_0001s08540.1, POPTR_0019s14890.1, POPTR_0001s 08550.1, and POPTR_0013s15130.1), three Ovate family proteins [POPTR_0008s15340.1(OFP19), POPTR_0001s18070.1(OFP25), POPTR_0010s09730.1(OFP21)], and Ku70 family protein (POPTR_0011s10870.1).

To verify the direct interaction of PtOFP1 with these predicted proteins, a yeast two-hybrid (Y2H) assay was performed via co-transformation of the pGADT7-PtOFP1 prey construct with full-length bait pGBKT7-PtOFP19, pGBKT7-PtOFP21, pGBKT7-PtOFP25, or pGBKT7-PtKu70-like family protein. Consistent with the protein–protein interaction prediction, PtOFP1 was found to interact with PtOFP19, PtOFP21, PtOFP25 and PtKu70 proteins (Figure 6B).

### Overexpression of PtOFP1 Enhances Drought Tolerance in *Arabidopsis*

We found that *PtOFPs* showed multiple responses to several plant hormones including IAA, GA, ABA, MeJA, and SA, implying that PtOFPs may participate in diverse biological processes. We also found that most *PtOFP* genes were responsive to ABA treatment, among which, *PtOFP1* showed the highest induction by ABA (Figure 4). Because ABA has been shown to play a critical role in regulating drought stress response, we wanted to further examine whether PtOFP1 plays a role in drought stress response. Therefore, we generated *35S:PtOFP1* transgenic *Arabidopsis* lines. Two independent transgenic lines (OE3-2 and OE3-7) that exhibited higher *PtOFP1* expression levels were selected for further analysis of their response to drought stress.

**FIGURE 7:**
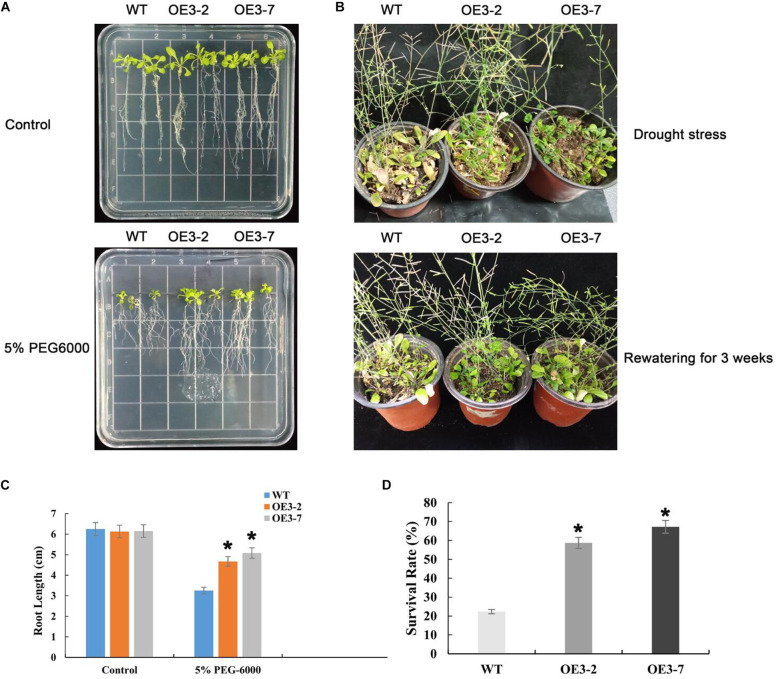
Drought stress responses of transgenic *Arabidopsis* overexpressing PtOFP1. **(A)** Phenotypes of WT and transgenic plants grown on PEG6000 (0 and 5%) for 10 days. **(B)** Phenotypes of drought treatment for 15 days and rewatering for 3 weeks. **(C)** Root length of WT and transgenic seedlings that were transferred to medium with or without 5% PEG-6000. **(D)** The survival rates of Col-0, OE3-2, and OE3-7 under drought stress. The results are shown as the means ± SD of three biological replicates. Asterisks indicate a significant difference at *P < 0.05 with respect to corresponding controls.

We first examined the drought tolerance at seedling stage. The growth of WT and transgenic *Arabidopsis* plants was not significantly different when grown on 1/2 MS medium without 5% PEG-6000. However, under PEG-induced drought stress for 10 days, WT seedlings exhibited severe inhibition of primary root growth compared with transgenic lines. The root lengths of the OE3-2 and OE3-7 transgenic plants were 4.67 and 5.08 cm, respectively, and average 34% longer than that of wild type (Figure 7A).

Meanwhile, drought tolerance responses under dehydration were also assessed by withholding water. Under normal growth conditions, all lines showed similar phenotypes. However, after 15 days of dehydration, the wilting frequency was 21.7–25.8% in transgenic lines compared within 60.3% in WT. Then, the stressed plants were re-watered following 21 days of dehydration. One day after of re-watering, 57.1–62.6% of the transgenic plants survived, while only 23.6% of the WT plants survived, indicating that the transgenic lines have significantly higher recovery frequency than that in WT (Figure 7B). Statistically significant differences were carried out using *post hoc* analysis (^∗^*P* < 0.05).

Collectively, PtOFP1 transgenic plants were more tolerant to drought than wild-type plants both at seedling stage and mature stage.

## Discussion

### Characterization of *OFP* Gene Family in *Populus*

The *OFP* gene family has been identified and characterized in various plants at the whole-genome level, including *A. thaliana* (19 members) (Liu et al., 2014; Wang et al., 2016), rice (33 members) (Yu et al., 2015), tomato (31 members) (Huang et al., 2013), and peach (15 members) (Li et al., 2019). Initially, it was thought that there were 18 AtOFP family members in *Arabidopsis* (Wang et al., 2011). Later on, AtOFP9 was removed from this family due to its new annotation. In addition, AtOFP19 and AtOFP20 were newly added as OFP family members in *Arabidopsis*. Therefore, there are a total of 19 *OFP* genes in *A. thaliana*. Liu et al. proposed that there were 29 *PtOFPs* in the *P. trichocarpa* genome by searching the keyword in the phytozome (v8.0) database (Liu et al., 2014). However, in this study, a new member, Potri.018G080100, was identified from the version 12.0 of phytozome^1^, which was named PtOFP30.

To further examine the evolutionary relationship of *OFP* genes in the *P. trichocarpa*, we constructed a phylogenetic tree including OFP proteins from *A. thaliana*, *O. sativa*, and *P. trichocarpa*. PtOFPs were divided into four major subfamilies (Figure 1), which is consistent with that in *A. thaliana* and rice (Wang et al., 2011; Yu et al., 2015). It is worth noting that AtOFPs members who are categorized into the same subfamily are likely to share similar phenotypes when overexpressed. For example, overexpression of Class I members (AtOFP1, AtOFP2, AtOFP4, AtOFP5, and AtOFP7) resulted in kidney-shaped cotyledons, as well as round and curled leaves. The leaves of overexpression plants of Class II AtOFP6 and AtOFP8 transgenic line are flat, thick, and cyan, and overexpression of Class III (AtOFP13, AtOFP15, AtOFP16, and AtOFP18) led to another distinct phenotype including blunt end siliques. Overexpression plants of all other AtOFPs examined were shown to be undistinguished from wild type (Liu et al., 2014).

Gene organization plays a vital role in the evolution of multiple gene families (Chen G. et al., 2018). In the present study, gene structure analysis revealed that most *OFP* genes in the *P. trichocarpa* are intron-less, which is consistent with the findings from other angiosperm species. For instance, among 31 *OFPs* in tomato, eight members in SlOFP family contain introns (Huang et al., 2013). Among the 15 peach *OFP* (*PpOFP*) genes, 14 of them did not contain introns (Li et al., 2019). As for rice, only *OsOFP14* (LOC_Os04g33870.1) contains intron within all the 33 *OsOFP* members. No more than two introns are present the intron-containing *OFPs* in *P. persica, Z. mays, O. sativa, S. lycopersicum, A. thaliana*, and *C. melo* (Yu et al., 2015).

We also analyzed the motif compositions of OFPs in the *P. trichocarpa* (Figure 2). Overall, most of the PtOFPs contained motif 1, motif 2, and motif 3. Additionally, although the motif may be different in individual PtOFPs, the motif composition within the same subgroup tends to be similar. For example, motifs 4, 9, and 10 were unique to subfamily I, whereas PtOFP28 and PtOFP29 only possess motif 3, which belongs to subfamily IV. These results suggested that the motif compositions are closely related to the members grouping in the phylogenetic tree.

### *PtOFP* Genes Play Crucial Roles in Response to Phytohormone

As aforementioned, 50% of *PtOFP* genes were expressed at undetectable or low level in tested leaf, stem, and root under normal conditions, implying that these *PtOFPs*’ expression may depend on biotic or abiotic stimuli. Increasing evidence indicated that OFP proteins play vital roles in the regulation of gene expression in response to adverse environmental conditions (Wang et al., 2007). Phenotypic analysis showed that overexpressing *Arabidopsis* Class III *OFP* genes caused blunt-ended siliques, which is similar to that in *er* mutants. A subsequent study revealed that Class III AtOFPs (OFP15, OFP16, and OFP18) may be phosphorylated by kinases downstream of the ER signaling pathway (Wang et al., 2019). In terms of horticultural crops, the banana OFP1 (MaOFP1) interacted with a MADS-box protein MuMADS1 to modulate ethylene-prompted postharvest maturation (Liu et al., 2018). However, no report of involvement of any *PtOFP*s in hormone treatment has been documented to date, which has led us to examine their potential roles in response to various hormones. In the present studies, ABRE, TGA-element, TATC-box, TGACG-motif, and TCA-element were found to distribute in the promoter regions of *PtOFP* genes (Supplementary Figure 6), indicating that a number of *OFP* genes in *P. trichocarpa* may participate in various hormone-related processes including IAA, GA, ABA, SA, and MeJA. Furthermore, the expression profiles of *PtOFPs* responding to exogenous phytohormone mentioned above were investigated (Figure 4). qRT-PCR analysis showed that few *PtOFP* genes showed altered transcript level treated by GA. However, prior reports have indicated that AtOFP1 and CaOvate function as active transcriptional repressor in the GA biosynthesis pathway by negatively affecting the expression of *AtGA20ox1 or CaGA20ox1* (Wang et al., 2007; Tsaballa et al., 2011). In addition, overexpression of OsOFP2 (LOC_Os01g43610) in rice alters leaf morphology and seed shape by downregulating the expression of OsGA20ox7 to suppress the GA level (Schmitz et al., 2015). These results implied that *OFP* genes in *P. trichocarpa* may exhibit different expression or induction patterns from that in non-woody plants, or PtOFPs are likely to be induced by different GA treatment concentration or period of time.

It has been well documented that ABA serves as a critical signaling factor in response to drought stress, and ABA can control the water status of plant via stomatal conductance and inducing the expression of genes involved in dehydration response. ABA can improve drought resistance by inducing plant antioxidant defense system and suppressing ROS damages (Desikan et al., 2004; Sharp et al., 2004). Additionally, ABA can activate certain antioxidant enzymes, regulate the osmotic adjustment, and improve the hydraulic conductivity of roots by changing gene expression of aquaporin family. Previous studies have also demonstrated the relationship between ABA hypersensitivity and enhanced drought tolerance. For example, WRKY68 TF in cotton (GhWRKY68) has been shown to improve the performance of transgenic plants under drought stress via an ABA-dependent signaling pathway (Hu et al., 2010). In our study, the expression of *PtOFP1* was highly induced by ABA treatment (Figure 4), and the PtOFP1 overexpression lines had improved drought stress tolerance compared to WT (Figure 7), suggesting that PtOFP1 is likely involved in an ABA-dependent signaling pathway in responses to drought stress. In future studies, it would be interesting to investigate how PtOFP1 interacts with known regulators in the ABA-dependent signaling pathway regulating drought stress response.

Additionally, it has been illustrated that SlOFP20 may play an important role in the crosstalk between BR and GA (Zhou et al., 2019). Interestingly, the present study showed that the transcripts of *PtOFP8* and *PtOFP14* were increased after treated with SA and MeJA, implying that these two PtOFP proteins may be involved in the crosstalk between SA and MeJA (Figure 4). Collectively, these findings suggest that *PtOFP* members might play diverse roles in sensing multiple plant hormone signals for *Populus* to adapt to variable stresses.

### PtOFP Identification and Function

Transcription factors play important roles in diverse biological processes in plant growth, development, and stress responses (Zhang T. et al., 2018; Sun et al., 2019). A previous study has shown that AtOFP proteins serve as transcriptional repressors in *Arabidopsis*, but little is known about the molecular function of OFP proteins in poplar. In this study, we used subcellular localization and transcriptional activity analysis and found that PtOFP1 (Potri.006G107700) was localized in the nucleus and that it can act as a transcriptional repressor.

Transcription factors often work together with other proteins to regulate the transcription of downstream targets (Chen H. et al., 2018; He et al., 2018). A previous study showed that most OFPs family members contain a predicted nuclear localization signal but lack the recognizable DNA binding domains (Hackbusch et al., 2005; Wang et al., 2007), implying that OFPs are inclined to interact with other proteins (i.e., KNOX and BLH) as protein functional complexes to mediate plant development and growth. For instance, the development of secondary cell walls of *Arabidopsis* and cotton are related to the AtOFP1, AtOFP4, and KNAT7–BLH6 complex and to the GhOFP4 and GhKNL1 heterodimer, respectively (Gong et al., 2014; Liu and Douglas, 2015). AtOFP1 regulates the transition timing from vegetative growth to reproductive growth by interacting with BLH3 (Zhang et al., 2016). AtOFP5 could regulate early embryo sac development by repressing the activity of a BLH1–KNAT3 complex (Pagnussat et al., 2007). Although Y2H assays have been used in studying many plant proteins such as those from *Arabidopsis*, banana, cotton, and peach to investigate OFPs protein–protein interactions, application of this technique has rarely been reported in the *P. trichocarpa* (Hu et al., 2010; Li et al., 2011, 2019; Liu et al., 2015). Firstly, prediction of protein interaction networks revealed that within the 10 potential PtOFP1-interacting proteins, three PtOFP family proteins and one PtKu70 protein were included. Interestingly, PtKu70 is the homologous AtKu70 (At1g16970), which was shown to interact with AtOFP1 (Wang et al., 2010). Then, Y2H assay was conducted to further verify the predicted protein interactions. In particular, three PtOFP proteins (PtOFP19, PtOFP21, and PtOFP25), as well as PtKu70 were validated to interact with PtOFP1 (Figure 6B). These results suggest that PtOFP1 protein can form protein complex with other proteins to regulate plant growth and development.

*OFP* genes in different plant species have been reported to participate in the response to various abiotic stresses, especially drought (Muthusamy et al., 2017). For example, several drought-responsive marker gene expressions were significantly higher in TaOFP29a-A-transgenic plants than in the WT (Wang et al., 2020). In addition, under PEG6000-induced osmotic stress conditions, the transgenic plants had longer roots than WT plants, and dry root biomass of the transgenic plants were significantly greater than wild type under water deficiency conditions. For OFP6 from *O. sativa*, the overexpression line showed slower water loss and less accumulation of H_2_O_2_ compared with RNAi plants under drought conditions, implying that OsOFP6 may confer both drought avoidance and drought tolerance in rice plants (Ma et al., 2017). To examine the potential function of PtOFP in drought stress, we generated PtOFP1 overexpression lines in *Arabidopsis*. As shown in Figure 7, PtOFP1 overexpression transgenic plants not only displayed drought tolerance at seedling stage but also showed significantly higher recovery frequency at the mature stage. Whether PtOFP1 and other PtOFP members are involved in other stresses such as cold or salt still requires further study.

Taken together, these results serve as the theoretical basis for understanding the biological function and regulation of poplar OFP proteins.

## Materials and Methods

### Genome-Wide Identification of PtOFP Genes

To search for OFP sequence homologs in the *P. trichocarpa*, an HMM profile of the OVATE domain (PF04844) was downloaded from Pfam^2^. Initially, 19 full-length amino acid OFP protein sequences in *Arabidopsis* collected from TAIR^3^ were used as queries by using BLASTP searches^4^. Putative OFP sequences were filtered based on an *E* value of ≤ 1 × 10^–10^. Secondly, each identified hit was used as a new query to conduct a BLAST search querying against the *P. trichocarpa* assembly genomic sequence, to ensure that no related genes were missed from the search. The searches were manually checked and run repeatedly until no new candidate was found.

The basic physical and chemical properties of each OFP protein sequence including molecular weight (Mw), isoelectric points (pI), and grand average of hydropathicity (GRAVY) were calculated using the ProParam tool in ExPASy program^5^ (Artimo et al., 2012). The online CELLO system^6^ (Yu et al., 2004) was used to predict subcellular localization.

### Sequence alignment, Phylogenetic Analysis, and Microsynteny Analysis

The full-length sequences of OFPs from *A. thaliana* and rice downloaded from the phytozome database^7^, together with newly identified PtOFPs, were used for phylogenetic analysis.

Multiple alignments for all the acquired and predicted OFP full-length protein sequences were reciprocally aligned by ClustalX2 software with the default parameters. A phylogenetic tree was inferred using the Neighbor Joining (NJ) method of MEGA 7.0 (Kumar et al., 2016). Bootstrap tests were performed with 1000 replicates for a statistical reliability analysis.

A BLAST search against the whole genomes of *A. thaliana*, *O. sativa*, and *P. trichocarpa* was used to investigate the microsyntenic relationships of *OFP* genes among these species. The results were displayed using Circos software^8^ (Krzywinski et al., 2009).

### Chromosomal Distribution, Gene Structure, and Protein Motifs Analysis

To determine the corresponding *OFP* gene loci across the *P. trichocarpa* chromosomes, the annotated genetic locations of PtOFPs were obtained from the PopGenIE database^9^. MapInspect tool^10^ software was used for creating the map of *PtOFP* genes’ physical chromosomal positions and relative distances proportionally.

For gene structure analysis, the exon/intron structures of individual *OFP* genes were illustrated using the Gene Structure Display Server (GSDS^11^) by aligning the genomic DNA sequences with the corresponding cDNA sequences (Supplementary Tables 2, 3) from the JGI^12^ database.

The conserved motifs in the putative PtOFP proteins were identified by Multiple Expectation Maximization for Motif Elicitation (MEME) online program^13^ (v4.12.0) (Bailey et al., 2009). MEME was run locally with the following parameters: the default settings for motif width (between 6 and 50 wide) and site distribution (zero or one occurrence per sequence), with the maximum number of motifs = 10. Amino acid sequences of PtOFPs are shown in Supplementary Table 4.

### Promoter *Cis*-Element Identification

To identify the cis-acting elements in the promoter region, upstream sequence (2.0 kb) relative to the translation start codon in each *PtOFP* gene was downloaded from Phytozome 11.0 and then submitted to PlantCARE databases^14^ (Lescot et al., 2002) to identify representative regulatory elements including ABRE (abscisic acid-responsive elements), involved in the ABA responsiveness; TCA-element, involved in SA responsiveness; TGACG-motif, involved in the MeJA-responsiveness; TATC-box, involved in GA responsiveness; and TGA-element, involved in the auxin-responsive element. The sequences of the *PtOFP* promoters are listed in Supplementary Table 5.

### Plant Materials and Growth Conditions

*Populus tremula* × *Populus alba* (*Populus* clone 717-1B4) was used for all experiments in this study. Fresh tissues were excised from greenhouse for plant propagation in media after surface sterilization. *Populus* tissues were sterilized in 1% (v/v) Tween-20 solution for 5 min, then 70% (v/v) ethanol for 1 min, and 15 min in 10% (v/v) bleach, followed by triple rinsing for 5 min in sterile water. The plants were then transferred to GA-7 (Magenta boxes) and cultured in 100 ml of MS solid medium (pH = 5.7) containing 1% (w/v) sucrose and 0.5 g MES and solidified with 0.8% (w/v) agar. Plants were cultivated in a growth chamber at 25°C with a 24-h photoperiod.

For the plant hormone treatment, “717-1B4” seedlings were soaked in liquid MS medium and applied with 1 μM IBA, 1 μM GA_3_, 100 μM ABA, 5 mM SA, or 10 μM MeJA for 3 h. Samples collected from untreated plantlets were used as controls. Treated materials from three separate individual plants were combined and to be considered as one sample, and all the data shown are from a representative experiment of three independent times.

To generate *Arabidopsis* transgenic plants that constitutively express the *PtOFP1* gene, the *PtOFP1* coding sequence was cloned into the pBI121 overexpression vector under the control of the CaMV 35S promoter. Five-week-old *Arabidopsis* plants were used for transformation via *Agrobacterium tumefaciens* (strain GV3101)-mediated floral dip method. T1 seeds were planted on 1/2 MS medium with kanamycin (50 μg/ml) for selecting transgenic plants, and to confirm in T2 up to T3 generations, and two independent homozygous lines (OE3-2 and OE3-7) were chosen for further study.

For the phenotypic analysis, seeds of homozygous T3 and WT plants were sterilized and grown on 1/2 MS (10 × 10 cm) plates with/without 5% PEG-6000. The length of the primary roots was measured after 10 days, with each treatment containing three independent replicates. For imposing dehydration stress, 1-month-old *Arabidopsis* plants kept at 22°C at a 16-h light/8-h dark photoperiod were given dehydration stress by withholding water. Wilting frequency was measured after 15 days of dehydration treatment. Watering was resumed after 21 days of dehydration, and the number of survival plants were recorded the next day.

### RNA Extraction and qRT-PCR Analysis

The expression levels of *PtOFPs* in different tissues were extracted from the RNA-seq data on phytozome (see footnote 8). *OFP* gene with an FPKM > 1 were used for further expression analysis. The expression profiles were generated by using Mev4.6.2 software^15^.

To validate the expression of *PtOFPs* reported in the Gene Atlas Study^16^, qRT-PCR was performed with gene-specific primers for 10 *PtOFP* genes and the *PtUBC* was used as an internal control. Total RNA was extracted from selected tissues with Spectrum Total Plant RNA extraction kit (Sigma-Aldrich, St. Louis, MO, United States) following the manufacturer’s protocol. One microgram of total RNA was used to synthesize complementary DNA (cDNA) by reverse transcription with RevertAid Reverse Transcriptase (Thermo Fisher Scientific, Waltham, MA, United States). Two hundred nanograms of reversely transcribed cDNA was used to perform qRT-PCR reaction with gene-specific primers. qRT-PCR experiment with three replicates was performed on a Maxima SYBR Green PCR Master Mix (Thermo Fisher Scientific). The thermal cycling conditions were as follows: an initial denaturation step of 10 min at 95°C, followed by 40 cycles of 15 s at 95°C for denaturation, 30 s at 60°C for annealing, and 30 s at 72°C for extension. Then, the melting curve analysis was performed. The relative expression levels of genes were calculated using the 2^–^^Δ^^Δ^^CT^ method. *T*-tests were employed for statistical analyses. All primers used in this study including primers for gene cloning and primers for gene expression analysis was listed in Supplementary Table 6.

### Subcellular Localization and Transcriptional Activity Analysis of PtOFP1 Protein

PtOFP1 was selected to examine its subcellular localization. The corresponding full-length coding sequence was cloned into a pENTR Gateway entry vector (pENTR-D-TOPO, Invitrogen). This recombined cloning system was subsequently used to further subclone PtOFP1 coding sequences into the GFP-fused destination vectors. The PtOFP1-GFP fusion proteins were expressed under the control of the CaMV 35S promoter in *Populus* protoplasts, together with the nuclear marker m-cherry fluorescent protein. GFP fluorescence was visualized with a confocal laser scanning microscope.

To generate the *35S:GD-PtOFP*1 constructs, the full-length open-reading frame (ORF) of *PtOFP1* gene was amplified by PCR using cDNA isolated from 2-month-old micropropagated clone 717-1B4 (female, *P. tremula* × *P. alba*). The expression vectors were constructed as follows: the coding sequences of the *PtOFP1* were cloned into an entry vector (pENTR-D-TOPO, Invitrogen), according to the manufacturer’s instructions, and subsequently cloned into the destination vector by an LR reaction (Gateway recombination, Invitrogen). 35S promoter was used in all fusion constructs.

### *In silico* PtOFP1 Protein Interaction Prediction and Y2H Assays

The functional protein–protein interaction networks were generated by submitting the PtOFP1 protein sequences to the STRING computer service^17^. Then, Y2H assays were used to verify the predicted interacted proteins.

Y2H assay was performed according to the manufacturer’s instructions of MATCHMAKER GAL4 Two-Hybrid System (Clontech^18^). PtOFP1 were ligated into vector pGBKT7, whereas PtKu70, PtOFP19, PtOFP21, and PtOFP25 were each ligated into vector pGADT7. Different combinations of Gal4 binding with activation domain vectors were transformed into the AH109 yeast strain and were viewed on plates containing synthetically defined (SD) medium without leucine and tryptophan. After incubating at 30°C for 72 h, colonies were picked and cultured in the same liquid medium at 30°C for 16 h. Successfully transformed yeast cells were selected and tested on SD media lacking Leu, Trp, adenine, or His (SD/-Leu/-Trp/-Ade/-His).

### Statistical Analysis

Tests of statistical significance were performed using one-way ANOVA with *post hoc* analysis. Differences were considered significant if the *p*-value was < 0.05.

## Data Availability Statement

The original contributions presented in the study are included in the article/Supplementary Material, further inquiries can be directed to the corresponding author/s.

## Author Contributions

YC and J-GC conceived and designed the experiments. HW performed the experiments, analyzed the data, and drafted the manuscript. HW, YC, and J-GC revised the manuscript. All the authors read and approved the final manuscript.

## Conflict of Interest

The authors declare that the research was conducted in the absence of any commercial or financial relationships that could be construed as a potential conflict of interest.

## Footnotes

1 https://phytozome.jgi.doe.gov/pz/

2 http://pfam.xfam.org/

3 https://www.arabidopsis.org/

4 https://phytozome.jgi.doe.gov/pz/portal.html#!search?show=BLAST&method=Org_Ptrichocarpa

5 http://web.expasy.org/compute_pi/

6 http://cello.life.nctu.edu.tw/cello.html

7 https://phytozome.jgi.doe.gov/

8 http://circos.ca/

9 http://popgenie.org/gp

10 http://mapinspect.software.informer.com/

11 http://gsds.cbi.pku.edu.cn/

12 https://genome.jgi.doe.gov/portal/

13 https://meme-suite.org/meme/tools/meme

14 http://bioinformatics.psb.ugent.be/webtools/plantcare/html/

15 https://sourceforge.net/projects/mev-tm4/files/mev-tm4/

16 https://phytozome.jgi.gov/pz/portal.html

17 http://string-db.org/

18 http://www.clontech.com/

## Supplementary Material

Supplementary Figure 1 Amino acid sequence alignment of *Populus* OVATE domain.

Supplementary Figure 2 Microsynteny analyses of *OFP* genes among *P. trichocarpa*, *O. sativa*, and *A. thaliana.*

Supplementary Figure 3 Chromosomal distribution of *PtOFP* genes in the genome of *Populus trichocarpa*.

Supplementary Figure 4 The detail motif logos of PtOFPs.

Supplementary Figure 5 Real-time PCR analysis of 16 *PtOFP* genes in three *Populus* tissues (root, stem, and leaf). *PtUBC* was used as an internal control. The 2^–ΔΔCt^ method was used to calculate the relative expression levels of the target genes. The error bars indicate the standard deviation obtained from three replicates.

Supplementary Figure 6 Distribution of hormone-responsive elements in the promoter region of *PtOFP* genes.

Supplementary Table 1 All primers used in this study.

Supplementary Table 2 The genomic sequences of *PtOFP* genes.

Supplementary Table 3 The CDS sequences of *PtOFP* genes.

Supplementary Table 4 The Protein sequences of PtOFPs.

Supplementary Table 5 The promoter sequences of *PtOFP* genes.

Supplementary Table 6 List of *PtOFP* gene expression in 18 different samples. RNA-seq data were collected from the *Populus* Gene Atlas Study in Phytozome v11.0 (http://phytozome.jgi.doe.gov/pz/portal.html).

## Abbreviations

Pt: *Populus trichocarpa*
At: *Arabidopsis thaliana*
OFP: ovate family protein
aa: amino acid
bp: base pair
MW: molecular weight
ORF: open-reading frame
pI: isoelectric points
qRT-PCR: real-time quantitative reverse transcription PCR
ABREs: ABA-responsive elements
GSDS: gene structure display server
FPKM: fragments per kilobase of transcript per million fragments mapped.
