# Supplementary figures and images for: Identification, Expression, and Interaction Analysis of Ovate Family Proteins in Populus trichocarpa Reveals a Role of PtOFP1 Regulating Drought Stress Response
Source: Front Plant Sci. 2021 Apr 20;12:650109. doi: 10.3389/fpls.2021.650109 (PMC8095670; doi:10.3389/fpls.2021.650109)

## Slide 1
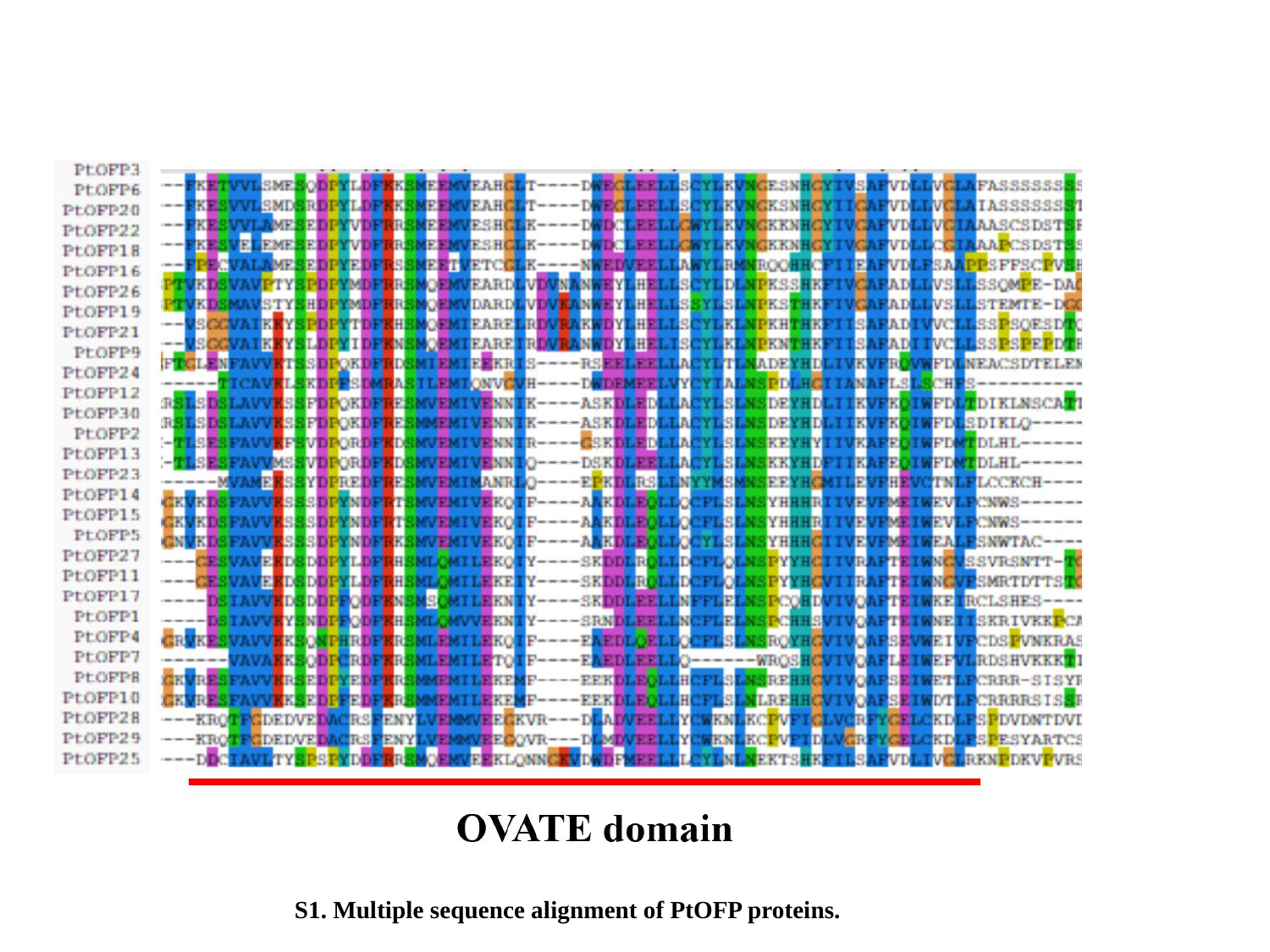

S1. Multiple sequence alignment of PtOFP proteins.

Supplement: Supplementary Figure 1 — Amino acid sequence alignment of Populus OVATE domain. [file Presentation_1.PPT]

## Slide 1
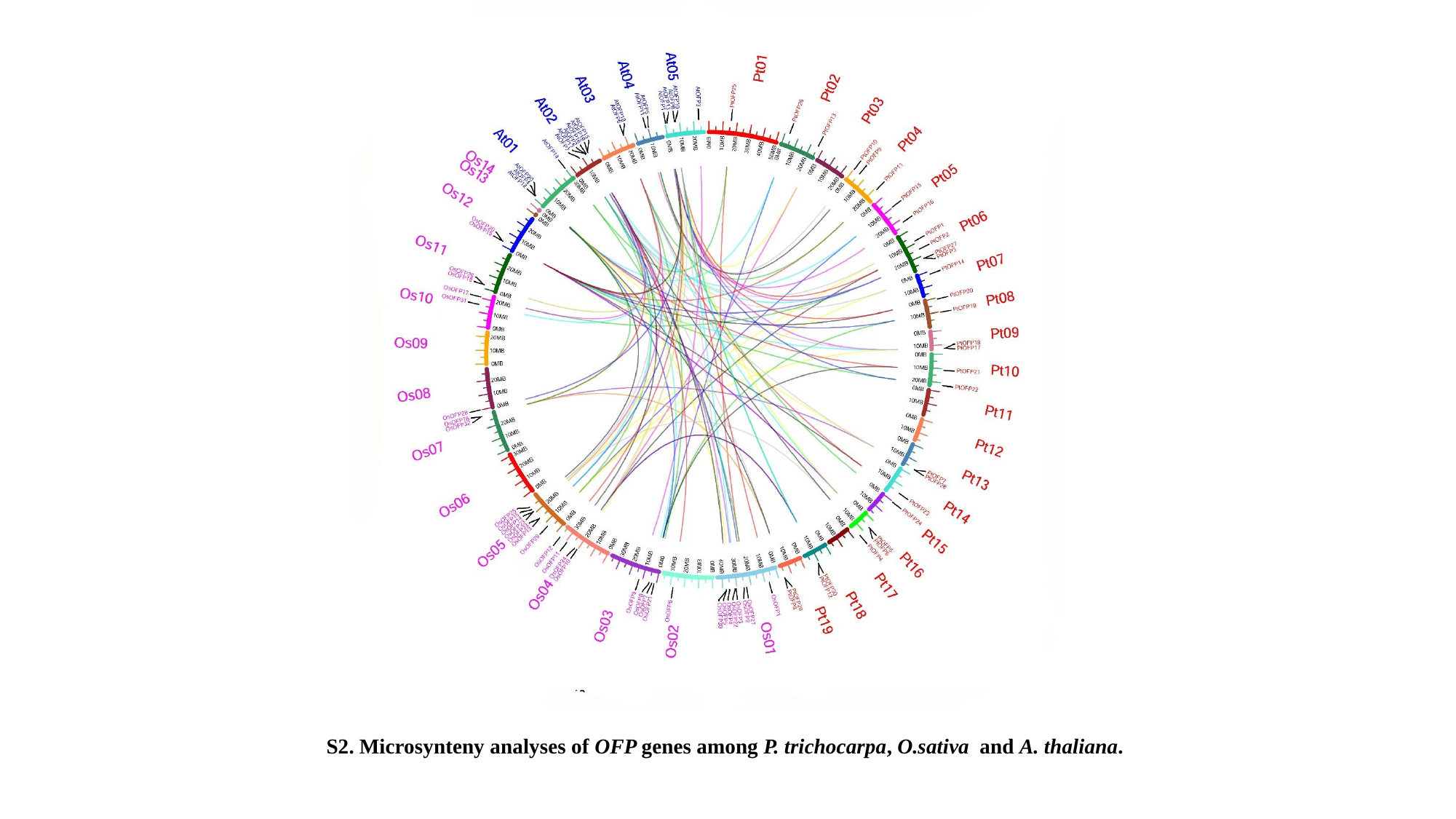

S2. Microsynteny analyses of OFP genes among P. trichocarpa, O.sativa  and A. thaliana.

Supplement: Supplementary Figure 2 — Microsynteny analyses of OFP genes among P. trichocarpa, O. sativa, and A. thaliana. [file Presentation_2.PPTX]

## Slide 1
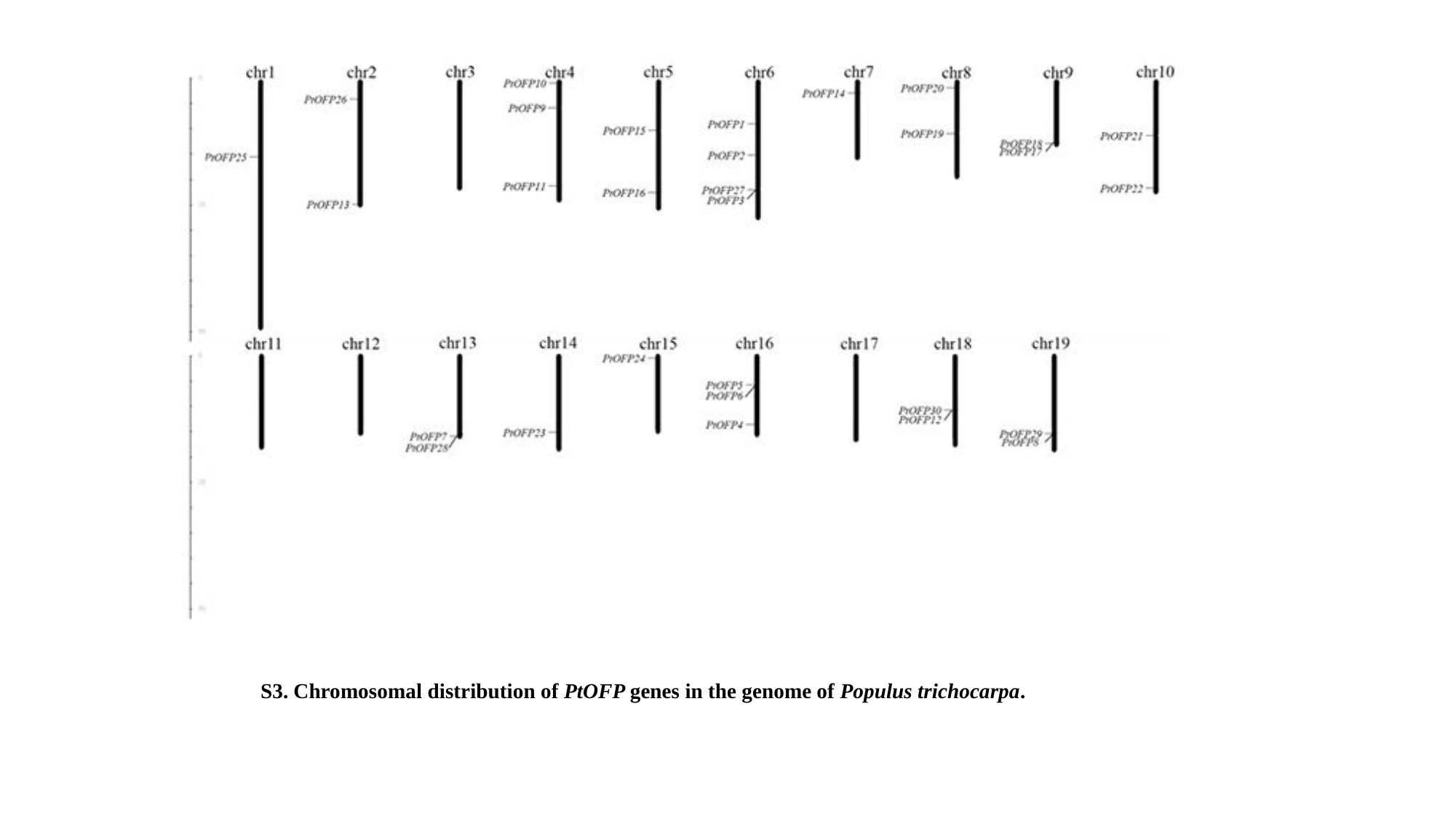

S3. Chromosomal distribution of PtOFP genes in the genome of Populus trichocarpa.

Supplement: Supplementary Figure 3 — Chromosomal distribution of PtOFP genes in the genome of Populus trichocarpa. [file Presentation_3.PPTX]

## Slide 1
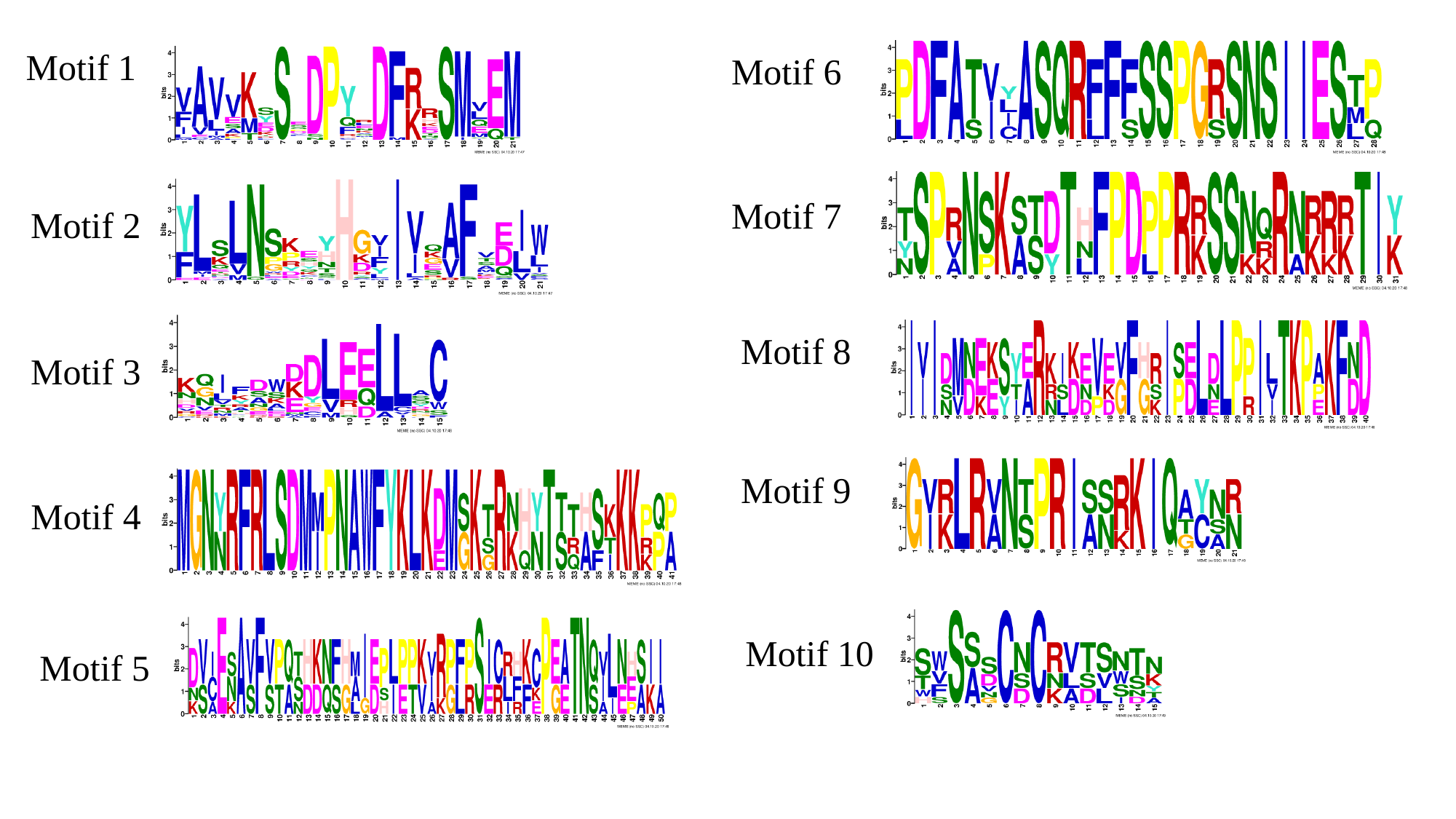

Motif 1
Motif 6
Motif 7
Motif 2
Motif 8
Motif 3
Motif 9
Motif 4
Motif 10
Motif 5

Supplement: Supplementary Figure 4 — The detail motif logos of PtOFPs. [file Presentation_4.PPTX]

## Slide 1
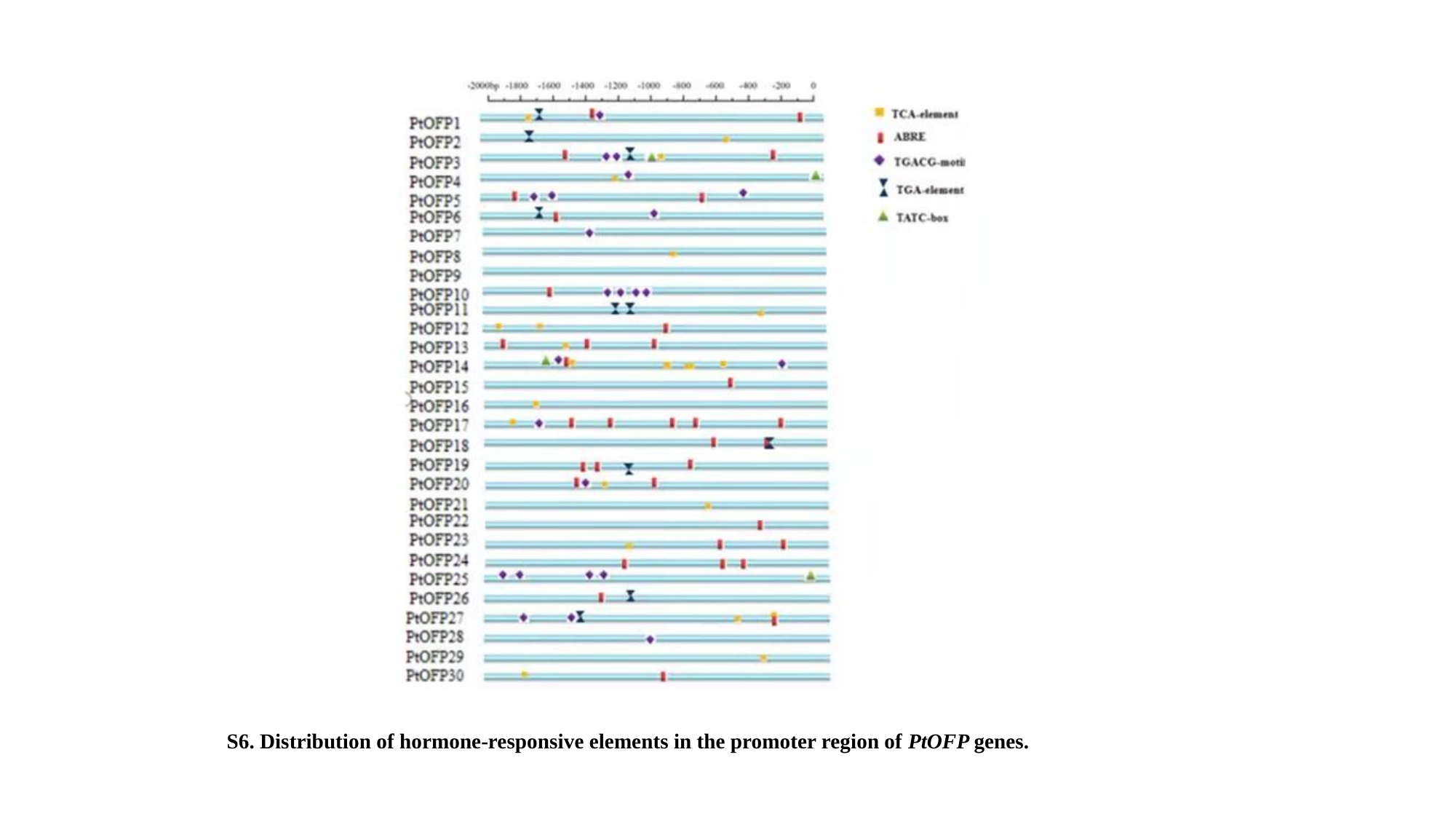

S6. Distribution of hormone-responsive elements in the promoter region of PtOFP genes.

Supplement: Supplementary Figure 6 — Distribution of hormone-responsive elements in the promoter region of PtOFP genes. [file Presentation_6.PPTX]
