# Supplementary material for: Identification, Expression, and Interaction Analysis of Ovate Family Proteins in Populus trichocarpa Reveals a Role of PtOFP1 Regulating Drought Stress Response
Source: Front Plant Sci. 2021 Apr 20;12:650109. doi: 10.3389/fpls.2021.650109 (PMC8095670; doi:10.3389/fpls.2021.650109)
Supplement: Supplementary Figure 5 — Real-time PCR analysis of 16 PtOFP genes in three Populus tissues (root, stem, and leaf). PtUBC was used as an internal control. The 2–ΔΔCt method was used to calculate the relative expression levels of the target genes. The error bars indicate the standard deviation obtained from three replicates. [file Presentation_5.PPTX]

## Slide 1
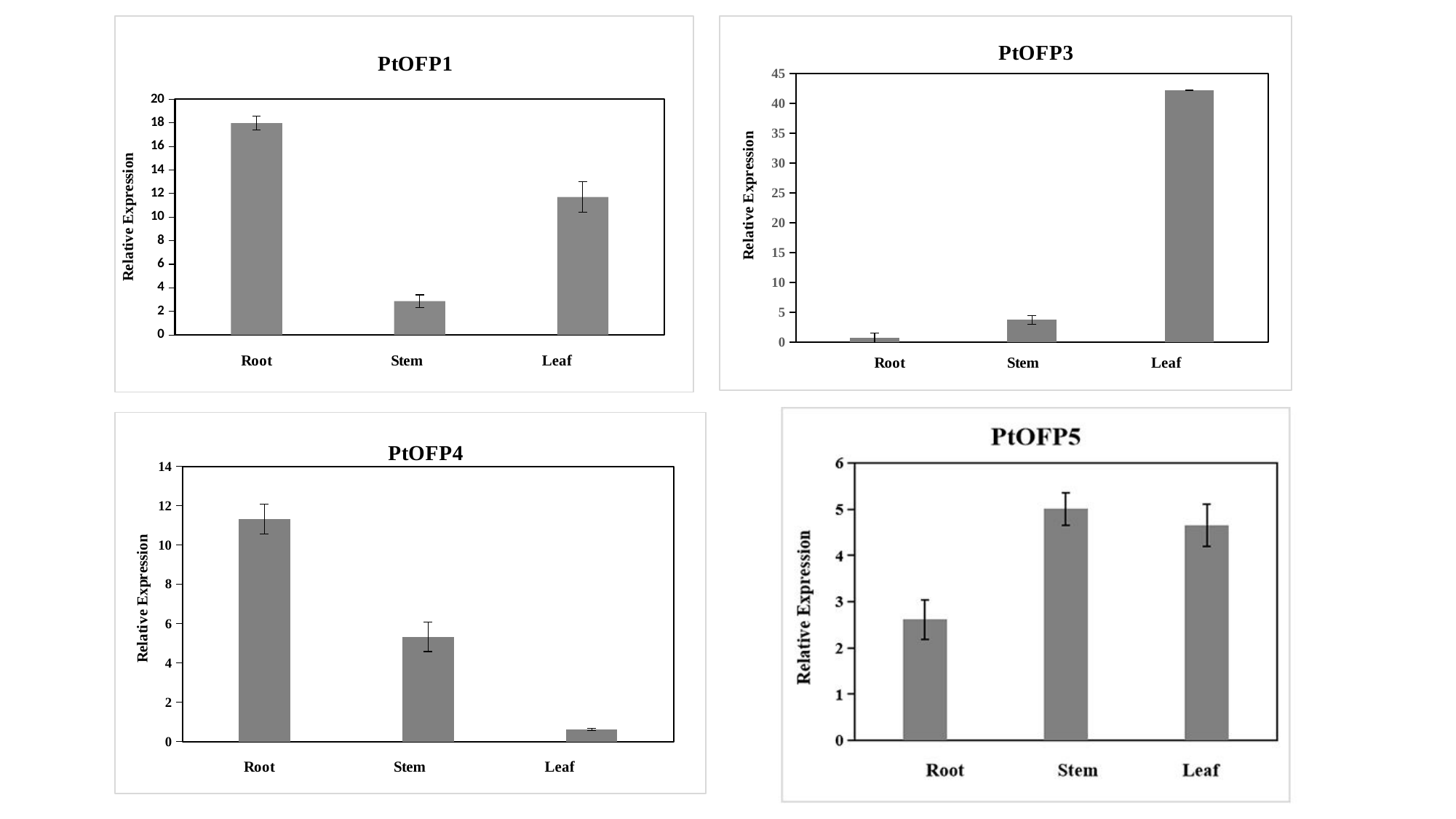

### Chart: PtOFP1
| Category | |
|---|---|
### Chart: PtOFP3
| Category | |
|---|---|
### Chart: PtOFP4
| Category | |
|---|---|

## Slide 2
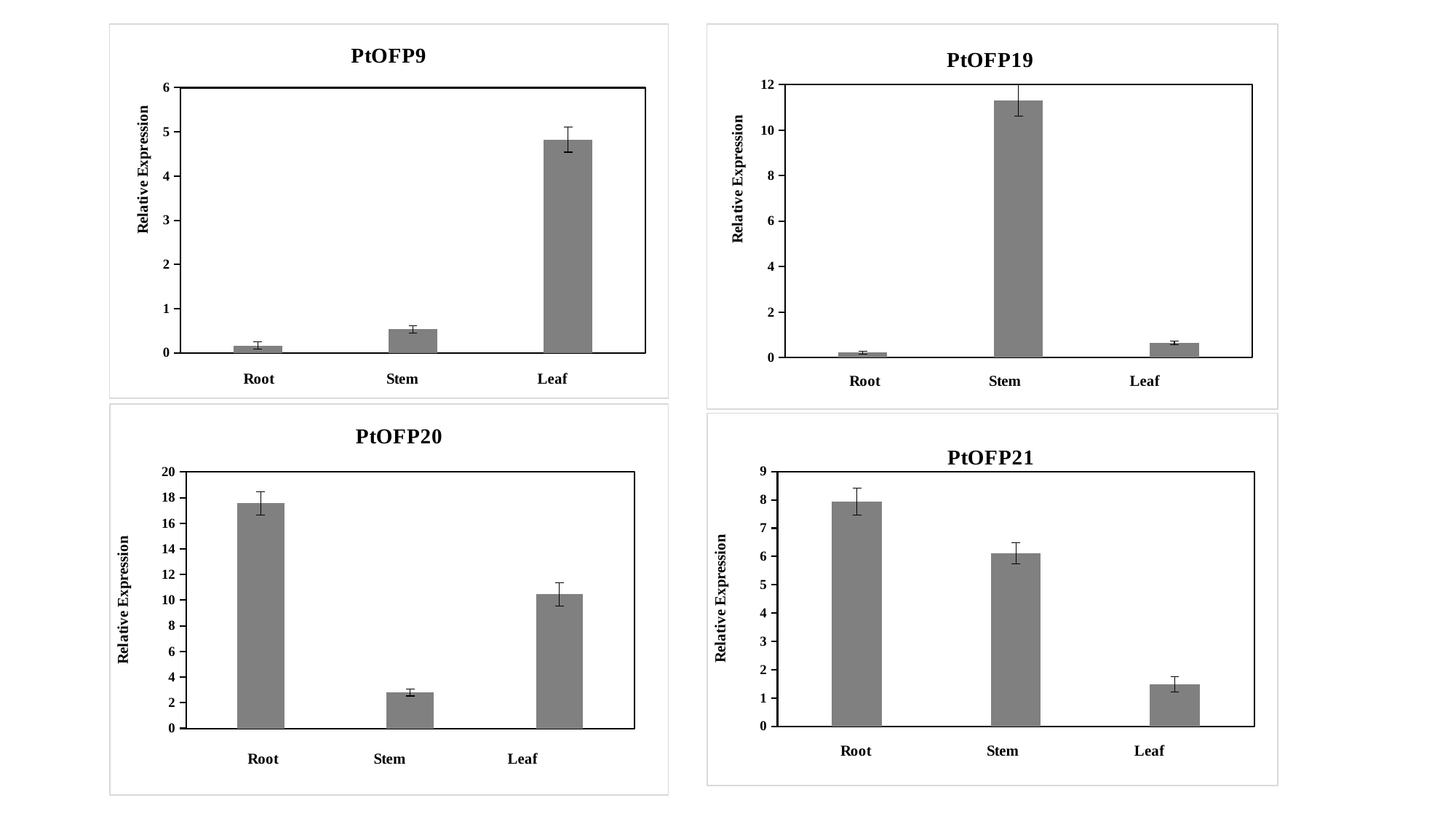

### Chart: PtOFP9
| Category | |
|---|---|
### Chart: PtOFP19
| Category | |
|---|---|
### Chart: PtOFP20
| Category | |
|---|---|
### Chart: PtOFP21
| Category | |
|---|---|

## Slide 3
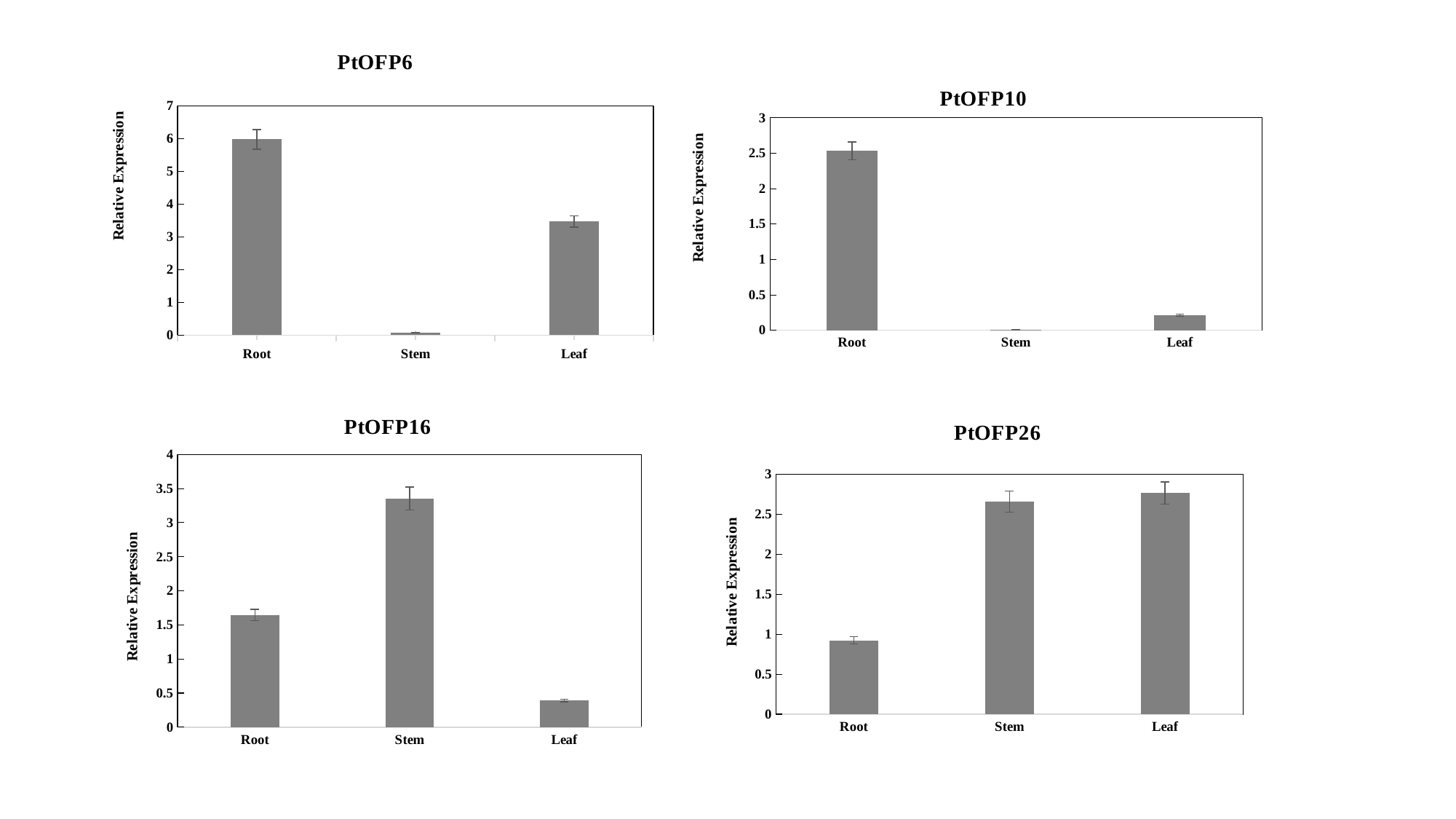

### Chart: PtOFP6
| Category | |
|---|---|
| Root | 5.974833333333334 |
| Stem | 0.07300000000000001 |
| Leaf | 3.4656666666666665 |
### Chart: PtOFP10
| Category | |
|---|---|
| Root | 2.5346666666666664 |
| Stem | 0.0065 |
| Leaf | 0.21533333333333332 |
### Chart: PtOFP16
| Category | |
|---|---|
| Root | 1.6445 |
| Stem | 3.3545 |
| Leaf | 0.39233333333333337 |
### Chart: PtOFP26
| Category | |
|---|---|
| Root | 0.9228333333333333 |
| Stem | 2.6598333333333333 |
| Leaf | 2.767333333333333 |

## Slide 4
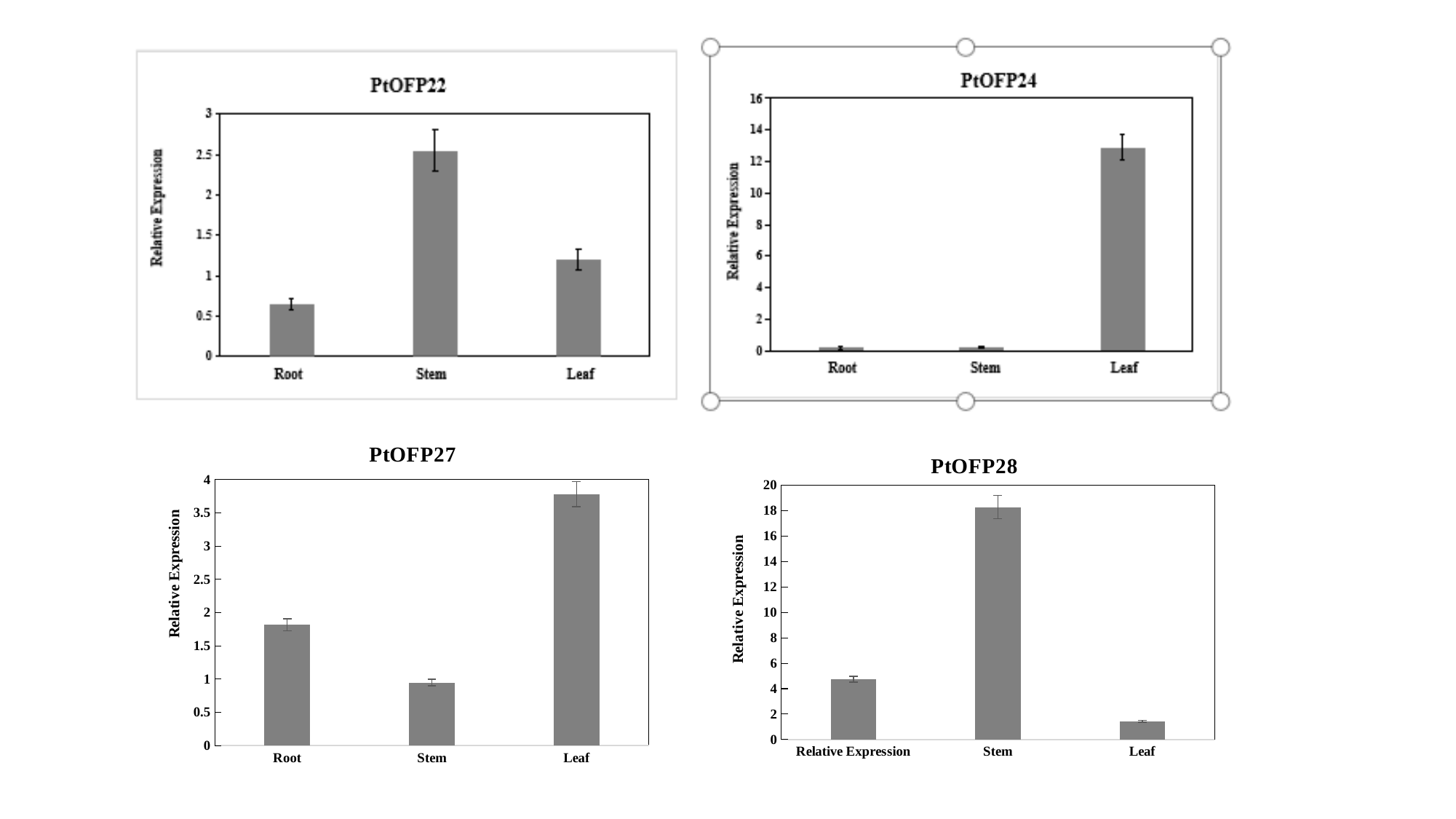

### Chart: PtOFP27
| Category | |
|---|---|
| Root | 1.8146666666666667 |
| Stem | 0.9483333333333333 |
| Leaf | 3.7811666666666666 |
### Chart: PtOFP28
| Category | |
|---|---|
| Relative Expression | 4.7445 |
| Stem | 18.278333333333336 |
| Leaf | 1.4353333333333333 |
