# Supplementary material for: Identification, Expression, and Interaction Analysis of Ovate Family Proteins in Populus trichocarpa Reveals a Role of PtOFP1 Regulating Drought Stress Response
Source: Front Plant Sci. 2021 Apr 20;12:650109. doi: 10.3389/fpls.2021.650109 (PMC8095670; doi:10.3389/fpls.2021.650109)
Supplement: Supplementary Table 2 — The genomic sequences of PtOFP genes. [file Table_2.docx]

**Table S2. The genomic sequences of *PtOFP* genes.**

>PtOFP1| Chr06:8398712..8400677 reverse

ATGGGCAACAATAGGTTCAGATTATCAGATATGATGCCTAATGCTTGGTTTTACAAGCTCAAAGAAATGGGCAAAACAAGAAACCATAACACCACCACTCATTCCATAAAGAAAAGACAAGCTACATCAGCTGCTGAAACTCAGCAGCCACCATCCAAACCAAAACATCCTCAATACAATCCTTATCCAAGAAAATCATACTACATCACTAGAGAGCTTATCTCAAGTGAACAAATACCTCACACTTCTCCAAGAAACTCAAAATCCACGTACACCAATTTTCCTGACCCCCCAAGAAGATCATCGAATCAAAGAAACAGGAGAAGAACCATCAAGGCCTCTCCTAAGCATGTCTCTGCAGGTTGTAACTGCCGTGCTACGCTCTGGACTAAATCAGATTCTCCTCCAGACTACTCAGCTTCTCTTTATGATGGTTCTCTTGACCAGGAGACAGATTTCTCTGACTCATTTCCACCAGAATTCAAGTCGGACAGTGCTCTTGCTACCGTCTCATTTGATAAAATGTTGTCTTGGTCAAGCTCTTGTGACTGCAAACTTGATTCTATTGACAATGATGGCATTGTTATCAGTGTGGATAAGAAGTCTACTGCTAGGAATTTAGATAATCCGAAAGTGTTTCATAGCATATCTGATCTTGATCTTCCTCCAATCGTAACAAAGCCTGCTAAATTTGATGATCAACTTGAAGATACCAAGAAGAAAGAAACCCAAGAACCAACCAAGTATAGAAGGAGTCCAGCTAAATACGAGGAAACAAATGCTCATGCCTCTTTATCTGTCAAGGTTGTAAAAGAAGAGAGCATTGCAGTGAAAGAATACAAGACCAGTTCTGTCCGGAGAAATTCTGTGACTTCACCAGGAGTCAGGCTAAGAGTCAATTCTCCAAGAATCTCAAATAAGAAAATCCAGGCTTATAATAATGGTCGAAAGAGTGTGTCATCGACGACAAGTTCATCGTCGCGGTCGCGAAGAAGCCTTTCGGATAGTTTGGCAGTTGTGAAATCTTCTTTTGATCCCCAGAAAGATTTCAGGGAATCGATGGTGGAGATGATAGTGGAGAACAATATCAAGGCATCAAAGGACTTAGAAGACCTTCTCGCTTGTTATCTTTCACTCAATTCTGATGAATATCATGACCTTATTATCAAGGTGTTCAAGCAAATTTGGTTTGATCTTACAGACATCAAGTTGAAgtgagatagatcttcaattgtatattacctttttctttattttttccaaataaccatgttgtcattgttatataatccaaatcgaatatcagataattaagtacatagtgtatagttttatcatgagcaattcttgagattagcataaataaattagatttaatgtagttatatcatattttgtttgctcccctaactttccaaaggggacaagaaggtaaaacgcaagattgccctgatatatcatgcatgctggctagcaaccacctcaattgactaatttccagcaaaccaacaagttattagatattactgtcccaaaatcagccttactgttcatttcctctttgattctgtgtcttttcagTAGTTGCGCGACTACATGTGAATCTACTTACAAGTTAAAGgtaagatcttggtttaagagtggcttaaggcttagctccccggagtcctactagttattccagaagaagtatcgtcatgtgtgacaataaattttcttttttcaatgcacaagaacaaatgataacactgtaaatcatagacaatataaatggatacaagaactgcaagataccctttaaggacaagattcagtctttattgtttcttagaagccctttctttttatctctctctagGCAGTATATTCAGCTTTCATAACACTCCCTTTTCCAGAGAAGAACACTAAAGAAAGAGAACAAAAGCTGCTGATTCAAAAATGTTTAAAGCTGATCAACAATGGTTAA

>PtOFP2 | Chr06:14923700..14924610 forward

ATGTTTCGAGGCTCATGTCGGACCCGAAACTTATCGGACGTGGCTGAAAAAGCTGTGTTTGTGCCTCAAAACCATAAAAACTTCCACTTGATCGACCATTTACCTCCCAAGGCTCGACCTTTCCCTTCTATTTGCATACGCAAATGCCCTGAAGCAACAAACCAAGCCATCAATCCCTCTATCATCTCCAGGAAAAACCTATCACACCGGTATCCTCCTGCTTCCCCCATTTTTCCTATGAATCCATTCTATAAAGAATTGGGTTTCCAAGAGAAAACGAAGGGACGTTGTAGCTCAATTAGAAACAGGAGTAAAAAGAAGAAGAACATCACTAACAAAAAAGACCAGATGAGTTTGTTGAGCTCATCTTCACAAGACAGTGCATGTTTTGGAGGTCGCTATTACTGGTTCAGCAGCGAAGATGAAAACAAGAGAGAGGATGATGAGTCGGACACTCTTTTCTCTTCAAGAAGTCTTTCTTCAGATTCATCCGGATCTCTCAGGCACCCTTCTTCTCGCCGCAGAAAGTACACTTCTCGGAGGAGAAGGGCAAAAGTGAAGAGTTCTCAAGTGGGTGGTTTGCCATTAGATGGAAATGTGAAGGACAGCTTTGCAGTGGTGAAGAGTTCTAGTGATCCTTATAACGATTTTAGAAAATCAATGGTTGAGATGATTGTTGAGAAGCAGATATTTGCAGCTAAGGATCTCGAACAGCTGTTGCAGTGTTACTTGTCTTTGAACTCTTATCATCATCACGGGATAATTGTTGAGGTTTTTATGGAGATTTGGGAGGCTTTGTTCTCTAATTGGACTGCTTGTTGAttttcttctatctttgttttaaaacatgtagtttagagttctaactctgataaactgtgttaaattaatagattagttttccattaatt

>PtOFP3| Chr06:22116784..22117608 reverse

ATGAAGCTCCCTTTTCTCTCCAAGATTAATACAAACACAGATCAATCAAAAAGGCCTTTATGGCCTTGGCCTACTTATTGTCACCAACCAAGAACCCTCTCTTTTAGTTTTAGAACAAGTGATGGCATGTTCAAGACCATCAACTCAGCTTTCTTAGATGCTACTAACAATGATGTGGTGGACAGCACACCTGAGTCATGGTTCACCAAATCTTGTGAATCAGCTAGCTTCTCAACAGCATCAGATGATCAGTCAGGAGCTATTGACCCTATAGAGACAGTAATCAGAGGTTTAAGGTCAGAGAGGCTGTTCTTTGAACCAGGAGAGACTAATTCAATCTTGGAAGAAGCTAAGGCAGGTGATGAGTTTCCATTTAAAGAAACTGTGGTGCTGTCAATGGAATCTCAAGACCCTTATTTGGATTTCAAGAAGTCAATGGAGGAGATGGTTGAGGCTCATGGTTTAACAGACTGGGAAGGCCTTGAAGAGCTTTTGTCTTGCTACTTGAAAGTGAATGGAGAGAGCAATCATGGGTACATCGTTAGTGCTTTTGTAGATTTGCTTGTTGGTCTTGCTTTTGCTTCTTCTTCTTCCTCCTCTTCTTCTATTACTAGTACTAGCACTACTCAACATCATCATGATTTTTGTTCTTCTTCTCATCATTCTCCTTCTTCTCCTTTGTCTTTATACACTTCTTCTACATCTGATGATGATTCTTCTTCTACTCCTTGCTGTGTATCCTCATTAGAAAATGGGGCTGATATAATTAGTCCCTGTTTAACTTCATTAGAAGCTGAAAATGGGATTAAAAATATTAATCAATAG

>PtOFP4 | Chr16:13760304..13762189 forward

ATGGAAGACATTGATTGGCATTTCGCAgtaaaaaaaatcacagaggctttgtcacatgggataacaaattgaagagcttggttcttttaataaattaaagtaataaaaaactgtgatgattataagagaccagattttatgttttgcttaggtgaattaggagtcgtggccacagaaagtcatttacacttggccccatccccatttctttgatgctacctttttctccctttatggaccacacctcccttctctccaattccaatttgtccctctagTTATCCTCTCAACACTCTCAAGGCTCAGCCAAAGCTTTTGACATAGACAATCCAACAATTTTTTCTGTTCCGCTAGCCACTATTTTCACTCACAAATACACAGAAAAAGAGGGTGAGGGGAAGGGAAGGGTGGTGTTTTTGATGGGCAACAATAGGTTTAGATTATCAGATATGATGCCCAATGCTTGGTTTTACAAGCTCAAAGACATGGGCAAAACAAGGAACCACAACACCACCACTCATTCCACAAAGAAAAAACAAGCTACATCAGCAGCAGCAGCAGCTGTAGCTGAATCTCAGCAACCACCGTCCAAGCCAAAACATCCTCACTACAATTCTTGTCCGAGAAAATCATACTACAACACCAGGGAGCTTATCTCAAGTGACCAAAAATTTCACACCTCCCCAAGAAACTCAAAATCCACAGACACCCTTTTTCCTGACCCACCAAGAAGATCATCGAAACAAAGGGCTAGGAAAAGGACCATCAAGTCCTCCTCTCCTAAGCTAGCCACCTCTTCTGTCTCTGCCGTTTGCAACTGCCGTGCTACCCTATGGACTAAACCAAATTCTCCTCCAGACTACTCAGCTTCTCTTTCTGATAGTTCTCTTGATCAGGAGACGGATTTCTCTGACTCATTTCCACCAGAATTCAGGTCTGACTGTGTTCTTGCTACTGACTCATTTGATAAAATGTTGTCTTGGTCAAGTTCTAATTGTGACTGCAAACTAGATTCTAATAACTATGATGATATTGTTATCAACATGGATGAGAAATATATTGCTAGGAGATCAGATGATGTGGACGTGTTTCACAAAATATCTGATCTTGATCTCCCTCCCATCATAACAAAGCCCCCTAAATTTGATGATCAAGTGGAAGATTTCAAGAAGAAAGACACCCTGGAACCAGTCAAGTATAGAAGGAGTTCAGCTAAATATGAGGAAACAAACGCTAATGCTTCTTTATCTGTTAAGGTTGTCAAAGAAGGGAGCATTACTGCAATGAAAGAACATAAGACTAATACTACTGTCAGGAGAAATTCTGTCACTTCACCAGGAGTTAGGCTAAGAGTCAATTCTCCAAGAATCTCAAATAGGAAAATCCAGGCATACAATAATGGTCGAAAGAGTGTGTCATCAACTACAAGTTCATTGTCTCGGTCGCGAAGAAGCCTTTCGGATAGCTTGGCAGTTGTGAAATCTTCTTTTGATCCTCAGAAAGATTTCAGGGAATCAATGATGGAGATGATAGTGGAGAACAATATCAAGGCATCAAAGGACTTAGAAGACCTTCTTGCTTGTTATCTTTCACTCAATTCTGATGAATATCATGACCTTATTATTAAGGTGTTCAAGCAAATCTGGTTTGATCTCTCTGACATCAAGTTGCAGTGAaatctttccattgtatattattacccctttttttcttttttttttaatttctccgaataaccatcttgttattatgatataatttcttatcatgagctagcagttcttcatattagcagaaataagttaattaggtccaatttataaagagctggtcttctgtaatttttaatatacttatattatagtaaatcttggagagattatcaa

>PtOFP5 | Chr16:5444575..5445689 forward

cagtcaaatgcctcatctctattcagtcacttgctcaggcgctcttcaaggaaatcccaacagacacctttcacctctccttctctcacagacgcactcttaacaatcaacaATGTCCACTAACAAGAAAAAGCACCTTTTCAACACAGTCTCGGTGAACTTAGGCTGTAGCAGCTGCAAGAAACCAAAACTTTCCAATATATTCCAGCCAAAACCAAAACTCCAAACCCCCACTTACAGAAAACACAAGAAGGATCTCTACTGCTCCTCTTCTTCGACCTCTTCAACAAAAATAACCACAAACCATTCTCGAAATGACCATGAATATCATGACACCCCTTCCACATTCTCTCCATCAATGGACACACCACCCTATTTCTTCTCTGACACGGACAACAGCGGGACGTGTTCAAGAGCCGTGCGAGGATTTGGGCGTGTTGGAGGTGAAAGCGTTGCTGTAGAGAAAGATTCCGATGACCCTTATCTGGATTTTAGACACTCAATGTTGCAAATGATACTGGAGAAGCAGATTTACTCAAAGGATGATCTTAGACAGCTCCTTGATTGTTTCTTGCAGCTAAATTCGCCCTACTATCATGGGATTATCGTTAGAGCTTTCACTGAGATCTGGAATGGAGTCTTCTCTGTGAGGTCTAATACCACTACAGGTTCCGAGAAGCAGCTGCATTATTACTATGGCTGCTAGTCACGTGACTTGTGAAAATGGTTCTTGCTGTTCTGCACGTGAACGTCTTGTCGGGGTACAAAATCATTCTAAGGATGATCTTAGACAGCTCCTTGATTGTTTCTTGCAGCTAAATTCGCCCTACTATCATGGGATTATCgttagagctttcactgagatctggaatggagtctcctctgtgaggtctaataccactacagGTTCCGAGAAGCAGCTGCATTATTACTATGGCTGCTAGtcacgtgacttgtgaaaatggttcttgctgttctgcacgtgaacgtcttgtcggggtacaaaatcattcgtttgtagaatgaacggccgcataataaagtggaagtcttttttccttgttgacatgaaattttatgttttagattaggcattttgtggatgtaatggcaaa

>PtOFP6 | Chr16:5466667..5467504 reverse

gcaaaaaccaagaacaaacgagttggtgggcaagaaaATGAAGCTCCCTTTTCTCTCCAAGAATAATGCAAATACGGATCACTCATCAAGAGCTTTATGGCCTTGGCCTGCCTATTGTCAGCAACCAAGAACCCTCTCTTTTAGTTTTAGAACAAGTGGTGGAATGCTCAAGACCATTAACCCAGGTTTCTTAGATGCTACTAACACTGATGTGGTGGACAGTACTTCACCTGAGTCATGGTTCACCAACTCCTGTGAGTCAGCTAGCTTCTCAACAGCATCAGATGATCAGTCAGGAGCTGGTGAGTCTATAGAGACAGTGATCAAAGGCTTGAGGTCAGAGAGGCTGTTCTTTAAACCAGGGGAGACAAACTCAATCTTGGAAGAAGCTAAGGCAGGTGGTGAGTTTCCATTTAAAGAAAGTGTGGTATTGTCAATGGATTCTCGAGACCCTTATTTGGATTTCAAGAAGTCAATGGAGGAGATGGTTGAGGCTCATGGATTAACAGACTGGGAAGGTCTTGAAGAGCTTTTGTCTTGCTACTTGAAGGTGAATGGAAAGAGCAATCATGGGTACATCATTGGTGCTTTTGTAGATTTGCTTGTTGGTCTTGCTATTGCTTCTTCTTCTTCTTCTTCAAGTACTATCACTACTGCTAGTACTACGCAACATCATCATGATTCTCCTTCTTCTCCTTTGTCATTTTACACTTCTTCTGCATCTTCTGATGATTCGTCTTCTACTCCTTGTTGTGTATCCTCGTTAGGAAATGAAGTTGATATAATCAGTCCTTGTTTAACTTCATTAGAAGCTGAAAATGAGATTAAAAAGTATTAA

>PtOFP7 | Chr13:15811104..15812812 reverse

aaactttagccaatgttttcaggccctcagtttcttttagtaaagttgaattttggagatcattatgcataacaaatcctgattatgtttgtctaatccaaaaaaaaaaaattctgatttgtaattctgttagttgttatgatgtaagaggaacacacctgctttcATGCTGAACTTTTTTCTAGTGAGCCCCCATCTATTCTTTACCTTTTATAACCCAAACTTTGTAAGCTCTTTTTATCTTTTCAGTAATCAAAGAGAGGGAAAGGTAAACCAACTCAGACAAATCTCAGAGAGGGAAAGAAAAATGGGTAATTACAGGTTTAGATTATCAGATATGATACCAAACGCCTGGTTTTACAAGCTCAAGGACATGAGCAAAAGCAGAAAGCATTACACCTCTCGAGCTTCCAAGAAAAAACCACCTCCAGGAACTGTAACATCGCAAAAACCCAACATTTCTCATCAAAGATACTCTTATTACTTCACCACAGAACCTGAAAGAGCTGAGAAATTAGATTACTATTCACCTGCAAATCCAAAAGCCTCAGATACTCACTTCCCTGATCCACCAAGAAAATCATCCAATAGAAGAAACAAAAGAAAAACCATTTACAAGCCTTCTCCAAAACTGGTGTCCACATTCTCTGCTGATTGTAGCTGTCGCGTTACAGTTAACTCGAACCTGACCGGGTTTATTCCAGGTGATTCACCTGACTGCTCTAGCTCTCCTGTTGAGAGCTCTTATGATGAGCTTGACTTCCTATCAGAATCTGATGAAGATGATGGTTTTCTTGTTCCTGATTCAATTGATCATCATTTAGCCTCCTGGTCTAGCTCTTGTAACTGTAATGTTAGCTCTTCAACCACTGACATTATCATTGATATGAATGAAGAATCCTATGAAAGGAAAATCAAGGAGGTTGAAGGGTTTGGTAGGATCCCTGAGCTAAACCTTCCTCCTATACTGACCAAGCCTGAGAAATTCAACGACAATGAAGTTACTAAGTTCAGAAGAAGTTCATCCAAATTGGAGGAGGTCAAAGCACATAGGTCTCTCTCTGTCAAGATTGTCAAGGAGAAAAGCATTAGGACTTATAAAGAGAAAAGGATGAATCCTCCTACAAGAAAGTCCTCGGTGAATTCGGCAAAAGGGATAAAACTCCGAGCAAATACTCCAAGAATTGCAAGCAGGAAAATTCAGGGTTGTTCTCGAAAAAGTGTGTCATTGTCAAGAAACAAGACCCTTTCAGAGAGCTTTGCAGTGGTTAAGTTCTCAGTTGATCCACAGAGAGACTTCAAGGATTCAATGGTGGAGATGATTGTGGAGAACAATATCCGAGGTTCAAAAGATTTGGAAGACCTTCTTGCTTGTTACCTTTCTCTCAATTCAAAAGAATACCATTATATCATTGTCAAGGCATTTGAGCAAATCTGGTTTGACATGACTGACCTTCACTTGTAAttgtaaagccttgttagattatcagaagcatgaataatatgatcctgtaaatgctagtgctttaattaaaatttctattctctgcttagaattgttggtttaatcatggagaattgcttcaatatgtagttgatttttaaagaatttttaccaggaaaatgtacctaaagaatctttgattccttcccaattcacagttactaagcccaagcaaaaccatcaagttcatgactgacaacaaaac

>PtOFP8 | Chr19:15487343..15488677 forward

catctttttccaggtatcaaagagagggaaaggtgtaaacgatctcagagagggaaagaaATGGGCAATTACAGGTTTAGATTGTCAGATATGATACCAAATGCCTGGTTTTACAAGCTCAAGGACATGAGCAAAGGCAGAAAGCAATACACCTCTCAAGCTTTCAAGAAAAAACCACCCCCAGGAAATGTAACATCACAAAAACCCAACATTTCTCATCAAAGATACTCTTATTGCTTCACCACAGAACCTGGAAGAGCTGAGAAATTCCATTTCAATTCACCTGTAAATTCAAAAGCCTCAGATACTCACTTTCCTGATCTACCAAGAAAATCATCCAATAAAAGAAACAAAAGAAAAACCATTTACAAGCCTTCTCCAAAACTGGTCTCCACATTCTCTGCTGATTGTAGCTGTCGCGTAACGGTTAACTCAAACCTGACCAAGTCAATTCCAGGTGATTCACCTGACTACTCTAGCTCTCCTGCTGAGAGCTCCTATGATGAGCTTGACTTCTTATCAGAATCTGATGAAGATGACGGTTTTCTTGTTCCTGATTCAATTGATCATCAATTATCATCCTGGTCTAGCTCTTGTAACTGTAATGTTAGCTCTTCAACCGCTGACATTATCATTGACATGAATGAAGAATCCTATGAAAGGAAAATCAAGGAGGTTGAAGGGTTTGGCAGGATCCCCGAGCTAGAGCTTCCTCGAATATTGACCAAGCCAGCAAAATTTAACGACAAGGAAACTGAAGTTACTCAGTTCAGAAGAAGTTCATCTAAATTGGAAGGGGTAAAAGCACATAGGTCTCTCTCTGTCAAGATTGTCAATGAGAGAAGCATTAGGACTCGTAAAGAGCAAAAGAACAATCCTCCTACAAGAAAATCCTCAGCGAATTCGACAGGGATAAAACTCCGAGCTAATACTCCAAGAATTGCAAGCAGGAAAATCCAGACTTGTGCTCGAAAAGGTGTGTCATTTTCAAGAAACAAGACGCTTTCAGAGAGCTTCGCAGTGGTTATGTCCTCAGTTGATCCACAGAGAGACTTCAAGGATTCAATGGTGGAGATGATTGTGGAGAACAATATCCAGGATTCAAAGGATTTGGAAGAACTTCTTGCTTGTTACCTTTCACTCAATTCAAAAAAATATCATGATTTCATTATCAAGGCATTTGAGCAAATCTGGTTTGACATGACTGACCTTCACTTGTAAttgtaaaatcttgttagattatcagatgcatgaataatatgatcttgtaaatgttacaacttgaatgaaaatttctatcttttcttacaatcattggcttcccaagttgaaata

>PtOFP9 | Chr04:5132470..5134525 forward

aagaaatctccaaatcactaaagccataaagagagaactgtcagtcctaattgtccaaaatctgcccccatttcaaagccctggtagctggttcataccaaaatacaaaagttcacgagctactgatgcaactacactggctatgtggacagaattatacaccataattcttgtgaatgaactttgggtgatcctgcacctagaggatcaaagggcttcccgtctgagcatgggtttaggcatgaattcatttccaagtacaaatcataaagtgaaaaaaagaagccaaagtggtcacaagattaagggcgggtgcctcttttgaaaaaaaaaaaaaactagcagcagaaaacaaagtaagaaacccaagtccctatttataagcctctaactgtcccaaactgctacagctctctctctccccctcccattaacaccccattcagtttcttatcatcatctccatcttcttcttcttctcttggcaactggataaccttaaaaaaaatcaagctattctcatccaaacaaaagaaagaaaactactccataacaaacaggaaaaaccctgtttgtcagaagtagaaaaggATGAAGTGGGGTAGGAAAAAAACCCCATCTTCTTCGCGCCCTTCTTTAATATCTCATGTTTTCCCCACTTCCTGGCTAACAAAGTTCAAGCACATGAGCATCAACCCAGGGCAAGAACATGCAAAAGCGAAGCAAAAAGGGAAATGGAATTCTGTTTCCGCAAGTCCATTGCCATTTGCTCGTGGTGAAGGAGGAGGTAGATTCTATGGAGGGGATGGCGATGCTTTTTGGAGACTTTCTTTTGGTGACGAGAGTGCTAGCACAGGTGCTTTGAGTTCATTTCATAATGATTTGGACAGTGAGCTCCAGGCTCCACCATCAAGTTGTCACAGCTGTAGATCAAACGCTACAAGGGTAAATAATAGAAAAGAAGACAAGATCAGGTTCAGCAACAAGGTTTCTGAAGCAAGGAAAATGAGAGGACTGCCAAGAGAAATTGAGATTTTGCCTGAAATGGATGCATGTATAAGTGAAAAGGTAGCAGAAATCAGGACCCCAAGGTTGAGGGTTGGGAGAGAGGAGAAATTGAGGAAAACAGATCAAAGGGTTTTCGAAGCGCAGCAGTTCAATTTGGATGGAGAATCGTATGAAGCAGAGAGGGTATCAAGAAAAGAGACATCAAAGAATATTTCTGAAACGGAATCAGAAAGAACGATTGGGAGGATAGAGAGAGAGGACTGTAAGTTGACAGCTTCTCATTCAAAAAAAGATTTCTCCACTCATCTAAGAAAGACTAAAAAAGACTTTGTATTTGCTGCTCAGAATGAAAGTGATGGATTTTCTGCTGAAAATTTGAGTTCTGAGTGGCAAACGTTGAAAGACATGAAGATTGAAGAGCTCAAGACAAAGAGGGAGAAACAGAGAAAATCTCTGTACATAAACAGAGAATTGCAGAGGAAAAAGAAAAGTAAAGTCAGGGCTATTTCTCCAAGAACAGCTTCAAAAGTCGAAATCTGCAGAATAAAAGCACTTGAAGACATGAAGAAAGCCAAGATGAAAAAGAAGAAAAAGGCTAGGGAGAAAAAAATGGAGGGATTCACAGGCCTAGAGAATTTCGCTGTGGTGAAAACCTCGTCTGATCCACAGAAGGACTTCAGAGATTCAATGATTGAAATGATTGAGGAGAAGAGAATTAGCCGGTCAGAAGAGCTCGAAGAACTCTTGGCGTGCTATCTGACATTGAACGCTGACGAGTATCATGATCTTATTGTCAAGGTATTCCGGCAGGTATGGTTTGATCTGAACGAGGCCTGCTCTGATACTGAACTAGAGAATGAACAAGGTTACGATGAATAAccgcattagtcagttaattatagcaagaaacttgtaaatttcctgtaaattgctaagatattactgttataacatgatgatgcctctaattaattatcattccatgaaaaattatcatcataacttcaatgcagtgacaatttcaagaattagtgctattaaagtttgt

>PtOFP10 | Chr04:245691..246161 reverse

ATGAAAGAATCCAGGGTAGACAAGAAGCAAAAACTTCAGCAAAGAGGATGCAAGGCCTTTTGTTGCAGTTGCAGGCTAAGTGTCTCTTCTTCTGAGGAAGCTGAGAGCTCGAATCCCGATCGGTTTGCATCGATCTCAAGCCTGGCACATGCGATGGTACAAGAGAGACTAGACCAGATGATTAGAGAAAGGCAAGAGGCAAGACAAAGAGAGAGAAGAAGGAGACTAAGAAGTGATGGGACCAAGTTTATAGTAATGGTAGCTATGGAGAAAAGCTCTTACGATCCAAGAGAAGATTTTAGAGAGTCCATGGTTGAGATGATCATGGCAAACCGGCTACAAGAGCCAAAAGATCTCCGTAGTCTGTTGAATTATTACATGTCAATGAACTCTGAAGAGTATCATGGAATGATACTGGAAGTTTTCCATGAGGTTTGCACCAATCTGTTTTTATGCTGTAAATGCCATTGA

>PtOFP11 | Chr04:21223914..21224683 reverse

ATGTCTTCAAAGAAGAAAAATCTCCTTCAATCCATACTCACACCCAATGCAGGCAGTGGCTGTGGCTGTGGCAGGCCAAAACTCTCAGATGTATACGAGCCAGCACCAAAACCTAAACCCAAAACCTCAATTTCCAAAAAAGATCCGAACCCTAAACATTGTTCCTCTACAATAACCTCATGTGATAAGAGTGTAGGCTTCTCATTACCGGACAGTGAAGAAGAAGGCTCCACTTCCACTACTTTCACTTTGAAAAAAGACAACAACACGTCATCAACCCAAAACTCCGAATCCGAAACCTATCCAAAAGCATCCAAAATCACTGACAGCATTGCTGTGGTGAAGGATTCTGATGACCCATTTCAAGATTTCAAGAATTCTATGTCGCAAATGATCTTGGAGAAAAACATCTACTCTAAAGATGATCTTGAAGAGCTTCTTAACTTCTTCTTGGAGCTGAATTCTCCTTGCCAACATGATGTTATCGTTCAAGCTTTCACTGAGATCTGGAAAGAGATCAGATGTTTGTCACATGAATCCTAGctgtgcatggaacttgtatgttggatagttttgatgttaatttgcttacaatacttcggcagtgagattttattttggaaaaattatttattttcttgacggtaacattttacttaatttttccttacgtttattgttgtgattgtgtataaatgtacttacagaata

>PtOFP12 | Chr18:10790893..10791900 reverse

ATGGAAAACCGATTTAAGACGCGAATCTCTCGCATGTTTCGTGGCTCATGTCGGACCCGAAACTTATCGGACGTGATTGAAAATGCTGTGTTTGTGCCTCAAACCCATAAGAATTTCCACATGATCGAGCCTTTGCCGCCCAAGGTTCGACCTTTCCCTTCTATTTGCAGACACAAATGCCCTGAAGCAACAAACCAAGTCCTCAATCACTCTATCATCTCCAGGCAAAAATTATCACACCGTTATCCTCCTCTTATAACTGCCAATACTAGTGGACACAGTTCTTGCCCTCCTGCTTACCCCATTTTTCCTTTGAATCCATTCTACAAAGACTTGAGTTTCAAAGAGAAAAAGAAGAGTTGTCGTTCAGTTAAAAACAGAAGCAAAAAGAAGAATATCATTAGCAAGAAAGAACAGACGAGTTTGTTCAGATCATCTTCACAAGATAGTACATATTTTGGAGGTAGCTATTATTGGTTTAGCAGCGAAGATGAGGACAAGAGAGGGGATGAGTCGGACACTCTTTTCTCTTCAAGAAGTCTTTCTTCGGATTCATCAGGATCTCTTAGCCACCCTTCTCATGGAAAAAAGTTCACTTCTCGGAGGAGAAGGGCCAAAGTGAAGAGTTCTCATGTGGGTGTTTTGCCATTAGATGGCAAAGTTAAGGACAGCTTTGCAGTGGTGAAGAGTTCTAGTGATCCTTATAATGATTTTAGAACATCAATGGTTGAGATGATTGTTGAAAAGCAGATATTTGCGGCTAAGGATCTTGAACAGCTTCTGCAGTGTTTCTTGTCTTTGAACTCTTATCATCATCATAGGATTATTGTTGAGGTTTTCATGGAGATTTGGGAGGTTTTGTTCTGTAATTGGTCTTGAttatgagcagaaaataaaactgtactgcttgttagctttctgctatctttgttttaaagtatgtagtttagagttctaactctgataaactatgttaattaatagattttctttctctgttattttctc

>PtOFP13 | Chr02:25046408..25047442 forward

ATGCCTAATCGTCTCAAGCATAAGCTCTCTCGTGTTATCACCCCATTCCAACTCTGTAGATCCAAAGATCCTTCTTGTCCCGAAGCTCCAATTCCAGCTATCAACAGGCTCTCTCCTTTCAACCCCAAAGCACTTGACATCAATTACCCTTGTAACCTCCAAGCACCACCACCGCCATCAACACCTTATTACAAATGCCGAGTGTCAAGGAAGACCATTTCCGTCGGCTGCAAATGCCAATCGCGATCGTGCCCGCGCTGCTGCATGTCAGATTGGAGCATTGAATCGCCCGATTTTGCGGGCAAGAAAGAAGCCAGGTGGCAAGCCAAACCTCACCTAAACGTGCCCTTCTCATTTTCTGATGGGAGTGGGGACATGTCACCCTTTATGGTAACTGGAAAAAACAAAAACAGAGAAATTAATATCAAGAAAAACAAGGTCAAGACAGGTGTCTTGTCTGTTGATACTAGTGGGTGCTTTAGCAGTACTGATGTTGCTGGTGAAGAAAATGAAACCCTACTTTGTTCTTCAAGAAGTTTCTCCTATGATTCTTCTTGTGAATTCAGCCATTCATTGGATACTATAGCCAGGCAATCAGAATATCATGAAGCGTTTAATAAGCCTATAGGGAACAAGAAAGTGAGTAACCTAAAGAAAATTAAAAAGCTTGGACACCAAATTTCATTGAACAAGTGGAAAAGATCAAAGACTGTGACGTCTCCAGAGATTCCTTCACCTGTGAGATCATCCGTTTTGAAGCGGGTGATATCACGCAAGGTTGATGGGAGAGTGAAGGAGAGCGTGGCAGTGGTGAAGAAATCACAGAACCCACACCGGGACTTTAAGAGGTCTATGTTGGAGATGATATTAGAAAAGCAAATATTTGAGGCCGAGGATTTACAGGAACTTTTGCAGTGTTTTTTGTCCTTGAATTCGAGACAGTATCATGGAGTTATCGTGCAGGCATTCTCGGAGGTTTGGGAGATCGTTTTCTGTGATTCTCCTGTAAATAAGAGAGCTTCTATCAGAAATTAA

>PtOFP14 | Chr07:2103608..2105090 forward

ttttttccaaaaaaggattactaattacttttccaatttatcgtggtcaatatatattaatgttagtgtagacggctgttatgcacattttcaccctcttctttcctaatcattgaaaaaagaaaaaaaaaggaaaaagaaaattcatcccacccctatataaacctttcagcttcctcatacaaacagtttaatgctttgacacttacaaagcacttgagcccaaaagccgaaaacattaacaATGGCTAAACGTTTCAGGCTCAAATTCTCTCGAGTGATCTCCTTTCAATCTTGCCGTTCCAAAGACCCTTCTACTCTCCCATCAAATCCTGTCCCTTCATTTCTTAGACTATCTCCAGTCAACCACAACTCCATCATCATCAACAACCTCCATCTCCCACCCTCACAACCACCTCCTTCCAAACCTCTCCATCACTCTTCCATCAGGCGACATGTGTCCTCGGCATTCACATCAATGGGCTGTGGGTTTAGATCAAAACCCTCCACACATTACCTCTCTGAAACCGACCACACCAAATCCTCTCCACCAACTGAAAATTTCCACTGGGAAGAGGAAGAAAAATATCACGTTGTGGCCAAGCTCTTTGACGACGATTCAACACCCCGCCGCAAGATTTACAACTCTTCAGCCTCTGAGGACTCCAAAAACCACGACGTTTTTCTCCCTCCATCTAAAATCGAGAAGAAAAAACGACGAGTCAAAAAGAAGAAAACGGCATCAAGAATCCGCATCAGCACTTCCTCAGCTGATAGCGGGATATTTTTTAGCGGCGACGAGCATGTCATAAACGACGAAGAAACAGAAACTCTAGTCTCTTCTTCGAGGAGCTTCTCCACTGATTCTTCCTCGGAATTCAATCCCCACTTAGAAACAATACGTGAATCTCCATTTTCACGTAAAAAGAGAGCTAAGAAGGCTAAAGGGCGTTGTGTTTTAAAGAATGGAGCGAAAGGAACAACGAGAAGAGGACGAAAGGAAAGAAATAGCCGCGATGGTTCTTTGTCGCCTGCAAGATTGTCGAGGTTTCAGTGGCTGATACCGTGCACGGTGGAGGGGAAGGTAAGAGAGAGCTTTGCGGTGGTGAAGAGATCAGAGGACCCATATGAGGATTTTAAGAGATCAATGATGGAAATGATACTAGAAAAGGAAATGTTTGAAGAGAAAGATTTAGAGCAGTTGTTGCATTGTTTCTTGTCTTTGAATTCAAGGGAGCATCATGGGGTTATTGTTCAAGCTTTTAGTGAGATTTGGGAGACTTTGTTTTGTAGAAGAAGATCTATTTCTTATAGGGTTTCAGCTGTTTAAtttcttgttttttttttgttagagctctttgaatgtgtagcggtaaattaagggtaataacaataaaaattagtactataatcagaaagattgctcgtgttaatctaccgtgacactggattatcctcctttttcttaatagtatgtactagc

>PtOFP15 | Chr05:9772353..9773429 forward

ATGGCTAAACGTTTCAAGTTCAGATTCTCTCGATTGATCTCCTTTCAATCTTGCCGTTCCAAAGACCCCTCCCCTCTCCCATCAAATCCTGTTCCTTCATTTCTTAGACTCTCTCCGGTCAACCACAACTCCATCATTATCAACAACCTCCACCTCCCACCATCACAACCGCCTTCTTCCAAACCCCATCAACACTCCTCCATCAAGCGCCATGTGTCCTCAGCTTTCACATCAATGGGCTGTGGATTTAGATCAAAATCCTCCACGCATTCCCTCTCTGAAACCGACCACGCCAAATCCTCACAACAAACTGAAAATTTCCACTGGGCAGAGGAAGAAAAATATCACCTTGTGGCCAAACTCTTTGATGACGACTCAACTCCCCGCCGCAAACTTTACAACTCTTCGGCCTCTGAGGACTCTAAAAACCACGACGTTTTTCTCCCTCCGACTAACATAGAGAGGAAAAAACGACGTGTCAAAAAGAAGAAAAGGGCTTCAAGAATCCGCATTAGCACTTCTTCAGCTGATAGCGGGTTATTTTTTACCGGCGACGAGAATGTCATAAACGTTGAAGAAACGGAAACTTTAGTTTCTTATTCAAGGAGCTTCTCCACTGATTCTCCCTCAGAATTCAATCCCCACTTGGAAACAATACGTGAATCTCCATTTACACGTAAAAAGAGAGGTAGGAAGGCGAAAGGAGGTGTTTTAAAGAAAGGAACAACAAGAAGAGGACGAAAGGCAAGAAATAGCTGCGATGGTTCGTTGTCGCCTGCAAGACTGTCGAGGCTTCAGTGGCTGATACCGTGCACGGTGGAGGGGAAGGTAAGAGAGAGCTTTGCAGTGGTGAAGAAATCAGAGGACCCATTTGAGGATTTTAAGAGGTCAATGATGGAAATGATATTAGAGAAGGAAATGTTTGAAGAGAAAGATTTGGAGCAGTTGCTGCACTGTTTCCTTTCTTTGAATTTAAGGGAGCATCATGGGGTTATTGTTCAGGCTTTTAGTGAGATTTGGGATACTTTGTTTTGTAGAAGAAGAAGATCCATCTCTTCCAGGGTTTCAGCTGCTTAA

>PtOFP16 | Chr05:22517159..22518862 reverse

ctgctcaaccccccattttgttatcgtttcttttcctttgattcaccttcacactcctctctttctttcctgcacaatcacagaaacaaaataATGGCAGGCACAATAGGAAGAAACCTCAATCTATGCTTCACTAAGATCAGACGTCCACTACCACCCCATGATCAATCCCCCACTACCCTACTAACCCCAGATGATCACAGCCATACATTCCTCATAAAAAACTATAATTCCCTCTATGACCCCACCATTGATTCCGCCTCCTCCTCCACTTCTTCCAGCTCCTCCTCCTCCTCTGAACCTGACTTCGCTACCGTCTGCGCCTCTCAGCGCTTCTTCTTCTCCTCCCCTGGCCGCTCCAACTCCATTATTGAATCCACACCGTCCATCGTCACTTCCTCAGACTCATCAGACAATCTAGTAGCCCCGCAATCTGACAGCAATGGTCTGACAACGAATCCCTCCAATGACAAGTCTTTGTTGGTTGATAGTTGTAATAATAGTACTCATCCCCAGTTATTAAAATCCCCAACCGTTAAAGACAGTGTTGCTGTCCCCACCTACTCACCGGACCCGTACATGGACTTCCGGCGATCCATGCAAGAGATGGTGGAGGCACGTGACTTGGTGGACGTCAACGCTAATTGGGAGTATTTGCACGAGCTACTATCGTGTTATCTTGATCTTAATCCTAAGAGTAGCCACAAGTTCATTGTTGGAGCTTTTGCTGATCTTCTTGTTAGTCTTTTGTCATCACAAATGCCAGAAGATGCTGGCCGCCGGGGAGAGGATTTTTCCTCCGGTAGCTGTGGGATTTCGCGGCAGTGCATGTAAtgtagtatgtggtttctgtcggaaaatgattgtctaatcttgttacctttaatttagcaatatatatgtgtgtgggggaacaaaattgagcctccttttgtcttctattataacaccttatcccctcaccaccatcttgcttttggttcttgtatttattaagctggaaacttttttttcgatttgttagtattgttttcgggttctagattagaaaatttgtttttttcccccgaaattgggttttttttggaatgcaggcagtgcatgattacaagtaaaggcgaggtacttgtcctaccttttctttttactccatctctctctctctctttcattttttttaatggtaattgcaagtgctcgtgaactagatgtctataattcactctttgatagttggttggggatagaagctttcaattaatccgagggacttggagggcttgttctatcaaatgtaaatttgatgagattacgtttttcactctatagtgaagctttgtttttgttggatgacgaattgatatccatcacctgttacataaattctaaaaatttcgttagacccgttttcttcctttccttttccgtactttcttctctactgaaacaaaaaagttagctgcaacgtggaagaaaagctgtaacctttggattaattttgaagacttcacgtgtaagattgaaattagttttgttaaacctttcttgcatgcttatcccactttctttttcagctaaaacttgctttaatcttccaaattaactctaataaaccacgttcttgcacagacaattaatattatgtccactcattgttttgcagaggtaatcaatcaagacaaggggactattaggttgggaacg

>PtOFP17 | Chr09:12444391..12446657 reverse

ATGgtactggatgcatacatctttttttttttaaaaaaaaattaaataatgtttaagataaacttgaatacaaaacattgcacctagctgtatatatatggagttccttcatgtcatcaattagtcccaaaattaatatatggtatggtttaaattgattccaagttcgttgatcgatctagcaggcgtagttcatacttgacctagctagctataattaaagtcgagtcacaagaatcctgttaggctcctgatcaatgacctttcacatccaattaattagctatatcttgagcgtggaggttatatccccatttccgtgatagttgtctcaaaggggtacgtagctaggttttatctatatatcaacacgaaggagatcaatacaataatgtcgatcttgctagctacaagtctaaaaacacccaagaagtaaaaagggaatttgtccaagttgcagcatcaaattacctgtaatcattccaaattaattacaatatataattttatatatggaagtcatcgtcattatgaattgagaacgccggtgagcaaatctcatggcttgattgctagttgctaatcccaattcaaagccctcaaatagttttctattcatgtcacatggaacggaaatttcgtgtgggggaatcctgggaccattcatcaatagttaatttatcacctaatatgattttactataggcattgtacatagatttgatttggttaacccatctaaaaatgccaaggaagctctaactatagtgcttttgacttctccaaacatgttacgatgataagtagtatgagtctataagaacttggtgtcttgttgatctaagatttaattcatgtctgactttaaattaaactaaataaaaattaatctcgttaaaattgataattaatttgataagttaatctataaagtcaaaatttagtttgatttaaaaaaaataatcaaaatcaaacatctaatttaataatttgattatatatatatgctagcatgtaatttgattcaattatagtttaattagcatgagaaaatagaaaccatttaaaaaaaatgcttctagaaaggcattttagaaaatgccaaagtcccttcacaaaggctcattacctctctctcgcccctaataagttaagttaaatgaactaataggaataaaagccttcctctctcaacaacactaaagaaaacgactctatctatctaaaaacaacttctcccccccctctctctagtccccaaagGCCTTAATTAGAGTTAATTCTGAAAGGGACTTTTATCAAGATCGATCACCTCCTAGCTCCAGCCTCGATCCTAGCAGTATATTAAACCTTGCAAAACTCTATTTTTTCTGTTTTTTCTTCTCTTCAATGTTTTCAAAGAAGAAAAAGACCCTTCAAACCATACTCGCATCCAATGCAGGCTGTGGCTGTGGCAGGCCAAAACTCTCAGATGTATACGAGCCGATACCAAAACCTAGACCTAGACCCTGCAGAACCTCAATTTCCCAAAAAGATCCAAACCCTAATTGTTCATCTTCAAGCTCCTGTGACAAAAGTGTAGGCTTCTCATTGATGGACAATGAAGAAGAAGATTACACCTCAACCACTATCACTCTGAACAAAGACAACACTTCATCATCACAAAACTCCGAATCTGAAACCGATCCAAAAGCATCCAAGATTATTGACAGCATTGCTGTGGTGAAATATTCTAACGACCCATTTCAAGACTTCAAGCATTCCATGTTGCAAATGGTCGTGGAGAAAAATATCTACTCAAGAAATGATCTTGAAGAACTCCTCAACTGCTTCTTGGAGCTGAATTCTCCTTGCCACCATAGCGTCATCGTTCAAGCTTTCACTGAGATCTGGAATGAGATCATCTCGAAGAGGATCGTCAAGAAACCCTGTGCTCAGTTCATGTGAgtcaaaaaatgaaagaatcctagctgtgcatggaaattgctcgcgtgaaatccacgtgaaggcattgaattcacatggggaaactttgattagtgttcggtagggtttggtcaagatcagggggcgcaacagggctttctagtggagaaaagatggatatgagattcttagattataatatttaataaagaaagaagcccaaaccttagagatgccagagatgaaccttcaaggttgtttcgattttattttgtttaatgctttggcagctggtgagtttttatttttcttgcaactgtgtacaaattatgtaaataata

>PtOFP18 | Chr09:12442016..12442681 forward

ATGAAACTCCCTTCAATCTTTAAGAAACAAGAAACCAATTTTGTTTCATGGAAATGGCCACCGTTCAGTATTGGGACATTTCCATTCCAATCTAAGGTGAATGCTTTAAAAAATTTCTACTTTGCTTTACGTGATGCAGCCAAGTTCATGACAATGCTGAATTCCTCATGGTCACAATCAAAGACTATCTCCTTGACGCCCAAGGAATCTAAAGAGGACTCGCTGGAGATTGCTGTTAATAAGGCAGTGAGATCAGAGAGGTTGTTCTTCGAGCCAGGTAACACGAGTTCAATACTAGATGATCATAACGATGAAGCTAGCAAATTCCCATTCCCTGAATGTGTAGCACTAGCTATGGAATCTGAAGATCCTTATGAGGATTTTCGCAGTTCAATGGAGGAAACTGTTGAGACTTGCGGATTAAAGAATTGGGAGGATGTAGAGGAGTTATTGGCATGGTATTTGAGAATGAATAGGCAGCAGCATCATTGCTTTATAATTGAGGCCTTCGTTGATCTATTTTCTGCTGCCCCACCGTCTTTTTTTTCTTGCCCTGTTTCTCACAGTGACTCTGCTTCATCTTCAAAATCAAAAGATTTGTGGATGATTGAAGCTAAGAGGTCACAGCCAGCTATGGAAAAGGGGAAAAGTCTAAAGAATTGCTGA

>PtOFP19 | Chr08:10416095..10416730 reverse

ATGCCCCCCATCTTCTGGAAGAACATTCTCAAATGCCTACCCACCATAATCCCATCCTCACATCCATTGCCCTCAGATCAGTTGCAGGAGTATAGGGATCCATTACCATCATCCACTACCCTCATCTCCCCCACCACATCAATTATTATCCAGAACTTCAACTCCCCCTACGACCTCTCCTCAGCACCCACCTCCAAATCCCTCAGTACTCCCTCCACCAACTCCTTCTCCTCCTCTTACTCCGACTCAGACACCGAATCAAATCTTGATTTTGCCACCATTCTCGCCTCCCAACGTCTCTTCTTCTCCTCCCCTGGCCGCTCCAACTCCATCATTGAGTCCTTGCCAGAGCCCCAGACACCAGTCAGTGGAGGTGTTGCGATTAAAAAGTATTCACCAGATCCTTATACGGATTTTAAACATTCAATGCAAGAGATGATTGAAGCAAGAGAGCTAAGGGACGTTAGGGCTAAATGGGACTACTTGCATGAGTTGCTCTCTTGTTATCTTAAATTAAACCCTAAACACACTCACAAGTTTATCATTAGTGCTTTTGCTGACATAGTAGTTTGCTTATTGTCTTCGCCCTCGCAGGAATCCGACACACAACGGGAACCTGACGGCCTTCGTCGGTGA

>PtOFP20 | Chr08:931154..932161 forward

tccttccatccccccctactcctcctcgcctcttctttaaatattaccctcacaacttcctctgatcacattaataccaaaccaaaaagcaacagacattcacaagccctgataaaaccattcaagctttctcttgctagtgtttgagatcaagaaatATGAAGATCCCTGCACTGTTCAAGATCAAAGAAACAAAGCAATCATGGCAAAAATGGCCTTCATGCAAGCACCCCAAGACTCTTTCTTTCAGGGGTGGAGATGATGTGATCAAGACTGTAAATTCAGTTTTCTTTGATCCTTCTGAAAGGGTTGAGACACCAGAGTCATGGTTCACAAACTCATCAGAAACTACAAGTTTCTCAACTGAGTCGGAGGGCTTTGATGGGGAGTCGTTGGAGGTTGTTGTACGTGGGGTGAGATCAGAAAGGTTGTTTTTTGAGCCTGGAGACACAAACTCAATACTAGAAGAGGCAAAAACAGGAGGGTTTCCATTCAAAGAAAGTGTGGTTCTAGCAATGGAGTCAGAGGATCCATATGTTGATTTTAGAAGGTCAATGGAGGAGATGGTGGAGTCTCATGGACTTAAAGATTGGGATTGTTTAGAGGAGTTGTTGGGGTGGTATTTGAAGGTCAATGGGAAAAAGAATCATGGGTATATAGTTGGGGCATTCGTGGATCTACTTGTTGGGATTGCAGCTGCTTCTTGTTCTGATTCCACTTCCTTTTCTTCTGCTGTTTCTTCATTTTCTCCTTCATCCCCTTTATGTTCATTGAAAGGGCAAAATGAGATTGATGAGGAATAAcagatggtatagtcttagcttccttatatcagttttgtgtatattagtaggattaacgagaagcaaaaatgagatacagagtttgtttttgtttatattactacgatgactatgaaatctatccccttcttggaaatgtcaagaaatattagaatatgaaggttctgcgatcttgttctgtgtctgtgaatatccaggtaggtcc

>PtOFP21 | Chr10:11142835..11143918 forward

ctccaccgttcaacgttcctctatttttatccccattgccattatagcagacacattacaatctccctgtctgatctcatcaagaggaaaaATGCCCACCATCTTCTGGAAGAACATTCTCAAATGCTTACCCACCATAACCCCGTCCTCACATCCATTGCCCTCAGAGTTGCAAGAGCATAGTGATCCATTACTATCATCCGCTACCGCCGCCGCCCCCACCACATCAGTAATGATCAAGAACTTCAACTCCCTCTATGACCTCTCCTCAGCATCCACCTCCAAATCCCTCAGTACTCCCTCCACCAACTCCTCCTCCTCATCTTACTCCGATCCTGACACCGACTCGACGCCTGATTTTGCCACCATTATCGCCTCACAACGTTTCTTCTCCTCCTCCCCTGGCCGCTCCAATTCCATCATTGAGTCCATGCAAGAGCTTCATACCCCAGTCAGTGGAGGTGTTGCCATTAAAAAGTATTCACTAGATCCTTACATAGATTTTAAAAACTCAATGCAAGAGATGATTGAAGCAAGAGAGATAAGGGACGTTAGGGCTAATTGGGACTACTTGCATGAGTTGATCTCTTGCTATCTTAAATTAAACCCTAAAAACACCCACAAGTTTATCATTAGTGCTTTCGCTGATATAATTGTTTGCCTATTGTCTTCGCCCTCGCCGGAACCCGACACCCACTGGAAACCTGAAGGCCTTCAACAACACAAGGTTTCACGTTTATTGGTGTGAggtcaattatgtactgtgtacatcttttaaattttgttttgttattttaaaagaaggggcatatgtgatgtatgttagtggtggtacgttttttcgttgttttgtggaatcactttgtatttggtgaggagggatcgatgttatcatgcatgaagacacagtaaaggtcatttgtaatggaggcaaagtaaattataaataatttggaaaccaaggaattcgcgtgggatgttaatgtctcgatgatgttttaatttttaatctttgtcttgtgttgggttatatgcatgtgtttaagtttttcttgttaagtgtttctaggtactttggatggat

>PtOFP22 | Chr10:21845743..21846472 forward

aacaATGAAGATACCAACACTCTTCAGGGGCAAAGAAACAGAGCATACATGGCAAAAATGGCCTTCATGCAAGCACCCAAAGACTCTTTCTTTCAGGGCTGGAGATGATGTTATCAAGACTGTAAACTCAGTCTTCTTTGATCCTTCTGAAGGGGTTGAAACACCAGAGTCATGGTTCACTGACTCGTCAGAAACTACAAGCTTCTCAACTGAGTCGGAGGACTATGACGGGGAGTCATTGGAGGTTGTTGTACGTGGGGTGAGATCAGAAAGGTTGTTTTTTGAGCCTGGTGACACAAACTCAATACTAGAAGAGGCAAAAACAGGAGGGTTTCCATTCAAAGAAAGTGTAGAGCTAGAAATGGAGTCTGAAGACCCATATGTTGATTTTAGAAGGTCAATGGAGGAGATGGTGGAGTCTCATGGACTTAAAGATTGGGATTGTTTAGAGGAGTTATTGGGGTGGTATTTGAAGGTCAATGGGAAGAAGAATCATGGGTATATAGTTGGGGCATTTGTCGATCTACTTTGTGGGATTGCAGCTGCTCCTTGTTCTGATTCTACCTCTTCCTCTTCATCTCCTCTATGTCCATTGAAAGGGCATAATGAGATTGATGAGGAAGAACAGATGGTATAGtcttagctttcatgattccagttttgcttatatcagtaggatttaagaaaaacaaaatgagattagagcttcttttcgtgtacattaattact

>PtOFP23 | Chr14:15283281..15283520 reverse

ATGCACAGTTGGTGGAAAAGCGTGGCGGTTGCGAAGAAATCACAGGATCCATGCCGGGACTTCAAGAGGTCTATGTTGGAGATGATATTAGAAACGCAAATATTTGAGGCTGAGGATTTAGAGGAACTTTTGCAGTGGAGACAGAGTCATGGAGTTATTGTGCAGGCATTCTTGGAGATTTGGGAGTTCGTGTTACGTGATTCTCATGTAAAGAAAAAAACAATATTTTGGTGTTTTTAG

>PtOGP24 | Chr15:289125..289620 forward

tcattacacgctccagctatcacatgactcctaaatatatatatagagtatcaggtcatggttttggtcctgaatggtacttaatttagtcattattcctgagtgtaggagccATGGATGCTGGAGAAGACTACAAAAGGAAGGCAATTACAAGGAAGAAGAGTTCTTACAGGATTAGCCTTTCGGCTAGTTTACCTGAAGATGTTTGTGGTGCTTTCTCAGGTGATACTATCTGTGCAGTTAAGCTCTCTAAGGATCCATTCTCAGACATGAGAGCATCAATATTAGAGATGATTCAGAACGTGGGTGTTCATGATTGGGATGAAATGGAAGAGTTAGTCTATTGTTACATTGCCCTTAACTCTCCTGACCTGCATGGCATCATTGCCAATGCATTTCTTAGTTTATCTTGTCATTTTTCCTAGctcgtacgctctttctctgttgtctgcaacatactgcttaaaatggcacatctagtcaacttctttttagt

>PtOFP25 | Chr01:15631731..15632618 reverse

ATGCCAAAGAAACTCCAAAAGTCTCTCCAAGACTATATCTATAAGATCAAAAACCCTACCCAAAATATCCAATTATCTTCTGATTCTTTCTCAAATTCAAAGAACTGGATATTAAGAGGCTGCAAACACCCTAGAACACTATCCTTTGCCATAGCTGGTAACCAGAATAAGAGTCGTGATGAAGAAGACGAGGAGAAGGGTGGCGCGGCAACACTCTCTGATGTGGATCGCTTCCTTTTCGAGAATTTTAGGTCACTTTATATCAATGATGACGATGGGAATTTTCAAAAGGAAAGTGATCGTAGATCTAGAGGAGGTGACCAAGCTCCGAGCATGAACGAGATTTTAATTGATTCACCTAGGTATATTGATCAACCACTGGACCTGTGTGGCTCCCATCGATTCTTTGTGGAGCGGGGCTCATCATCCGGCTCACTTGTGGAAGAGGCACGGTCCAGCCTAACTGCCACCTCCGAGAACATGGGTTCAAGTTCAAGTTCAAGTTCAACCTCAGTCTCAACCACCAGTACCCTCAATGACGACTCTGCCACAGTTGCTTCAAATGATCCAAAGCAGGTTAGGCTACCAGATGACTGCATCGCAGTGCTAACCTACTCTCCGAGTCCCTACGATGATTTTCGGCGGTCCATGCAAGAAATGGTGGAAGAAAAATTGCAGAATAACGGTAAGGTTGATTGGGATTTCATGGAAGAGCTTTTGCTATGTTACTTGAACTTGAATGAGAAAACGTCCCACAAGTTCATACTCAGTGCTTTCGTGGACCTCATTGTTGGTTTGCGCAAGAATCCGGACAAAGTTCCTGTCAGATCACGCCATTCTCGAATTGCAAGATCAGGGGGGAGGAGGAAATTGGAAAATGTAACGTAA

>PtOFP26 | Chr02:3377480..3378805 forward

ccagcttacagtcttttaccgttgtctagtcgccttcgaaccaaaactacagaaactagtaacaacttgcaccatcaaggcattaaccatgatggaccctcccctctatctctctgaaccgacacgaataaatacagcatcacctgcccaatttctttttgtttatttatctttgattcacatttcctcctctctctttcccacacaatcagaaaaataaaataccgagagaagaggagaaagaATGGCTGGCACAGCAGGAAGAAACCTCAATCTCTGCTTCATCAATAAGATCAAACGTCCACTACCACCTGATCATCAACCCCCCTCTAACCCACTAACCCCAGATGATCACAGCCATCCATTCCTCTTTAAAAACTATAATTCCCTCTACGACCACACCATTGACTCCGCCTCCGCCTCCACCTCCACCTCCATCTCTTCCAGCTCCTCCTCCTCTGAACCTGACTTCGCCAGCGTCTACGCCTCTCAGCGCTTCTTTTTCTCCTCCCCCGGCAGCTCCAACTCCATCATTGAATCTACACCGTCCATTGTCACTTCCACAGAATCATCGGACAATCTAGTAGCCCCGCAACCTGATAGCAATGGTCTGATAATAAATCACTCCACTGGCAAGTCTTTGTTACTTGACGGTTGTAACAATAGCCATCCTTTACATGATCAACAACCACCCCAATTATTAAAATCACCAACCGTTAAAGACAGTATGGCTGTCTCCACCTACTCACACGACCCGTACATGGACTTCCGGCGATCCATGCAGGAGATGGTAGATGCACGCGACTTGGTGGATGTCAAGGCTAATTGGGAGTACTTGCACGAGCTACTATCGAGTTATCTTTCTCTCAATCCAAAGAGTACCCACAAGTTCATTGTTGGTGCTTTTGCTGATCTTCTTGTTAGTCTTTTGTCAACGGAAATGACGGAAGATGGTGGCCGCCGGGAAGAGGATTTTTCTTCCGATGGTTGTGGGATTTCGCGGCAGTGCATATAAtgtagcaatgtggtgtgtcgggaaatgattgtataatcttttaactataatttagcaatatatacatgtggggaactaagatggccctcttgtcttcgatcacagcaccttatccctcacaacaaccatcttgcttttggtccttgtatttattaagctggtattttttttttcaatttgtttgtattatttccagttataaaaaaacctcgaggtccttaattatattttttctacgattgggtctcccaaacttgtgatcctttttttggaacgttgtagcttttttctcgaaaataaaataaaaatcg

>PtOFP27 | Chr06:22103710..22104857 forward

caagaacatgatgtgataaataaatatcaacaggcgagtcataacatcattcaggggcagagcatggacccatcttaactcatgcaggatttatagagaaccctcacctcccccagactcagtcaaatgcctcatctctactcatccacttcctcgggcactcttcaaggaaaatcccaacagactcctttcttctccctatctcttacgcgcatcttttagcaatcaacaATGTCCACTAACAAGAAAACGTTCCTTCTCAACACAGTCTCACTGAACTTAGGCTGTAGCAGCTGCAAGAAACCAAAACTTTCCAACATATGCCAGCCAAAACCAAAACCAAAACCAAAACCAAAACTCCAAACCCCGACTTACCAAAAACACAAAAAAGATCTCTACTGCTCCTCTTCTTCAACCTCTTCATCAAAAATAACAACAAACCAATCTCCAAATGGCCATGAAAATCATGACACCCCGAACACATTTTCTCCAGCAATGGACACACCACCCCATTTCTTCTCTGACACAGGCAACAACATGAAGTGTTCAACAGCTGTACGAGGATTCGGTCGCGTTGGAGGTGAAAGTGTTGCTGTTGAGAAAGATTCTGATGACCCTTATTTGGATTTTAGACACTCAATGTTGCAGATGATATTGGAGAAGGAGATTTACTCGAAAGATGATCTTAGACAGCTTCTTGATTGTTTCTTGCAACTAAATTCGCCTTACTATCATGGGGTTATTATTAGAGCTTTCACTGAGATCTGGAATGGAGTCTTCTCTATGAGGACCGACACCACTAGTACAGGTTCCGAGAAGCAACTACATTATTACTATGGTTGCTAGtcacgtgatttatgaaatggttcttgctgttctgcacgtgaaagttctgtctgagcacaaaatattcctgtgtagaatgaatggttgcttaataaagtggaagtcttttttttttcttgttgacatgaaattttataagttagtttatgcatagtttggttttttgttcatgtaatggcaaaaaatgtctgagcgacatttctaacttttgtacatttgttggtttatgtaatggcaaaagtacaaatattagagttgtagaatcttatccatctatgcttttaatgaaaatttactttggg

>PtOFP28 | Chr13:15814307..15815550 forward

tgaggatcatttttaatttaatggaacttttggcccaaatgatgagcatccattgaactcttctccatatcaggattcttgccaaaatagtctctcacttcatggggtgccactttcctccttgaggacatgaaccctacaatagcaaggagatgcacacagggcttaactaacctcttattatatccatcttcaacaaactctctctccctctggtacaccaaccgttagtggagcaacattatgtccttgaactagctagaagcaataaaaggactccattctgctccatttcttcaacaacactctgatatcaatcattacagATGAAAATGAAAGCATTAGTTGTCTTCAGATCCAAGCTTTTCAGGCCATGCAAGAAACTACTAATACTCTTCAGATTCAAGCTCAAAGGACCTGTCTTTATAAGAGATCTTCGACTTCATCGTCGTAGCAAAAAACGCAGAAAAGCTCCTCAAAAGAGCCGAGTTTTTACTTTTTTTCGTTCTTTTAGGAAGTCAAGAAAGATGGACAGAGTTGCAGAACTTAGGAGCGTCTCCGAAGCAGAGCGTGAGAGAATGCTCTATCCATCTCCTCTCACACCAGCTTATATCAAGGCCAGTTTGGCCACAAAAAGGCAAACCTTTGGTGATGAAGATGTAGAAGATGCATGCAGAAGTTTCGAGAACTATTTGGTGGAGATGATGGTTGAAGAGGGAAAAGTAAGGGATTTAGCGGATGTGGAAGAGCTTCTGTATTGCTGGAAAAACCTCAAGTGTCCTGTCTTCATTGGTTTGGTCTGCAGATTCTACGGAGAGCTATGCAAGGACTTGTTCTCTCCTGATGTTGACAACACCGACGTTGATAGTCCCAAATCTCCCAAATGAttctgcgaatagtattgctgttcctcctttgcactgcagagacaatccctgtcctctccgcaatccctcttctggtttctctgttgactctttgtattgcgagtagataatgaaaggaataaatcccattgttgtatatcgttttctcttagtgttaggaggcaaatcaagcaaagggagagagcggtgaaattcgaggatgctgccaaagaattggaaagcacacgtttcgtactaattacaagattagtcattaactgatctttggtttccttctcattgatcaagtacaacattgaaaaaacactaggaaaattgagaaatagagctttatgcacctctatgttagtaagataa

>PtOFP29 | Chr19:15484122..15484694 reverse

ATGAAAGCATTAGCTGTCTTCAGATCCAAGCTTTTCAGTCCATGCAAGAAACTACTGTTACTCTTCAGATTCAAGCTCAAAAGACCTGTCTTTATAAGAGGTCTTCAACTTCGTCGTCGCAGCAAGAAACCCAGAAAAGCTCCTCAAAAGAATCGAGTTTTTAATTCTTTACTCTCCGTTTTTCATCCTCTTAGGAAGTCGAGAAAGATGGACAGAGTTTCCGAACTTAGGAGCGTCTCAGAACCGGAGTGTGAAAGAATGCTCTTTCCATCACCTCTTACACCAGCTTATATCAAGGCCAGTTTGGAAAAGAAAAGGCAAACCTTCGGTGATGAAGATGTAGAAGATGCGTGCAGAAGTTTCGAGAACTATTTGGTAGAGATGATGGTTGAAGAGGGGCAAGTGAGGGATTTAATGGACGTGGAAGAGCTATTGTATTGCTGGAAAAACCTGAAGTGTCCCGTCTTCATTGATTTGGTCGGTAGATTCTATGGAGAGCTATGCAAGGACTTGTTCTCTCCCGAGAGCTATGCAAGGACTTGTTCTCTCCCGATGACGACAACACTGACATAA

>PtOFP30 | Chr18:10677937..10679000 reverse

tccctctccctctctgctctctccccttcttctgaaatatctctctttctcccATGGAAAACCGATTTAAGACGCGAATCTCTCGCATGTTTCGTGGCTCATGTCGGACCCGAAACTTATCGGACGTGATTGAAAATGCTGTGTTTGTGCCTCAAACCCATAAGAATTTCCACATGATCGAGCCTTTGCCGCCCAAGGTTCGACCTTTCCCTTCTATTTGCAGACACAAATGCCCTGAAGCAACAAACCAAGTCCTCAATCACTCTATCATCTCCAGGCAAAAATTATCACACCGTTATCCTCCTCTTATAACTGCCAATACTAGTGGACACAGTTCTTGCCCTCCTGCTTACCCCATTTTTCCTTTGAATCCATTCTACAAAGACTTGAGTTTCAAAGAGAAAAAGAAGAGTTGTCGTTCAGTTAAAAACAGAAGCAAAAAGAAGAATATCATTAGCAAGAAAGAACAGACGAGTTTGTTCAGATCATCTTCACAAGATAGTACATATTTTGGAGGTAGCTATTATTGGTTTAGCAGCGAAGATGAGGACAAGAGAGGGGATGAGTCGGACACTCTTTTCTCTTCAAGAAGTCTTTCTTCGGATTCATCAGGATCTCTTAGCCACCCTTCTCATGGAAAAAAGTTCACTTCTCGGAGGAGAAGGGCCAAAGTGAAGAGTTCTCATGTGGGTGTTTTGCCATTAGATGGCAAAGTTAAGGACAGCTTTGCAGTGGTGAAGAGTTCTAGTGATCCTTATAATGATTTTAGAACATCAATGGTTGAGATGATTGTTGAAAAGCAGATATTTGCGGCTAAGGATCTTGAACAGCTTCTGCAGATGTTTCTTGTCTTTGAACTCTTATCATCATCATAGGATTATTGTTGAGGTTTTCATGGAGATTTGGGAGGTTTTGTTCTGTAATTGGTCTTGAttatgagcagaaaataaaactgtactgcttgttagctttctgctatctttgttttaaagtatgtagtttagagttctaactctgataaactatgttaattaatagattttctttctctgttattttctcgat
