# Supplementary material for: Identification, Expression, and Interaction Analysis of Ovate Family Proteins in Populus trichocarpa Reveals a Role of PtOFP1 Regulating Drought Stress Response
Source: Front Plant Sci. 2021 Apr 20;12:650109. doi: 10.3389/fpls.2021.650109 (PMC8095670; doi:10.3389/fpls.2021.650109)
Supplement: Supplementary Table 3 — The CDS sequences of PtOFP genes. [file Table_3.docx]

**Table S3. The CDS sequences of *PtOFP* genes.**

>PtOFP1 CDS sequence

ATGGGCAACAATAGGTTCAGATTATCAGATATGATGCCTAATGCTTGGTTTTACAAGCTCAAAGAAATGGGCAAAACAAGAAACCATAACACCACCACTC

ATTCCATAAAGAAAAGACAAGCTACATCAGCTGCTGAAACTCAGCAGCCACCATCCAAACCAAAACATCCTCAATACAATCCTTATCCAAGAAAATCATA

CTACATCACTAGAGAGCTTATCTCAAGTGAACAAATACCTCACACTTCTCCAAGAAACTCAAAATCCACGTACACCAATTTTCCTGACCCCCCAAGAAGA

TCATCGAATCAAAGAAACAGGAGAAGAACCATCAAGGCCTCTCCTAAGCATGTCTCTGCAGGTTGTAACTGCCGTGCTACGCTCTGGACTAAATCAGATT

CTCCTCCAGACTACTCAGCTTCTCTTTATGATGGTTCTCTTGACCAGGAGACAGATTTCTCTGACTCATTTCCACCAGAATTCAAGTCGGACAGTGCTCT

TGCTACCGTCTCATTTGATAAAATGTTGTCTTGGTCAAGCTCTTGTGACTGCAAACTTGATTCTATTGACAATGATGGCATTGTTATCAGTGTGGATAAG

AAGTCTACTGCTAGGAATTTAGATAATCCGAAAGTGTTTCATAGCATATCTGATCTTGATCTTCCTCCAATCGTAACAAAGCCTGCTAAATTTGATGATC

AACTTGAAGATACCAAGAAGAAAGAAACCCAAGAACCAACCAAGTATAGAAGGAGTCCAGCTAAATACGAGGAAACAAATGCTCATGCCTCTTTATCTGT

CAAGGTTGTAAAAGAAGAGAGCATTGCAGTGAAAGAATACAAGACCAGTTCTGTCCGGAGAAATTCTGTGACTTCACCAGGAGTCAGGCTAAGAGTCAAT

TCTCCAAGAATCTCAAATAAGAAAATCCAGGCTTATAATAATGGTCGAAAGAGTGTGTCATCGACGACAAGTTCATCGTCGCGGTCGCGAAGAAGCCTTT

CGGATAGTTTGGCAGTTGTGAAATCTTCTTTTGATCCCCAGAAAGATTTCAGGGAATCGATGGTGGAGATGATAGTGGAGAACAATATCAAGGCATCAAA

GGACTTAGAAGACCTTCTCGCTTGTTATCTTTCACTCAATTCTGATGAATATCATGACCTTATTATCAAGGTGTTCAAGCAAATTTGGTTTGATCTTACA

GACATCAAGTTGAATAGTTGCGCGACTACATGTGAATCTACTTACAAGTTAAAGGCAGTATATTCAGCTTTCATAACACTCCCTTTTCCAGAGAAGAACA

CTAAAGAAAGAGAACAAAAGCTGCTGATTCAAAAATGTTTAAAGCTGATCAACAATGGTTAA

>PtOFP2 CDS sequence

ATGTTTCGAGGCTCATGTCGGACCCGAAACTTATCGGACGTGGCTGAAAAAGCTGTGTTTGTGCCTCAAAACCATAAAAACTTCCACTTGATCGACCATT

TACCTCCCAAGGCTCGACCTTTCCCTTCTATTTGCATACGCAAATGCCCTGAAGCAACAAACCAAGCCATCAATCCCTCTATCATCTCCAGGAAAAACCT

ATCACACCGGTATCCTCCTGCTTCCCCCATTTTTCCTATGAATCCATTCTATAAAGAATTGGGTTTCCAAGAGAAAACGAAGGGACGTTGTAGCTCAATT

AGAAACAGGAGTAAAAAGAAGAAGAACATCACTAACAAAAAAGACCAGATGAGTTTGTTGAGCTCATCTTCACAAGACAGTGCATGTTTTGGAGGTCGCT

ATTACTGGTTCAGCAGCGAAGATGAAAACAAGAGAGAGGATGATGAGTCGGACACTCTTTTCTCTTCAAGAAGTCTTTCTTCAGATTCATCCGGATCTCT

CAGGCACCCTTCTTCTCGCCGCAGAAAGTACACTTCTCGGAGGAGAAGGGCAAAAGTGAAGAGTTCTCAAGTGGGTGGTTTGCCATTAGATGGAAATGTG

AAGGACAGCTTTGCAGTGGTGAAGAGTTCTAGTGATCCTTATAACGATTTTAGAAAATCAATGGTTGAGATGATTGTTGAGAAGCAGATATTTGCAGCTA

AGGATCTCGAACAGCTGTTGCAGTGTTACTTGTCTTTGAACTCTTATCATCATCACGGGATAATTGTTGAGGTTTTTATGGAGATTTGGGAGGCTTTGTT

CTCTAATTGGACTGCTTGTTGA

>PtOFP3 CDS sequence

ATGAAGCTCCCTTTTCTCTCCAAGATTAATACAAACACAGATCAATCAAAAAGGCCTTTATGGCCTTGGCCTACTTATTGTCACCAACCAAGAACCCTCT

CTTTTAGTTTTAGAACAAGTGATGGCATGTTCAAGACCATCAACTCAGCTTTCTTAGATGCTACTAACAATGATGTGGTGGACAGCACACCTGAGTCATG

GTTCACCAAATCTTGTGAATCAGCTAGCTTCTCAACAGCATCAGATGATCAGTCAGGAGCTATTGACCCTATAGAGACAGTAATCAGAGGTTTAAGGTCA

GAGAGGCTGTTCTTTGAACCAGGAGAGACTAATTCAATCTTGGAAGAAGCTAAGGCAGGTGATGAGTTTCCATTTAAAGAAACTGTGGTGCTGTCAATGG

AATCTCAAGACCCTTATTTGGATTTCAAGAAGTCAATGGAGGAGATGGTTGAGGCTCATGGTTTAACAGACTGGGAAGGCCTTGAAGAGCTTTTGTCTTG

CTACTTGAAAGTGAATGGAGAGAGCAATCATGGGTACATCGTTAGTGCTTTTGTAGATTTGCTTGTTGGTCTTGCTTTTGCTTCTTCTTCTTCCTCCTCT

TCTTCTATTACTAGTACTAGCACTACTCAACATCATCATGATTTTTGTTCTTCTTCTCATCATTCTCCTTCTTCTCCTTTGTCTTTATACACTTCTTCTA

CATCTGATGATGATTCTTCTTCTACTCCTTGCTGTGTATCCTCATTAGAAAATGGGGCTGATATAATTAGTCCCTGTTTAACTTCATTAGAAGCTGAAAA

TGGGATTAAAAATATTAATCAATAG

>PtOFP4 CDS sequence

ATGGAAGACATTGATTGGCATTTCGCATTATCCTCTCAACACTCTCAAGGCTCAGCCAAAGCTTTTGACATAGACAATCCAACAATTTTTTCTGTTCCGC

TAGCCACTATTTTCACTCACAAATACACAGAAAAAGAGGGTGAGGGGAAGGGAAGGGTGGTGTTTTTGATGGGCAACAATAGGTTTAGATTATCAGATAT

GATGCCCAATGCTTGGTTTTACAAGCTCAAAGACATGGGCAAAACAAGGAACCACAACACCACCACTCATTCCACAAAGAAAAAACAAGCTACATCAGCA

GCAGCAGCAGCTGTAGCTGAATCTCAGCAACCACCGTCCAAGCCAAAACATCCTCACTACAATTCTTGTCCGAGAAAATCATACTACAACACCAGGGAGC

TTATCTCAAGTGACCAAAAATTTCACACCTCCCCAAGAAACTCAAAATCCACAGACACCCTTTTTCCTGACCCACCAAGAAGATCATCGAAACAAAGGGC

TAGGAAAAGGACCATCAAGTCCTCCTCTCCTAAGCTAGCCACCTCTTCTGTCTCTGCCGTTTGCAACTGCCGTGCTACCCTATGGACTAAACCAAATTCT

CCTCCAGACTACTCAGCTTCTCTTTCTGATAGTTCTCTTGATCAGGAGACGGATTTCTCTGACTCATTTCCACCAGAATTCAGGTCTGACTGTGTTCTTG

CTACTGACTCATTTGATAAAATGTTGTCTTGGTCAAGTTCTAATTGTGACTGCAAACTAGATTCTAATAACTATGATGATATTGTTATCAACATGGATGA

GAAATATATTGCTAGGAGATCAGATGATGTGGACGTGTTTCACAAAATATCTGATCTTGATCTCCCTCCCATCATAACAAAGCCCCCTAAATTTGATGAT

CAAGTGGAAGATTTCAAGAAGAAAGACACCCTGGAACCAGTCAAGTATAGAAGGAGTTCAGCTAAATATGAGGAAACAAACGCTAATGCTTCTTTATCTG

TTAAGGTTGTCAAAGAAGGGAGCATTACTGCAATGAAAGAACATAAGACTAATACTACTGTCAGGAGAAATTCTGTCACTTCACCAGGAGTTAGGCTAAG

AGTCAATTCTCCAAGAATCTCAAATAGGAAAATCCAGGCATACAATAATGGTCGAAAGAGTGTGTCATCAACTACAAGTTCATTGTCTCGGTCGCGAAGA

AGCCTTTCGGATAGCTTGGCAGTTGTGAAATCTTCTTTTGATCCTCAGAAAGATTTCAGGGAATCAATGATGGAGATGATAGTGGAGAACAATATCAAGG

CATCAAAGGACTTAGAAGACCTTCTTGCTTGTTATCTTTCACTCAATTCTGATGAATATCATGACCTTATTATTAAGGTGTTCAAGCAAATCTGGTTTGA

TCTCTCTGACATCAAGTTGCAGTGA

>PtOFP5 CDS sequence

ATGTCCACTAACAAGAAAAAGCACCTTTTCAACACAGTCTCGGTGAACTTAGGCTGTAGCAGCTGCAAGAAACCAAAACTTTCCAATATATTCCAGCCAA

AACCAAAACTCCAAACCCCCACTTACAGAAAACACAAGAAGGATCTCTACTGCTCCTCTTCTTCGACCTCTTCAACAAAAATAACCACAAACCATTCTCG

AAATGACCATGAATATCATGACACCCCTTCCACATTCTCTCCATCAATGGACACACCACCCTATTTCTTCTCTGACACGGACAACAGCGGGACGTGTTCA

AGAGCCGTGCGAGGATTTGGGCGTGTTGGAGGTGAAAGCGTTGCTGTAGAGAAAGATTCCGATGACCCTTATCTGGATTTTAGACACTCAATGTTGCAAA

TGATACTGGAGAAGCAGATTTACTCAAAGGATGATCTTAGACAGCTCCTTGATTGTTTCTTGCAGCTAAATTCGCCCTACTATCATGGGATTATCGTTCC

GAGAAGCAGCTGCATTATTACTATGGCTGCTAGTCACGTGACTTGTGAAAATGGTTCTTGCTGTTCTGCACGTGAACGTCTTGTCGGGGTACAAAATCAT

TCTAAGGATGATCTTAGACAGCTCCTTGATTGTTTCTTGCAGCTAAATTCGCCCTACTATCATGGGATTATCGTTAGAGCTTTCACTGAGATCTGGAATG

GAGTCTCCTCTGTGAGGTCTAATACCACTACAGGTTCCGAGAAGCAGCTGCATTATTACTATGGCTGCTAG

>PtOFP6 CDS sequence

ATGAAGCTCCCTTTTCTCTCCAAGAATAATGCAAATACGGATCACTCATCAAGAGCTTTATGGCCTTGGCCTGCCTATTGTCAGCAACCAAGAACCCTCT

CTTTTAGTTTTAGAACAAGTGGTGGAATGCTCAAGACCATTAACCCAGGTTTCTTAGATGCTACTAACACTGATGTGGTGGACAGTACTTCACCTGAGTC

ATGGTTCACCAACTCCTGTGAGTCAGCTAGCTTCTCAACAGCATCAGATGATCAGTCAGGAGCTGGTGAGTCTATAGAGACAGTGATCAAAGGCTTGAGG

TCAGAGAGGCTGTTCTTTAAACCAGGGGAGACAAACTCAATCTTGGAAGAAGCTAAGGCAGGTGGTGAGTTTCCATTTAAAGAAAGTGTGGTATTGTCAA

TGGATTCTCGAGACCCTTATTTGGATTTCAAGAAGTCAATGGAGGAGATGGTTGAGGCTCATGGATTAACAGACTGGGAAGGTCTTGAAGAGCTTTTGTC

TTGCTACTTGAAGGTGAATGGAAAGAGCAATCATGGGTACATCATTGGTGCTTTTGTAGATTTGCTTGTTGGTCTTGCTATTGCTTCTTCTTCTTCTTCT

TCAAGTACTATCACTACTGCTAGTACTACGCAACATCATCATGATTCTCCTTCTTCTCCTTTGTCATTTTACACTTCTTCTGCATCTTCTGATGATTCGT

CTTCTACTCCTTGTTGTGTATCCTCGTTAGGAAATGAAGTTGATATAATCAGTCCTTGTTTAACTTCATTAGAAGCTGAAAATGAGATTAAAAAGTATTA

A

>PtOFP7 CDS sequence

ATGCTGAACTTTTTTCTAGTGAGCCCCCATCTATTCTTTACCTTTTATAACCCAAACTTTGTAAGCTCTTTTTATCTTTTCAGTAATCAAAGAGAGGGAA

AGGTAAACCAACTCAGACAAATCTCAGAGAGGGAAAGAAAAATGGGTAATTACAGGTTTAGATTATCAGATATGATACCAAACGCCTGGTTTTACAAGCT

CAAGGACATGAGCAAAAGCAGAAAGCATTACACCTCTCGAGCTTCCAAGAAAAAACCACCTCCAGGAACTGTAACATCGCAAAAACCCAACATTTCTCAT

CAAAGATACTCTTATTACTTCACCACAGAACCTGAAAGAGCTGAGAAATTAGATTACTATTCACCTGCAAATCCAAAAGCCTCAGATACTCACTTCCCTG

ATCCACCAAGAAAATCATCCAATAGAAGAAACAAAAGAAAAACCATTTACAAGCCTTCTCCAAAACTGGTGTCCACATTCTCTGCTGATTGTAGCTGTCG

CGTTACAGTTAACTCGAACCTGACCGGGTTTATTCCAGGTGATTCACCTGACTGCTCTAGCTCTCCTGTTGAGAGCTCTTATGATGAGCTTGACTTCCTA

TCAGAATCTGATGAAGATGATGGTTTTCTTGTTCCTGATTCAATTGATCATCATTTAGCCTCCTGGTCTAGCTCTTGTAACTGTAATGTTAGCTCTTCAA

CCACTGACATTATCATTGATATGAATGAAGAATCCTATGAAAGGAAAATCAAGGAGGTTGAAGGGTTTGGTAGGATCCCTGAGCTAAACCTTCCTCCTAT

ACTGACCAAGCCTGAGAAATTCAACGACAATGAAGTTACTAAGTTCAGAAGAAGTTCATCCAAATTGGAGGAGGTCAAAGCACATAGGTCTCTCTCTGTC

AAGATTGTCAAGGAGAAAAGCATTAGGACTTATAAAGAGAAAAGGATGAATCCTCCTACAAGAAAGTCCTCGGTGAATTCGGCAAAAGGGATAAAACTCC

GAGCAAATACTCCAAGAATTGCAAGCAGGAAAATTCAGGGTTGTTCTCGAAAAAGTGTGTCATTGTCAAGAAACAAGACCCTTTCAGAGAGCTTTGCAGT

GGTTAAGTTCTCAGTTGATCCACAGAGAGACTTCAAGGATTCAATGGTGGAGATGATTGTGGAGAACAATATCCGAGGTTCAAAAGATTTGGAAGACCTT

CTTGCTTGTTACCTTTCTCTCAATTCAAAAGAATACCATTATATCATTGTCAAGGCATTTGAGCAAATCTGGTTTGACATGACTGACCTTCACTTGTAA

>PtOFP8 CDS sequence

ATGGGCAATTACAGGTTTAGATTGTCAGATATGATACCAAATGCCTGGTTTTACAAGCTCAAGGACATGAGCAAAGGCAGAAAGCAATACACCTCTCAAG

CTTTCAAGAAAAAACCACCCCCAGGAAATGTAACATCACAAAAACCCAACATTTCTCATCAAAGATACTCTTATTGCTTCACCACAGAACCTGGAAGAGC

TGAGAAATTCCATTTCAATTCACCTGTAAATTCAAAAGCCTCAGATACTCACTTTCCTGATCTACCAAGAAAATCATCCAATAAAAGAAACAAAAGAAAA

ACCATTTACAAGCCTTCTCCAAAACTGGTCTCCACATTCTCTGCTGATTGTAGCTGTCGCGTAACGGTTAACTCAAACCTGACCAAGTCAATTCCAGGTG

ATTCACCTGACTACTCTAGCTCTCCTGCTGAGAGCTCCTATGATGAGCTTGACTTCTTATCAGAATCTGATGAAGATGACGGTTTTCTTGTTCCTGATTC

AATTGATCATCAATTATCATCCTGGTCTAGCTCTTGTAACTGTAATGTTAGCTCTTCAACCGCTGACATTATCATTGACATGAATGAAGAATCCTATGAA

AGGAAAATCAAGGAGGTTGAAGGGTTTGGCAGGATCCCCGAGCTAGAGCTTCCTCGAATATTGACCAAGCCAGCAAAATTTAACGACAAGGAAACTGAAG

TTACTCAGTTCAGAAGAAGTTCATCTAAATTGGAAGGGGTAAAAGCACATAGGTCTCTCTCTGTCAAGATTGTCAATGAGAGAAGCATTAGGACTCGTAA

AGAGCAAAAGAACAATCCTCCTACAAGAAAATCCTCAGCGAATTCGACAGGGATAAAACTCCGAGCTAATACTCCAAGAATTGCAAGCAGGAAAATCCAG

ACTTGTGCTCGAAAAGGTGTGTCATTTTCAAGAAACAAGACGCTTTCAGAGAGCTTCGCAGTGGTTATGTCCTCAGTTGATCCACAGAGAGACTTCAAGG

ATTCAATGGTGGAGATGATTGTGGAGAACAATATCCAGGATTCAAAGGATTTGGAAGAACTTCTTGCTTGTTACCTTTCACTCAATTCAAAAAAATATCA

TGATTTCATTATCAAGGCATTTGAGCAAATCTGGTTTGACATGACTGACCTTCACTTGTAA

>PtOFP9 CDS sequence

ATGAAGTGGGGTAGGAAAAAAACCCCATCTTCTTCGCGCCCTTCTTTAATATCTCATGTTTTCCCCACTTCCTGGCTAACAAAGTTCAAGCACATGAGCA

TCAACCCAGGGCAAGAACATGCAAAAGCGAAGCAAAAAGGGAAATGGAATTCTGTTTCCGCAAGTCCATTGCCATTTGCTCGTGGTGAAGGAGGAGGTAG

ATTCTATGGAGGGGATGGCGATGCTTTTTGGAGACTTTCTTTTGGTGACGAGAGTGCTAGCACAGGTGCTTTGAGTTCATTTCATAATGATTTGGACAGT

GAGCTCCAGGCTCCACCATCAAGTTGTCACAGCTGTAGATCAAACGCTACAAGGGTAAATAATAGAAAAGAAGACAAGATCAGGTTCAGCAACAAGGTTT

CTGAAGCAAGGAAAATGAGAGGACTGCCAAGAGAAATTGAGATTTTGCCTGAAATGGATGCATGTATAAGTGAAAAGGTAGCAGAAATCAGGACCCCAAG

GTTGAGGGTTGGGAGAGAGGAGAAATTGAGGAAAACAGATCAAAGGGTTTTCGAAGCGCAGCAGTTCAATTTGGATGGAGAATCGTATGAAGCAGAGAGG

GTATCAAGAAAAGAGACATCAAAGAATATTTCTGAAACGGAATCAGAAAGAACGATTGGGAGGATAGAGAGAGAGGACTGTAAGTTGACAGCTTCTCATT

CAAAAAAAGATTTCTCCACTCATCTAAGAAAGACTAAAAAAGACTTTGTATTTGCTGCTCAGAATGAAAGTGATGGATTTTCTGCTGAAAATTTGAGTTC

TGAGTGGCAAACGTTGAAAGACATGAAGATTGAAGAGCTCAAGACAAAGAGGGAGAAACAGAGAAAATCTCTGTACATAAACAGAGAATTGCAGAGGAAA

AAGAAAAGTAAAGTCAGGGCTATTTCTCCAAGAACAGCTTCAAAAGTCGAAATCTGCAGAATAAAAGCACTTGAAGACATGAAGAAAGCCAAGATGAAAA

AGAAGAAAAAGGCTAGGGAGAAAAAAATGGAGGGATTCACAGGCCTAGAGAATTTCGCTGTGGTGAAAACCTCGTCTGATCCACAGAAGGACTTCAGAGA

TTCAATGATTGAAATGATTGAGGAGAAGAGAATTAGCCGGTCAGAAGAGCTCGAAGAACTCTTGGCGTGCTATCTGACATTGAACGCTGACGAGTATCAT

GATCTTATTGTCAAGGTATTCCGGCAGGTATGGTTTGATCTGAACGAGGCCTGCTCTGATACTGAACTAGAGAATGAACAAGGTTACGATGAATAA

>PtOFP10 CDS sequence

ATGAAAGAATCCAGGGTAGACAAGAAGCAAAAACTTCAGCAAAGAGGATGCAAGGCCTTTTGTTGCAGTTGCAGGCTAAGTGTCTCTTCTTCTGAGGAAG

CTGAGAGCTCGAATCCCGATCGGTTTGCATCGATCTCAAGCCTGGCACATGCGATGGTACAAGAGAGACTAGACCAGATGATTAGAGAAAGGCAAGAGGC

AAGACAAAGAGAGAGAAGAAGGAGACTAAGAAGTGATGGGACCAAGTTTATAGTAATGGTAGCTATGGAGAAAAGCTCTTACGATCCAAGAGAAGATTTT

AGAGAGTCCATGGTTGAGATGATCATGGCAAACCGGCTACAAGAGCCAAAAGATCTCCGTAGTCTGTTGAATTATTACATGTCAATGAACTCTGAAGAGT

ATCATGGAATGATACTGGAAGTTTTCCATGAGGTTTGCACCAATCTGTTTTTATGCTGTAAATGCCATTGA

>PtOFP11 CDS sequence

ATGTCTTCAAAGAAGAAAAATCTCCTTCAATCCATACTCACACCCAATGCAGGCAGTGGCTGTGGCTGTGGCAGGCCAAAACTCTCAGATGTATACGAGC

CAGCACCAAAACCTAAACCCAAAACCTCAATTTCCAAAAAAGATCCGAACCCTAAACATTGTTCCTCTACAATAACCTCATGTGATAAGAGTGTAGGCTT

CTCATTACCGGACAGTGAAGAAGAAGGCTCCACTTCCACTACTTTCACTTTGAAAAAAGACAACAACACGTCATCAACCCAAAACTCCGAATCCGAAACC

TATCCAAAAGCATCCAAAATCACTGACAGCATTGCTGTGGTGAAGGATTCTGATGACCCATTTCAAGATTTCAAGAATTCTATGTCGCAAATGATCTTGG

AGAAAAACATCTACTCTAAAGATGATCTTGAAGAGCTTCTTAACTTCTTCTTGGAGCTGAATTCTCCTTGCCAACATGATGTTATCGTTCAAGCTTTCAC

TGAGATCTGGAAAGAGATCAGATGTTTGTCACATGAATCCTAG

>PtOFP12 CDS sequence

ATGGAAAACCGATTTAAGACGCGAATCTCTCGCATGTTTCGTGGCTCATGTCGGACCCGAAACTTATCGGACGTGATTGAAAATGCTGTGTTTGTGCCTC

AAACCCATAAGAATTTCCACATGATCGAGCCTTTGCCGCCCAAGGTTCGACCTTTCCCTTCTATTTGCAGACACAAATGCCCTGAAGCAACAAACCAAGT

CCTCAATCACTCTATCATCTCCAGGCAAAAATTATCACACCGTTATCCTCCTCTTATAACTGCCAATACTAGTGGACACAGTTCTTGCCCTCCTGCTTAC

CCCATTTTTCCTTTGAATCCATTCTACAAAGACTTGAGTTTCAAAGAGAAAAAGAAGAGTTGTCGTTCAGTTAAAAACAGAAGCAAAAAGAAGAATATCA

TTAGCAAGAAAGAACAGACGAGTTTGTTCAGATCATCTTCACAAGATAGTACATATTTTGGAGGTAGCTATTATTGGTTTAGCAGCGAAGATGAGGACAA

GAGAGGGGATGAGTCGGACACTCTTTTCTCTTCAAGAAGTCTTTCTTCGGATTCATCAGGATCTCTTAGCCACCCTTCTCATGGAAAAAAGTTCACTTCT

CGGAGGAGAAGGGCCAAAGTGAAGAGTTCTCATGTGGGTGTTTTGCCATTAGATGGCAAAGTTAAGGACAGCTTTGCAGTGGTGAAGAGTTCTAGTGATC

CTTATAATGATTTTAGAACATCAATGGTTGAGATGATTGTTGAAAAGCAGATATTTGCGGCTAAGGATCTTGAACAGCTTCTGCAGTGTTTCTTGTCTTT

GAACTCTTATCATCATCATAGGATTATTGTTGAGGTTTTCATGGAGATTTGGGAGGTTTTGTTCTGTAATTGGTCTTGA

>PtOFP13 CDS sequence

ATGCCTAATCGTCTCAAGCATAAGCTCTCTCGTGTTATCACCCCATTCCAACTCTGTAGATCCAAAGATCCTTCTTGTCCCGAAGCTCCAATTCCAGCTA

TCAACAGGCTCTCTCCTTTCAACCCCAAAGCACTTGACATCAATTACCCTTGTAACCTCCAAGCACCACCACCGCCATCAACACCTTATTACAAATGCCG

AGTGTCAAGGAAGACCATTTCCGTCGGCTGCAAATGCCAATCGCGATCGTGCCCGCGCTGCTGCATGTCAGATTGGAGCATTGAATCGCCCGATTTTGCG

GGCAAGAAAGAAGCCAGGTGGCAAGCCAAACCTCACCTAAACGTGCCCTTCTCATTTTCTGATGGGAGTGGGGACATGTCACCCTTTATGGTAACTGGAA

AAAACAAAAACAGAGAAATTAATATCAAGAAAAACAAGGTCAAGACAGGTGTCTTGTCTGTTGATACTAGTGGGTGCTTTAGCAGTACTGATGTTGCTGG

TGAAGAAAATGAAACCCTACTTTGTTCTTCAAGAAGTTTCTCCTATGATTCTTCTTGTGAATTCAGCCATTCATTGGATACTATAGCCAGGCAATCAGAA

TATCATGAAGCGTTTAATAAGCCTATAGGGAACAAGAAAGTGAGTAACCTAAAGAAAATTAAAAAGCTTGGACACCAAATTTCATTGAACAAGTGGAAAA

GATCAAAGACTGTGACGTCTCCAGAGATTCCTTCACCTGTGAGATCATCCGTTTTGAAGCGGGTGATATCACGCAAGGTTGATGGGAGAGTGAAGGAGAG

CGTGGCAGTGGTGAAGAAATCACAGAACCCACACCGGGACTTTAAGAGGTCTATGTTGGAGATGATATTAGAAAAGCAAATATTTGAGGCCGAGGATTTA

CAGGAACTTTTGCAGTGTTTTTTGTCCTTGAATTCGAGACAGTATCATGGAGTTATCGTGCAGGCATTCTCGGAGGTTTGGGAGATCGTTTTCTGTGATT

CTCCTGTAAATAAGAGAGCTTCTATCAGAAATTAA

>PtOFP14 CDS sequence

ATGGCTAAACGTTTCAGGCTCAAATTCTCTCGAGTGATCTCCTTTCAATCTTGCCGTTCCAAAGACCCTTCTACTCTCCCATCAAATCCTGTCCCTTCAT

TTCTTAGACTATCTCCAGTCAACCACAACTCCATCATCATCAACAACCTCCATCTCCCACCCTCACAACCACCTCCTTCCAAACCTCTCCATCACTCTTC

CATCAGGCGACATGTGTCCTCGGCATTCACATCAATGGGCTGTGGGTTTAGATCAAAACCCTCCACACATTACCTCTCTGAAACCGACCACACCAAATCC

TCTCCACCAACTGAAAATTTCCACTGGGAAGAGGAAGAAAAATATCACGTTGTGGCCAAGCTCTTTGACGACGATTCAACACCCCGCCGCAAGATTTACA

ACTCTTCAGCCTCTGAGGACTCCAAAAACCACGACGTTTTTCTCCCTCCATCTAAAATCGAGAAGAAAAAACGACGAGTCAAAAAGAAGAAAACGGCATC

AAGAATCCGCATCAGCACTTCCTCAGCTGATAGCGGGATATTTTTTAGCGGCGACGAGCATGTCATAAACGACGAAGAAACAGAAACTCTAGTCTCTTCT

TCGAGGAGCTTCTCCACTGATTCTTCCTCGGAATTCAATCCCCACTTAGAAACAATACGTGAATCTCCATTTTCACGTAAAAAGAGAGCTAAGAAGGCTA

AAGGGCGTTGTGTTTTAAAGAATGGAGCGAAAGGAACAACGAGAAGAGGACGAAAGGAAAGAAATAGCCGCGATGGTTCTTTGTCGCCTGCAAGATTGTC

GAGGTTTCAGTGGCTGATACCGTGCACGGTGGAGGGGAAGGTAAGAGAGAGCTTTGCGGTGGTGAAGAGATCAGAGGACCCATATGAGGATTTTAAGAGA

TCAATGATGGAAATGATACTAGAAAAGGAAATGTTTGAAGAGAAAGATTTAGAGCAGTTGTTGCATTGTTTCTTGTCTTTGAATTCAAGGGAGCATCATG

GGGTTATTGTTCAAGCTTTTAGTGAGATTTGGGAGACTTTGTTTTGTAGAAGAAGATCTATTTCTTATAGGGTTTCAGCTGTTTAA

>PtOFP15 CDS sequence

ATGGCTAAACGTTTCAAGTTCAGATTCTCTCGATTGATCTCCTTTCAATCTTGCCGTTCCAAAGACCCCTCCCCTCTCCCATCAAATCCTGTTCCTTCAT

TTCTTAGACTCTCTCCGGTCAACCACAACTCCATCATTATCAACAACCTCCACCTCCCACCATCACAACCGCCTTCTTCCAAACCCCATCAACACTCCTC

CATCAAGCGCCATGTGTCCTCAGCTTTCACATCAATGGGCTGTGGATTTAGATCAAAATCCTCCACGCATTCCCTCTCTGAAACCGACCACGCCAAATCC

TCACAACAAACTGAAAATTTCCACTGGGCAGAGGAAGAAAAATATCACCTTGTGGCCAAACTCTTTGATGACGACTCAACTCCCCGCCGCAAACTTTACA

ACTCTTCGGCCTCTGAGGACTCTAAAAACCACGACGTTTTTCTCCCTCCGACTAACATAGAGAGGAAAAAACGACGTGTCAAAAAGAAGAAAAGGGCTTC

AAGAATCCGCATTAGCACTTCTTCAGCTGATAGCGGGTTATTTTTTACCGGCGACGAGAATGTCATAAACGTTGAAGAAACGGAAACTTTAGTTTCTTAT

TCAAGGAGCTTCTCCACTGATTCTCCCTCAGAATTCAATCCCCACTTGGAAACAATACGTGAATCTCCATTTACACGTAAAAAGAGAGGTAGGAAGGCGA

AAGGAGGTGTTTTAAAGAAAGGAACAACAAGAAGAGGACGAAAGGCAAGAAATAGCTGCGATGGTTCGTTGTCGCCTGCAAGACTGTCGAGGCTTCAGTG

GCTGATACCGTGCACGGTGGAGGGGAAGGTAAGAGAGAGCTTTGCAGTGGTGAAGAAATCAGAGGACCCATTTGAGGATTTTAAGAGGTCAATGATGGAA

ATGATATTAGAGAAGGAAATGTTTGAAGAGAAAGATTTGGAGCAGTTGCTGCACTGTTTCCTTTCTTTGAATTTAAGGGAGCATCATGGGGTTATTGTTC

AGGCTTTTAGTGAGATTTGGGATACTTTGTTTTGTAGAAGAAGAAGATCCATCTCTTCCAGGGTTTCAGCTGCTTAA

>PtOFP16 CDS sequence

ATGGCAGGCACAATAGGAAGAAACCTCAATCTATGCTTCACTAAGATCAGACGTCCACTACCACCCCATGATCAATCCCCCACTACCCTACTAACCCCAG

ATGATCACAGCCATACATTCCTCATAAAAAACTATAATTCCCTCTATGACCCCACCATTGATTCCGCCTCCTCCTCCACTTCTTCCAGCTCCTCCTCCTC

CTCTGAACCTGACTTCGCTACCGTCTGCGCCTCTCAGCGCTTCTTCTTCTCCTCCCCTGGCCGCTCCAACTCCATTATTGAATCCACACCGTCCATCGTC

ACTTCCTCAGACTCATCAGACAATCTAGTAGCCCCGCAATCTGACAGCAATGGTCTGACAACGAATCCCTCCAATGACAAGTCTTTGTTGGTTGATAGTT

GTAATAATAGTACTCATCCCCAGTTATTAAAATCCCCAACCGTTAAAGACAGTGTTGCTGTCCCCACCTACTCACCGGACCCGTACATGGACTTCCGGCG

ATCCATGCAAGAGATGGTGGAGGCACGTGACTTGGTGGACGTCAACGCTAATTGGGAGTATTTGCACGAGCTACTATCGTGTTATCTTGATCTTAATCCT

AAGAGTAGCCACAAGTTCATTGTTGGAGCTTTTGCTGATCTTCTTGTTAGTCTTTTGTCATCACAAATGCCAGAAGATGCTGGCCGCCGGGGAGAGGATT

TTTCCTCCGGTAGCTGTGGGATTTCGCGGCAGTGCATGTAA

>PtOFP17 CDS sequence

ATGGCCTTAATTAGAGTTAATTCTGAAAGGGACTTTTATCAAGATCGATCACCTCCTAGCTCCAGCCTCGATCCTAGCAGTATATTAAACCTTGCAAAAC

TCTATTTTTTCTGTTTTTTCTTCTCTTCAATGTTTTCAAAGAAGAAAAAGACCCTTCAAACCATACTCGCATCCAATGCAGGCTGTGGCTGTGGCAGGCC

AAAACTCTCAGATGTATACGAGCCGATACCAAAACCTAGACCTAGACCCTGCAGAACCTCAATTTCCCAAAAAGATCCAAACCCTAATTGTTCATCTTCA

AGCTCCTGTGACAAAAGTGTAGGCTTCTCATTGATGGACAATGAAGAAGAAGATTACACCTCAACCACTATCACTCTGAACAAAGACAACACTTCATCAT

CACAAAACTCCGAATCTGAAACCGATCCAAAAGCATCCAAGATTATTGACAGCATTGCTGTGGTGAAATATTCTAACGACCCATTTCAAGACTTCAAGCA

TTCCATGTTGCAAATGGTCGTGGAGAAAAATATCTACTCAAGAAATGATCTTGAAGAACTCCTCAACTGCTTCTTGGAGCTGAATTCTCCTTGCCACCAT

AGCGTCATCGTTCAAGCTTTCACTGAGATCTGGAATGAGATCATCTCGAAGAGGATCGTCAAGAAACCCTGTGCTCAGTTCATGTGA

>PtOFP18 CDS sequence

ATGAAACTCCCTTCAATCTTTAAGAAACAAGAAACCAATTTTGTTTCATGGAAATGGCCACCGTTCAGTATTGGGACATTTCCATTCCAATCTAAGGTGA

ATGCTTTAAAAAATTTCTACTTTGCTTTACGTGATGCAGCCAAGTTCATGACAATGCTGAATTCCTCATGGTCACAATCAAAGACTATCTCCTTGACGCC

CAAGGAATCTAAAGAGGACTCGCTGGAGATTGCTGTTAATAAGGCAGTGAGATCAGAGAGGTTGTTCTTCGAGCCAGGTAACACGAGTTCAATACTAGAT

GATCATAACGATGAAGCTAGCAAATTCCCATTCCCTGAATGTGTAGCACTAGCTATGGAATCTGAAGATCCTTATGAGGATTTTCGCAGTTCAATGGAGG

AAACTGTTGAGACTTGCGGATTAAAGAATTGGGAGGATGTAGAGGAGTTATTGGCATGGTATTTGAGAATGAATAGGCAGCAGCATCATTGCTTTATAAT

TGAGGCCTTCGTTGATCTATTTTCTGCTGCCCCACCGTCTTTTTTTTCTTGCCCTGTTTCTCACAGTGACTCTGCTTCATCTTCAAAATCAAAAGATTTG

TGGATGATTGAAGCTAAGAGGTCACAGCCAGCTATGGAAAAGGGGAAAAGTCTAAAGAATTGCTGA

>PtOFP19 CDS sequence

ATGCCCCCCATCTTCTGGAAGAACATTCTCAAATGCCTACCCACCATAATCCCATCCTCACATCCATTGCCCTCAGATCAGTTGCAGGAGTATAGGGATC

CATTACCATCATCCACTACCCTCATCTCCCCCACCACATCAATTATTATCCAGAACTTCAACTCCCCCTACGACCTCTCCTCAGCACCCACCTCCAAATC

CCTCAGTACTCCCTCCACCAACTCCTTCTCCTCCTCTTACTCCGACTCAGACACCGAATCAAATCTTGATTTTGCCACCATTCTCGCCTCCCAACGTCTC

TTCTTCTCCTCCCCTGGCCGCTCCAACTCCATCATTGAGTCCTTGCCAGAGCCCCAGACACCAGTCAGTGGAGGTGTTGCGATTAAAAAGTATTCACCAG

ATCCTTATACGGATTTTAAACATTCAATGCAAGAGATGATTGAAGCAAGAGAGCTAAGGGACGTTAGGGCTAAATGGGACTACTTGCATGAGTTGCTCTC

TTGTTATCTTAAATTAAACCCTAAACACACTCACAAGTTTATCATTAGTGCTTTTGCTGACATAGTAGTTTGCTTATTGTCTTCGCCCTCGCAGGAATCC

GACACACAACGGGAACCTGACGGCCTTCGTCGGTGA

>PtOFP20 CDS sequence

ATGAAGATCCCTGCACTGTTCAAGATCAAAGAAACAAAGCAATCATGGCAAAAATGGCCTTCATGCAAGCACCCCAAGACTCTTTCTTTCAGGGGTGGAG

ATGATGTGATCAAGACTGTAAATTCAGTTTTCTTTGATCCTTCTGAAAGGGTTGAGACACCAGAGTCATGGTTCACAAACTCATCAGAAACTACAAGTTT

CTCAACTGAGTCGGAGGGCTTTGATGGGGAGTCGTTGGAGGTTGTTGTACGTGGGGTGAGATCAGAAAGGTTGTTTTTTGAGCCTGGAGACACAAACTCA

ATACTAGAAGAGGCAAAAACAGGAGGGTTTCCATTCAAAGAAAGTGTGGTTCTAGCAATGGAGTCAGAGGATCCATATGTTGATTTTAGAAGGTCAATGG

AGGAGATGGTGGAGTCTCATGGACTTAAAGATTGGGATTGTTTAGAGGAGTTGTTGGGGTGGTATTTGAAGGTCAATGGGAAAAAGAATCATGGGTATAT

AGTTGGGGCATTCGTGGATCTACTTGTTGGGATTGCAGCTGCTTCTTGTTCTGATTCCACTTCCTTTTCTTCTGCTGTTTCTTCATTTTCTCCTTCATCC

CCTTTATGTTCATTGAAAGGGCAAAATGAGATTGATGAGGAATAA

>PtOFP21 CDS sequence

ATGCCCACCATCTTCTGGAAGAACATTCTCAAATGCTTACCCACCATAACCCCGTCCTCACATCCATTGCCCTCAGAGTTGCAAGAGCATAGTGATCCAT

TACTATCATCCGCTACCGCCGCCGCCCCCACCACATCAGTAATGATCAAGAACTTCAACTCCCTCTATGACCTCTCCTCAGCATCCACCTCCAAATCCCT

CAGTACTCCCTCCACCAACTCCTCCTCCTCATCTTACTCCGATCCTGACACCGACTCGACGCCTGATTTTGCCACCATTATCGCCTCACAACGTTTCTTC

TCCTCCTCCCCTGGCCGCTCCAATTCCATCATTGAGTCCATGCAAGAGCTTCATACCCCAGTCAGTGGAGGTGTTGCCATTAAAAAGTATTCACTAGATC

CTTACATAGATTTTAAAAACTCAATGCAAGAGATGATTGAAGCAAGAGAGATAAGGGACGTTAGGGCTAATTGGGACTACTTGCATGAGTTGATCTCTTG

CTATCTTAAATTAAACCCTAAAAACACCCACAAGTTTATCATTAGTGCTTTCGCTGATATAATTGTTTGCCTATTGTCTTCGCCCTCGCCGGAACCCGAC

ACCCACTGGAAACCTGAAGGCCTTCAACAACACAAGGTTTCACGTTTATTGGTGTGA

>PtOFP22 CDS sequence

ATGAAGATACCAACACTCTTCAGGGGCAAAGAAACAGAGCATACATGGCAAAAATGGCCTTCATGCAAGCACCCAAAGACTCTTTCTTTCAGGGCTGGAG

ATGATGTTATCAAGACTGTAAACTCAGTCTTCTTTGATCCTTCTGAAGGGGTTGAAACACCAGAGTCATGGTTCACTGACTCGTCAGAAACTACAAGCTT

CTCAACTGAGTCGGAGGACTATGACGGGGAGTCATTGGAGGTTGTTGTACGTGGGGTGAGATCAGAAAGGTTGTTTTTTGAGCCTGGTGACACAAACTCA

ATACTAGAAGAGGCAAAAACAGGAGGGTTTCCATTCAAAGAAAGTGTAGAGCTAGAAATGGAGTCTGAAGACCCATATGTTGATTTTAGAAGGTCAATGG

AGGAGATGGTGGAGTCTCATGGACTTAAAGATTGGGATTGTTTAGAGGAGTTATTGGGGTGGTATTTGAAGGTCAATGGGAAGAAGAATCATGGGTATAT

AGTTGGGGCATTTGTCGATCTACTTTGTGGGATTGCAGCTGCTCCTTGTTCTGATTCTACCTCTTCCTCTTCATCTCCTCTATGTCCATTGAAAGGGCAT

AATGAGATTGATGAGGAAGAACAGATGGTATAG

>PtOFP23 CDS sequence

ATGCACAGTTGGTGGAAAAGCGTGGCGGTTGCGAAGAAATCACAGGATCCATGCCGGGACTTCAAGAGGTCTATGTTGGAGATGATATTAGAAACGCAAA

TATTTGAGGCTGAGGATTTAGAGGAACTTTTGCAGTGGAGACAGAGTCATGGAGTTATTGTGCAGGCATTCTTGGAGATTTGGGAGTTCGTGTTACGTGA

TTCTCATGTAAAGAAAAAAACAATATTTTGGTGTTTTTAG

>PtOFP24 CDS sequence

ATGGATGCTGGAGAAGACTACAAAAGGAAGGCAATTACAAGGAAGAAGAGTTCTTACAGGATTAGCCTTTCGGCTAGTTTACCTGAAGATGTTTGTGGTG

CTTTCTCAGGTGATACTATCTGTGCAGTTAAGCTCTCTAAGGATCCATTCTCAGACATGAGAGCATCAATATTAGAGATGATTCAGAACGTGGGTGTTCA

TGATTGGGATGAAATGGAAGAGTTAGTCTATTGTTACATTGCCCTTAACTCTCCTGACCTGCATGGCATCATTGCCAATGCATTTCTTAGTTTATCTTGT

CATTTTTCCTAG

>PtOFP25 CDS sequence

ATGCCAAAGAAACTCCAAAAGTCTCTCCAAGACTATATCTATAAGATCAAAAACCCTACCCAAAATATCCAATTATCTTCTGATTCTTTCTCAAATTCAA

AGAACTGGATATTAAGAGGCTGCAAACACCCTAGAACACTATCCTTTGCCATAGCTGGTAACCAGAATAAGAGTCGTGATGAAGAAGACGAGGAGAAGGG

TGGCGCGGCAACACTCTCTGATGTGGATCGCTTCCTTTTCGAGAATTTTAGGTCACTTTATATCAATGATGACGATGGGAATTTTCAAAAGGAAAGTGAT

CGTAGATCTAGAGGAGGTGACCAAGCTCCGAGCATGAACGAGATTTTAATTGATTCACCTAGGTATATTGATCAACCACTGGACCTGTGTGGCTCCCATC

GATTCTTTGTGGAGCGGGGCTCATCATCCGGCTCACTTGTGGAAGAGGCACGGTCCAGCCTAACTGCCACCTCCGAGAACATGGGTTCAAGTTCAAGTTC

AAGTTCAACCTCAGTCTCAACCACCAGTACCCTCAATGACGACTCTGCCACAGTTGCTTCAAATGATCCAAAGCAGGTTAGGCTACCAGATGACTGCATC

GCAGTGCTAACCTACTCTCCGAGTCCCTACGATGATTTTCGGCGGTCCATGCAAGAAATGGTGGAAGAAAAATTGCAGAATAACGGTAAGGTTGATTGGG

ATTTCATGGAAGAGCTTTTGCTATGTTACTTGAACTTGAATGAGAAAACGTCCCACAAGTTCATACTCAGTGCTTTCGTGGACCTCATTGTTGGTTTGCG

CAAGAATCCGGACAAAGTTCCTGTCAGATCACGCCATTCTCGAATTGCAAGATCAGGGGGGAGGAGGAAATTGGAAAATGTAACGTAA

>PtOFP26 CDS sequence

ATGGCTGGCACAGCAGGAAGAAACCTCAATCTCTGCTTCATCAATAAGATCAAACGTCCACTACCACCTGATCATCAACCCCCCTCTAACCCACTAACCC

CAGATGATCACAGCCATCCATTCCTCTTTAAAAACTATAATTCCCTCTACGACCACACCATTGACTCCGCCTCCGCCTCCACCTCCACCTCCATCTCTTC

CAGCTCCTCCTCCTCTGAACCTGACTTCGCCAGCGTCTACGCCTCTCAGCGCTTCTTTTTCTCCTCCCCCGGCAGCTCCAACTCCATCATTGAATCTACA

CCGTCCATTGTCACTTCCACAGAATCATCGGACAATCTAGTAGCCCCGCAACCTGATAGCAATGGTCTGATAATAAATCACTCCACTGGCAAGTCTTTGT

TACTTGACGGTTGTAACAATAGCCATCCTTTACATGATCAACAACCACCCCAATTATTAAAATCACCAACCGTTAAAGACAGTATGGCTGTCTCCACCTA

CTCACACGACCCGTACATGGACTTCCGGCGATCCATGCAGGAGATGGTAGATGCACGCGACTTGGTGGATGTCAAGGCTAATTGGGAGTACTTGCACGAG

CTACTATCGAGTTATCTTTCTCTCAATCCAAAGAGTACCCACAAGTTCATTGTTGGTGCTTTTGCTGATCTTCTTGTTAGTCTTTTGTCAACGGAAATGA

CGGAAGATGGTGGCCGCCGGGAAGAGGATTTTTCTTCCGATGGTTGTGGGATTTCGCGGCAGTGCATATAA

>PtOFP27 CDS sequence

ATGTCCACTAACAAGAAAACGTTCCTTCTCAACACAGTCTCACTGAACTTAGGCTGTAGCAGCTGCAAGAAACCAAAACTTTCCAACATATGCCAGCCAA

AACCAAAACCAAAACCAAAACCAAAACTCCAAACCCCGACTTACCAAAAACACAAAAAAGATCTCTACTGCTCCTCTTCTTCAACCTCTTCATCAAAAAT

AACAACAAACCAATCTCCAAATGGCCATGAAAATCATGACACCCCGAACACATTTTCTCCAGCAATGGACACACCACCCCATTTCTTCTCTGACACAGGC

AACAACATGAAGTGTTCAACAGCTGTACGAGGATTCGGTCGCGTTGGAGGTGAAAGTGTTGCTGTTGAGAAAGATTCTGATGACCCTTATTTGGATTTTA

GACACTCAATGTTGCAGATGATATTGGAGAAGGAGATTTACTCGAAAGATGATCTTAGACAGCTTCTTGATTGTTTCTTGCAACTAAATTCGCCTTACTA

TCATGGGGTTATTATTAGAGCTTTCACTGAGATCTGGAATGGAGTCTTCTCTATGAGGACCGACACCACTAGTACAGGTTCCGAGAAGCAACTACATTAT

TACTATGGTTGCTAG

>PtOFP28 CDS sequence

ATGAAAATGAAAGCATTAGTTGTCTTCAGATCCAAGCTTTTCAGGCCATGCAAGAAACTACTAATACTCTTCAGATTCAAGCTCAAAGGACCTGTCTTTA

TAAGAGATCTTCGACTTCATCGTCGTAGCAAAAAACGCAGAAAAGCTCCTCAAAAGAGCCGAGTTTTTACTTTTTTTCGTTCTTTTAGGAAGTCAAGAAA

GATGGACAGAGTTGCAGAACTTAGGAGCGTCTCCGAAGCAGAGCGTGAGAGAATGCTCTATCCATCTCCTCTCACACCAGCTTATATCAAGGCCAGTTTG

GCCACAAAAAGGCAAACCTTTGGTGATGAAGATGTAGAAGATGCATGCAGAAGTTTCGAGAACTATTTGGTGGAGATGATGGTTGAAGAGGGAAAAGTAA

GGGATTTAGCGGATGTGGAAGAGCTTCTGTATTGCTGGAAAAACCTCAAGTGTCCTGTCTTCATTGGTTTGGTCTGCAGATTCTACGGAGAGCTATGCAA

GGACTTGTTCTCTCCTGATGTTGACAACACCGACGTTGATAGTCCCAAATCTCCCAAATGA

>PtOFP29 CDS sequence

ATGAAAGCATTAGCTGTCTTCAGATCCAAGCTTTTCAGTCCATGCAAGAAACTACTGTTACTCTTCAGATTCAAGCTCAAAAGACCTGTCTTTATAAGAG

GTCTTCAACTTCGTCGTCGCAGCAAGAAACCCAGAAAAGCTCCTCAAAAGAATCGAGTTTTTAATTCTTTACTCTCCGTTTTTCATCCTCTTAGGAAGTC

GAGAAAGATGGACAGAGTTTCCGAACTTAGGAGCGTCTCAGAACCGGAGTGTGAAAGAATGCTCTTTCCATCACCTCTTACACCAGCTTATATCAAGGCC

AGTTTGGAAAAGAAAAGGCAAACCTTCGGTGATGAAGATGTAGAAGATGCGTGCAGAAGTTTCGAGAACTATTTGGTAGAGATGATGGTTGAAGAGGGGC

AAGTGAGGGATTTAATGGACGTGGAAGAGCTATTGTATTGCTGGAAAAACCTGAAGTGTCCCGTCTTCATTGATTTGGTCGGTAGATTCTATGGAGAGCT

ATGCAAGGACTTGTTCTCTCCCGAGAGCTATGCAAGGACTTGTTCTCTCCCGATGACGACAACACTGACATAA

>PtOFP30 CDS sequence

ATGGAAAACCGATTTAAGACGCGAATCTCTCGCATGTTTCGTGGCTCATGTCGGACCCGAAACTTATCGGACGTGATTGAAAATGCTGTGTTTGTGCCTC

AAACCCATAAGAATTTCCACATGATCGAGCCTTTGCCGCCCAAGGTTCGACCTTTCCCTTCTATTTGCAGACACAAATGCCCTGAAGCAACAAACCAAGT

CCTCAATCACTCTATCATCTCCAGGCAAAAATTATCACACCGTTATCCTCCTCTTATAACTGCCAATACTAGTGGACACAGTTCTTGCCCTCCTGCTTAC

CCCATTTTTCCTTTGAATCCATTCTACAAAGACTTGAGTTTCAAAGAGAAAAAGAAGAGTTGTCGTTCAGTTAAAAACAGAAGCAAAAAGAAGAATATCA

TTAGCAAGAAAGAACAGACGAGTTTGTTCAGATCATCTTCACAAGATAGTACATATTTTGGAGGTAGCTATTATTGGTTTAGCAGCGAAGATGAGGACAA

GAGAGGGGATGAGTCGGACACTCTTTTCTCTTCAAGAAGTCTTTCTTCGGATTCATCAGGATCTCTTAGCCACCCTTCTCATGGAAAAAAGTTCACTTCT

CGGAGGAGAAGGGCCAAAGTGAAGAGTTCTCATGTGGGTGTTTTGCCATTAGATGGCAAAGTTAAGGACAGCTTTGCAGTGGTGAAGAGTTCTAGTGATC

CTTATAATGATTTTAGAACATCAATGGTTGAGATGATTGTTGAAAAGCAGATATTTGCGGCTAAGGATCTTGAACAGCTTCTGCAGTGTTTCTTGTCTTT

GAACTCTTATCATCATCATAGGATTATTGTTGAGGTTTTCATGGAGATTTGGGAGGTTTTGTTCTGTAATTGGTCTTGA
