# Supplementary material for: Identification, Expression, and Interaction Analysis of Ovate Family Proteins in Populus trichocarpa Reveals a Role of PtOFP1 Regulating Drought Stress Response
Source: Front Plant Sci. 2021 Apr 20;12:650109. doi: 10.3389/fpls.2021.650109 (PMC8095670; doi:10.3389/fpls.2021.650109)
Supplement: Supplementary Table 4 — The Protein sequences of PtOFPs. [file Table_4.docx]

**Table S4. The Protein sequences of PtOFPs.**

>PtOFP1

MGNNRFRLSDMMPNAWFYKLKEMGKTRNHNTTTHSIKKRQATSAAETQQPPSKPKHPQYNPYPRKSYYITRELISSEQIPHTSPRNSKSTYTNFPDPPRRSSNQRNRRRTIKASPKHVSAGCNCRATLWTKSDSPPDYSASLYDGSLDQETDFSDSFPPEFKSDSALATVSFDKMLSWSSSCDCKLDSIDNDGIVISVDKKSTARNLDNPKVFHSISDLDLPPIVTKPAKFDDQLEDTKKKETQEPTKYRRSPAKYEETNAHASLSVKVVKEESIAVKEYKTSSVRRNSVTSPGVRLRVNSPRISNKKIQAYNNGRKSVSSTTSSSSRSRRSLSDSLAVVKSSFDPQKDFRESMVEMIVENNIKASKDLEDLLACYLSLNSDEYHDLIIKVFKQIWFDLTDIKLNSCATTCESTYKLKAVYSAFITLPFPEKNTKEREQKLLIQKCLKLINNG*

>PtOFP2

MFRGSCRTRNLSDVAEKAVFVPQNHKNFHLIDHLPPKARPFPSICIRKCPEATNQAINPSIISRKNLSHRYPPASPIFPMNPFYKELGFQEKTKGRCSSIRNRSKKKKNITNKKDQMSLLSSSSQDSACFGGRYYWFSSEDENKREDDESDTLFSSRSLSSDSSGSLRHPSSRRRKYTSRRRRAKVKSSQVGGLPLDGNVKDSFAVVKSSSDPYNDFRKSMVEMIVEKQIFAAKDLEQLLQCYLSLNSYHHHGIIVEVFMEIWEALFSNWTAC*

>PtOFP3

MKLPFLSKINTNTDQSKRPLWPWPTYCHQPRTLSFSFRTSDGMFKTINSAFLDATNNDVVDSTPESWFTKSCESASFSTASD

DQSGAIDPIETVIRGLRSERLFFEPGETNSILEEAKAGDEFPFKETVVLSMESQDPYLDFKKSMEEMVEAHGLTDWEGLEELLSCYLKVNGESNHGYIVS

AFVDLLVGLAFASSSSSSSSITSTSTTQHHHDFCSSSHHSPSSPLSLYTSSTSDDDSSSTPCCVSSLENGADIISPCLTSLEAENGIKNINQ*

>PtOFP4

MEDIDWHFALSSQHSQGSAKAFDIDNPTIFSVPLATIFTHKYTEKEGEGKGRVVFLMGNNRFRLSDMMPNAWFYKLKDMGKTRNHNTTTHSTKKKQATSAAAAAVAESQQPPSKPKHPHYNSCPRKSYYNTRELISSDQKFHTSPRNSKSTDTLFPDPPRRSSKQRARKRTIKSSSPKLATSSVSAVCNCRATLWTKPNSPPDYSASLSDSSLDQETDFSDSFPPEFRSDCVLATDSFDKMLSWSSSNCDCKLDSNNYDDIVINMDEKYIARRSDDVDVFHKISDLDLPPIITKPPKFDDQVEDFKKKDTLEPVKYRRSSAKYEETNANASLSVKVVKEGSITAMKEHKTNTTVRRNSVTSPGVRLRVNSPRISNRKIQAYNNGRKSVSSTTSSLSRSRRSLSDSLAVVKSSFDPQKDFRESMMEMIVENNIKASKDLEDLLACYLSLNSDEYHDLIIKVFKQIWFDLSDIKLQ*

>PtOFP5

MSTNKKKHLFNTVSVNLGCSSCKKPKLSNIFQPKPKLQTPTYRKHKKDLYCSSSSTSSTKITTNHSRNDHEYHDTPSTFSPSMDTPPYFFSDTDNSGTCSRAVRGFGRVGGESVAVEKDSDDPYLDFRHSMLQMILEKQIYSKDDLRQLLDCFLQLNSPYYHGIIVPRSSCIITMAASHVTCENGSCCSARERLVGVQNHSKDDLRQLLDCFLQLNSPYYHGIIVRAFTEIWNGVSSVRSNTTTGSEKQLHYYYGC*

>PtOFP6

MKLPFLSKNNANTDHSSRALWPWPAYCQQPRTLSFSFRTSGGMLKTINPGFLDATNTDVVDSTSPESWFTNSCESASFSTASDDQSGAGESIETVIKGLRSERLFFKPGETNSILEEAKAGGEFPFKESVVLSMDSRDPYLDFKKSMEEMVEAHGLTDWEGLEELLSCYLKVNGKSNHGYIIGAFVDLLVGLAIASSSSSSSTITTASTTQHHHDSPSSPLSFYTSSASSDDSSSTPCCVSSLGNEVDIISPCLTSLEAENEIKKY*

>PtOFP7

MLNFFLVSPHLFFTFYNPNFVSSFYLFSNQREGKVNQLRQISERERKMGNYRFRLSDMIPNAWFYKLKDMSKSRKHYTSRASKKKPPPGTVTSQKPNISHQRYSYYFTTEPERAEKLDYYSPANPKASDTHFPDPPRKSSNRRNKRKTIYKPSPKLVSTFSADCSCRVTVNSNLTGFIPGDSPDCSSSPVESSYDELDFLSESDEDDGFLVPDSIDHHLASWSSSCNCNVSSSTTDIIIDMNEESYERKIKEVEGFGRIPELNLPPILTKPEKFNDNEVTKFRRSSSKLEEVKAHRSLSVKIVKEKSIRTYKEKRMNPPTRKSSVNSAKGIKLRANTPRIASRKIQGCSRKSVSLSRNKTLSESFAVVKFSVDPQRDFKDSMVEMIVENNIRGSKDLEDLLACYLSLNSKEYHYIIVKAFEQIWFDMTDLHL*

>PtOFP8

MGNYRFRLSDMIPNAWFYKLKDMSKGRKQYTSQAFKKKPPPGNVTSQKPNISHQRYSYCFTTEPGRAEKFHFNSPVNSKASDTHFPDLPRKSSNKRNKRKTIYKPSPKLVSTFSADCSCRVTVNSNLTKSIPGDSPDYSSSPAESSYDELDFLSESDEDDGFLVPDSIDHQLSSWSSSCNCNVSSSTADIIIDMNEESYERKIKEVEGFGRIPELELPRILTKPAKFNDKETEVTQFRRSSSKLEGVKAHRSLSVKIVNERSIRTRKEQKNNPPTRKSSANSTGIKLRANTPRIASRKIQTCARKGVSFSRNKTLSESFAVVMSSVDPQRDFKDSMVEMIVENNIQDSKDLEELLACYLSLNSKKYHDFIIKAFEQIWFDMTDLHL*

>PtOFP9

MKWGRKKTPSSSRPSLISHVFPTSWLTKFKHMSINPGQEHAKAKQKGKWNSVSASPLPFARGEGGGRFYGGDGDAFWRLSFGDESASTGALSSFHNDLDSELQAPPSSCHSCRSNATRVNNRKEDKIRFSNKVSEARKMRGLPREIEILPEMDACISEKVAEIRTPRLRVGREEKLRKTDQRVFEAQQFNLDGESYEAERVSRKETSKNISETESERTIGRIEREDCKLTASHSKKDFSTHLRKTKKDFVFAAQNESDGFSAENLSSEWQTLKDMKIEELKTKREKQRKSLYINRELQRKKKSKVRAISPRTASKVEICRIKALEDMKKAKMKKKKKAREKKMEGFTGLENFAVVKTSSDPQKDFRDSMIEMIEEKRISRSEELEELLACYLTLNADEYHDLIVKVFRQVWFDLNEACSDTELENEQGYDE*

>PtOFP10

MKESRVDKKQKLQQRGCKAFCCSCRLSVSSSEEAESSNPDRFASISSLAHAMVQERLDQMIRERQEARQRERRRRLRSDGTKFIVMVAMEKSSYDPREDFRESMVEMIMANRLQEPKDLRSLLNYYMSMNSEEYHGMILEVFHEVCTNLFLCCKCH*

>PtOFP11

MSSKKKNLLQSILTPNAGSGCGCGRPKLSDVYEPAPKPKPKTSISKKDPNPKHCSSTITSCDKSVGFSLPDSEEEGSTSTTFTLKKDNNTSSTQNSESETYPKASKITDSIAVVKDSDDPFQDFKNSMSQMILEKNIYSKDDLEELLNFFLELNSPCQHDVIVQAFTEIWKEIRCLSHES*

>PtOFP12

MENRFKTRISRMFRGSCRTRNLSDVIENAVFVPQTHKNFHMIEPLPPKVRPFPSICRHKCPEATNQVLNHSIISRQKLSHRYPPLITANTSGHSSCPPAYPIFPLNPFYKDLSFKEKKKSCRSVKNRSKKKNIISKKEQTSLFRSSSQDSTYFGGSYYWFSSEDEDKRGDESDTLFSSRSLSSDSSGSLSHPSHGKKFTSRRRRAKVKSSHVGVLPLDGKVKDSFAVVKSSSDPYNDFRTSMVEMIVEKQIFAAKDLEQLLQCFLSLNSYHHHRIIVEVFMEIWEVLFCNWS*

>PtOFP13

MPNRLKHKLSRVITPFQLCRSKDPSCPEAPIPAINRLSPFNPKALDINYPCNLQAPPPPSTPYYKCRVSRKTISVGCKCQSRSCPRCCMSDWSIESPDFAGKKEARWQAKPHLNVPFSFSDGSGDMSPFMVTGKNKNREINIKKNKVKTGVLSVDTSGCFSSTDVAGEENETLLCSSRSFSYDSSCEFSHSLDTIARQSEYHEAFNKPIGNKKVSNLKKIKKLGHQISLNKWKRSKTVTSPEIPSPVRSSVLKRVISRKVDGRVKESVAVVKKSQNPHRDFKRSMLEMILEKQIFEAEDLQELLQCFLSLNSRQYHGVIVQAFSEVWEIVFCDSPVNKRASIRN*

>PtOFP14

MAKRFRLKFSRVISFQSCRSKDPSTLPSNPVPSFLRLSPVNHNSIIINNLHLPPSQPPPSKPLHHSSIRRHVSSAFTSMGCGFRSKPSTHYLSETDHTKSSPPTENFHWEEEEKYHVVAKLFDDDSTPRRKIYNSSASEDSKNHDVFLPPSKIEKKKRRVKKKKTASRIRISTSSADSGIFFSGDEHVINDEETETLVSSSRSFSTDSSSEFNPHLETIRESPFSRKKRAKKAKGRCVLKNGAKGTTRRGRKERNSRDGSLSPARLSRFQWLIPCTVEGKVRESFAVVKRSEDPYEDFKRSMMEMILEKEMFEEKDLEQLLHCFLSLNSREHHGVIVQAFSEIWETLFCRRRSISYRVSAV*

>PtOFP15

MAKRFKFRFSRLISFQSCRSKDPSPLPSNPVPSFLRLSPVNHNSIIINNLHLPPSQPPSSKPHQHSSIKRHVSSAFTSMGCGFRSKSSTHSLSETDHAKSSQQTENFHWAEEEKYHLVAKLFDDDSTPRRKLYNSSASEDSKNHDVFLPPTNIERKKRRVKKKKRASRIRISTSSADSGLFFTGDENVINVEETETLVSYSRSFSTDSPSEFNPHLETIRESPFTRKKRGRKAKGGVLKKGTTRRGRKARNSCDGSLSPARLSRLQWLIPCTVEGKVRESFAVVKKSEDPFEDFKRSMMEMILEKEMFEEKDLEQLLHCFLSLNLREHHGVIVQAFSEIWDTLFCRRRRSISSRVSAA*

>PtOFP16

MAGTIGRNLNLCFTKIRRPLPPHDQSPTTLLTPDDHSHTFLIKNYNSLYDPTIDSASSSTSSSSSSSSEPDFATVCASQRFFFSSPGRSNSIIESTPSIVTSSDSSDNLVAPQSDSNGLTTNPSNDKSLLVDSCNNSTHPQLLKSPTVKDSVAVPTYSPDPYMDFRRSMQEMVEARDLVDVNANWEYLHELLSCYLDLNPKSSHKFIVGAFADLLVSLLSSQMPEDAGRRGEDFSSGSCGISRQCM*

>PtOFP17

MALIRVNSERDFYQDRSPPSSSLDPSSILNLAKLYFFCFFFSSMFSKKKKTLQTILASNAGCGCGRPKLSDVYEPIPKPRPRPCRTSISQKDPNPNCSSSSSCDKSVGFSLMDNEEEDYTSTTITLNKDNTSSSQNSESETDPKASKIIDSIAVVKYSNDPFQDFKHSMLQMVVEKNIYSRNDLEELLNCFLELNSPCHHSVIVQAFTEIWNEIISKRIVKKPCAQFM*

>PtOFP18

MKLPSIFKKQETNFVSWKWPPFSIGTFPFQSKVNALKNFYFALRDAAKFMTMLNSSWSQSKTISLTPKESKEDSLEIAVNKAVRSERLFFEPGNTSSILDDHNDEASKFPFPECVALAMESEDPYEDFRSSMEETVETCGLKNWEDVEELLAWYLRMNRQQHHCFIIEAFVDLFSAAPPSFFSCPVSHSDSASSSKSKDLWMIEAKRSQPAMEKGKSLKNC*

>PtOFP19

MPPIFWKNILKCLPTIIPSSHPLPSDQLQEYRDPLPSSTTLISPTTSIIIQNFNSPYDLSSAPTSKSLSTPSTNSFSSSYSDSDTESNLDFATILASQRLFFSSPGRSNSIIESLPEPQTPVSGGVAIKKYSPDPYTDFKHSMQEMIEARELRDVRAKWDYLHELLSCYLKLNPKHTHKFIISAFADIVVCLLSSPSQESDTQREPDGLRR*

>PtOFP20

MKIPALFKIKETKQSWQKWPSCKHPKTLSFRGGDDVIKTVNSVFFDPSERVETPESWFTNSSETTSFSTESEGFDGESLEVVVRGVRSERLFFEPGDTNSILEEAKTGGFPFKESVVLAMESEDPYVDFRRSMEEMVESHGLKDWDCLEELLGWYLKVNGKKNHGYIVGAFVDLLVGIAAASCSDSTSFSSAVSSFSPSSPLCSLKGQNEIDEE*

>PtOFP21

MPTIFWKNILKCLPTITPSSHPLPSELQEHSDPLLSSATAAAPTTSVMIKNFNSLYDLSSASTSKSLSTPSTNSSSSSYSDPDTDSTPDFATIIASQRFFSSSPGRSNSIIESMQELHTPVSGGVAIKKYSLDPYIDFKNSMQEMIEAREIRDVRANWDYLHELISCYLKLNPKNTHKFIISAFADIIVCLLSSPSPEPDTHWKPEGLQQHKVSRLLV*

>PtOFP22

MKIPTLFRGKETEHTWQKWPSCKHPKTLSFRAGDDVIKTVNSVFFDPSEGVETPESWFTDSSETTSFSTESEDYDGESLEVVVRGVRSERLFFEPGDTNSILEEAKTGGFPFKESVELEMESEDPYVDFRRSMEEMVESHGLKDWDCLEELLGWYLKVNGKKNHGYIVGAFVDLLCGIAAAPCSDSTSSSSSPLCPLKGHNEIDEEEQMV*

>PtOFP23

MHSWWKSVAVAKKSQDPCRDFKRSMLEMILETQIFEAEDLEELLQWRQSHGVIVQAFLEIWEFVLRDSHVKKKTIFWCF*

>PtOFP24

MDAGEDYKRKAITRKKSSYRISLSASLPEDVCGAFSGDTICAVKLSKDPFSDMRASILEMIQNVGVHDWDEMEELVYCYIALNSPDLHGIIANAFLSLSCHFS*

>PtOFP25

MPKKLQKSLQDYIYKIKNPTQNIQLSSDSFSNSKNWILRGCKHPRTLSFAIAGNQNKSRDEEDEEKGGAATLSDVDRFLFENFRSLYINDDDGNFQKESDRRSRGGDQAPSMNEILIDSPRYIDQPLDLCGSHRFFVERGSSSGSLVEEARSSLTATSENMGSSSSSSSTSVSTTSTLNDDSATVASNDPKQVRLPDDCIAVLTYSPSPYDDFRRSMQEMVEEKLQNNGKVDWDFMEELLLCYLNLNEKTSHKFILSAFVDLIVGLRKNPDKVPVRSRHSRIARSGGRRKLENVT*

>PtOFP26

MAGTAGRNLNLCFINKIKRPLPPDHQPPSNPLTPDDHSHPFLFKNYNSLYDHTIDSASASTSTSISSSSSSSEPDFASVYASQRFFFSSPGSSNSIIESTPSIVTSTESSDNLVAPQPDSNGLIINHSTGKSLLLDGCNNSHPLHDQQPPQLLKSPTVKDSMAVSTYSHDPYMDFRRSMQEMVDARDLVDVKANWEYLHELLSSYLSLNPKSTHKFIVGAFADLLVSLLSTEMTEDGGRREEDFSSDGCGISRQCI*

>PtOFP27

MSTNKKTFLLNTVSLNLGCSSCKKPKLSNICQPKPKPKPKPKLQTPTYQKHKKDLYCSSSSTSSSKITTNQSPNGHENHDTPNTFSPAMDTPPHFFSDTGNNMKCSTAVRGFGRVGGESVAVEKDSDDPYLDFRHSMLQMILEKEIYSKDDLRQLLDCFLQLNSPYYHGVIIRAFTEIWNGVFSMRTDTTSTGSEKQLHYYYGC*

>PtOFP28

MKMKALVVFRSKLFRPCKKLLILFRFKLKGPVFIRDLRLHRRSKKRRKAPQKSRVFTFFRSFRKSRKMDRVAELRSVSEAERERMLYPSPLTPAYIKASLATKRQTFGDEDVEDACRSFENYLVEMMVEEGKVRDLADVEELLYCWKNLKCPVFIGLVCRFYGELCKDLFSPDVDNTDVDSPKSPK*

>PtOFP29

MKALAVFRSKLFSPCKKLLLLFRFKLKRPVFIRGLQLRRRSKKPRKAPQKNRVFNSLLSVFHPLRKSRKMDRVSELRSVSEPECERMLFPSPLTPAYIKASLEKKRQTFGDEDVEDACRSFENYLVEMMVEEGQVRDLMDVEELLYCWKNLKCPVFIDLVGRFYGELCKDLFSPESYARTCSLPMTTTLT*

>PtOFP30

MENRFKTRISRMFRGSCRTRNLSDVIENAVFVPQTHKNFHMIEPLPPKVRPFPSICRHKCPEATNQVLNHSIISRQKLSHRYPPLITANTSGHSSCPPAYPIFPLNPFYKDLSFKEKKKSCRSVKNRSKKKNIISKKEQTSLFRSSSQDSTYFGGSYYWFSSEDEDKRGDESDTLFSSRSLSSDSSGSLSHPSHGKKFTSRRRRAKVKSSHVGVLPLDGKVKDSFAVVKSSSDPYNDFRTSMVEMIVEKQIFAAKDLEQLLQCFLSLNSYHHHRIIVEVFMEIWEVLFCNWS*
