# Supplementary material for: Identification, Expression, and Interaction Analysis of Ovate Family Proteins in Populus trichocarpa Reveals a Role of PtOFP1 Regulating Drought Stress Response
Source: Front Plant Sci. 2021 Apr 20;12:650109. doi: 10.3389/fpls.2021.650109 (PMC8095670; doi:10.3389/fpls.2021.650109)
Supplement: Supplementary Table 5 — The promoter sequences of PtOFP genes. [file Table_5.DOCX]

**Table S5. The promoter sequences of *PtOFP* genes**

>PtOFP1

GTAACAAAGGATCAAATACTAACCCGATACAGATTTGAATTTGTTTTTTTTTTTTTTTTTTTTGTAATATACCAGATAGATGTCACGTGATATGCCATGG

ACAAGGCTACATTCTTCCTAATATGAAAATAAAAAAGCTTAGAAAATAATAGCATTTTTAAAAGAGTACAAAAAATCTAAAACTTTGATCATTGATTCAA

TAATCTTAGATAATTTAATAATATGATTAAATAAAAAAAATAAAAAAGGCCAACAACTAATAAAAAAACACTTTCCATATTATGAAAAGATGCCCTCTAA

ATATTCTTAAAAAGTTTCAACGATTTTATTTAATAACAAATCAAAAATTAAATAAGAATGAAATAACCTCATAAAAAAATAAAAAAAATCTGAAGCTCAA

TTACCAGCAAAGTAAATGTTGAAAGACAAACTGAAAAAATATTAATTTTAAAAAATAACAAAAACACTTAACTCAAGTTAAAATCGTAAATTCACGGCCC

GCTCGTGAGACTAGAAAACCCCGCACATAGAAAATTAAATAAAACAATGAAAGTTAAATATTCTCATGCAAAGTAAATAATTAAAAAAATTAAAAAAAAT

CAATTAAAATTAACATAAAAAACTTGAGTAATCTCGATTAACCCACATCTCGACTAACCCACGACTCGAGATATGAGATCGAGATAACCTCACAAAAAAA

AGCGGAAAAAATCACGAAGCTCAAGGTCCAATAACCTAATGTTGAATAATAAAATTGAAAACAAAATCAATTTTAATAAAAAGGACATAAAAAAAAACTA

AAGTTAAACAAAACTTATGACTCAGGTCATTAGACCATTATTACCCCATAAAAGACAAAGACAAAAAAAAATCACGAAGAAAACTCCTAATTATCCAATT

TTTTTTTTAAAAAAACAAAGCAAGTAACAATCAAAACAATAACCAAATATGGTATAAAAATTAAATAAAAAAAATAATGAGGGACTAAATTGAAAAATAA

AATAAATCAAGAAAATGATAAAAAAAAACCTAAACAAAATAGCAATCAAAAGAATAAGGATCAAATTGGATAAAAAAATTAAATGAAATAAAATGTTGAT

GGATGAAATTGAAAAGAAATATCAACTTAAAAAGCATTAAAAGAAAAACATATAACAATCAAAAGAATGAAGACCAAAATTAATACAAATACAAACTTAC

ATAATACATTTAATATTTGAAAGAGTTGGCGTAAAATCTGAGGTAAAGAGAGAGAAAAGATGAAGAAAAAGTAAAAAACAAAGTCTACCAGAGCTCAACC

ATTGTTCCGTCGTGCACATGTGTCTTAACAACAGGAAAAGGATAACGCAACGTTGATGCAGAAGGTCATGTTTGATCGTCATGAGACGTTGCACGCATCG

CCAAAAAAACATTGACATCCCATTAAGTGATGCGTGCCACTTTTATTTTTTAATTACATTTATTCTTGTTAAAAAATTAATTACAATTAAAAAAACATGA

TGAAAATTAAAAAAAAATCGGTGGATATTAGTTTTATGTTTTTGCTTTCAAGAGTAGTATAATCATTTTATTGTGTAATCAAAACATTAAAATACTGAAA

AAATTCTGGACCTTAATTTTAAATTTTTTATTTTTAAAAATATTTAAGTCATTTAACTGTACATCTAAAATATTAAATGATTTTGTTGGAAAACGACACA

TGTAATTTTAGTTTTTATTTTTAATTTAATTTTAATAAGGCACTGAAAGTCATTAACCCTTGGGCCCCATTCCCATTTCTTTAGTGCCAACTTTTTCACC

ATTTATGGACCACAGCACCCTCTCTTCAATTCCAATCCGTCCCTCTTTATTTATCCTCTCCACACTCTCTGAGTTTCAGCCAAAGCAGAGGCATAGACAA

TCCAACAATTTCTTCTGTTCCACTAGCCACTATCTTCACTCACAAACACAGAAACACAGAAAAGAAAACGAGTTGGGAGAAGAGAAGGGAGGGGTTTTTC

>PtOFP2

AGTATAAAATAATATATATTATATTTTGGTTTAGCTAATTTAGATTTAGTCCATGGTTGGAATAGGTTAATTTAAAGATATTTATAACCATCAAATTAAA

AAATATAAAAATTAAATAAACAATGTCTTTTATTCTTAAAAAAAAAAAAACATGAACCGACAAATTGAAAGAATAGTTAGCTATGCATTCGTGATTCATT

ATCATCTTGCCTAATTCTCTGCTATTTGAATTTCAAATACCAAAAGATTCCTGCAAAAGTGCTCTCTTATTTTTTTTTGTCCCCTACCTACTCTCTCTCT

CTCTCTCTCTCTCTCTCTCTATACAATATATATATATATATAAGTAAGTTTGCATTCATGCTTTTTGTTACCTTGCTATAGTTATTTTTTTAAATATTTT

TTATATAAAAATATATTAAAATAATATATATTTTTTAAAAATTTATTTTTGATATCAGCACATTAAAAAGATCTGAAAACATAAAAAGAAATTAATTTAA

AATAAAAAAATTTTAATTTTTAATTTTTTTAAAATACTTTTAAAACGCAAAAACATGGTCTAATTAGGTTGTTCATGACCGCTGCTTTATTAGTACCTGT

TTTTTTAATTTTAATGACAAATGATTTCCGTTTACTATTAAAAAAGTTAGTCAATAGACACTTTTCAATTAAAAAAAAATTTAACTTGGTTTTAAGAAAA

AAAATATTTTTTAATTTTGGACAAAAAATACTTTCTAGAAGTTTTAAAAAAGTAAAAAAATATATCTAATTATTTGTTAATTATATCAAATTTTATCTTT

AATATATTGATTGCTATATATTTTGTTTCAAATTTGTTTTCTTTAATTTTATCCTTGGAATTTGATTTAGTTTGATTTTCATACCAAGTTGATTCTTTTT

TTTTATTGTTATTTATTTTTCTCTTATCCTTTTCTTAATTTGTATTATTTATCTATCAAGTTTGATCTCTATTTTTTTAATTGCTATTTATTTTATTTTA

AATAATTTATGAAATCAAATTATTTTTTTCAATTTCATTCTCTTTTAATTTATCTATCTATTAGATTTGGTCTCAATTCTTTTGATTACTACTTGTTTTA

TTTGAAATAATTTATAAAATTAGAAATTATTTTTCAATTTTATCATTCTTTAACTTTTTTATCTGTCAAATTTAGTCTATTGTTTCTAATAAATTTGAGA

AAAAAATTAAAAAATTATTTTCCACCTTATTTTTTATAATATAGCCAAACATTAAATAATATTTTCTAACTCATTTTTCATAACACTATTAAATAAAAAA

ATAATATACTTTTAAGAAATTTATTTTCTATAAAAATTATTTTCTAAAAAAATTTATTTTTCGGCAAATAAACAACACCTAAGTGTTTAAAGGAGGTTTT

TATTTTTCTTTCAAATGGTGGATTTAATAATTATAAAGATTCACTTATTATCTTAATTTCATAAGGAAATATTATTATTAAAAAAATCTATTCTCCTAAA

TATATTATGATGTGAAAGATAAATTCTTAAAATTAATGTACAAGAAAAAAAATTAGAATTCAATCATAAAAATACTTTTTATGGTGAGTTATAAAAAAAA

AGTGCACCTAGAATGGCTTTTAAACGTTCCAAAGGAAGTCCAATTCAAAATAAAGTGTTGATGGAGTAATTTATGTATAATATAATAATTTATTAATCAT

GGATGATGAAAAAAAAATGTAATAATTAAATAATGATTGATTTGAGGTCGTCTCCTCTATCAATTTTTTTCCATGTCCAAGAAAGAAACGACGAAAAGTA

TGATGATTCATTTATGAGTACGCTAAAACCCTTAATATAATAATAAACTTTCATTTTCGGTTTCTCATGTATTTATATCTCAACCATCCTTATACCACCA

CTCCCTTCATAAACCTATATCCCTCTCCCTCTCTCTGCTTCTTCTGCAATATTTCTCTCTTTCTCTAATGGAAAACCGATTCAGGTTGCGAATCTCTAGC

>PtOFP3

AATCAATAGGTATGAATTTTAATAAGATAATGGTTACATTGCATTCAAAATTCAAGGGCTAAAGAATAATCTACTGAAAAACCAGTTGATGCAAAAACCA

AGAAACAAGTTGGGTTCTCTCTGTCGGAGGCGCCATATACTGAGAACACAAAAGCTCGCCAATCTATCAAATGGGCGAAGAAACAAAATAACTCTGTCAC

TCTCAAGTCTCAATCTATTCCCTGGAACGCATATGGAGGGCACGTGAAATATTAGTGTTATTTGTTACAGCACTTGCAAACGATTTTAATCCCTACGAGC

TCCGTATGTTAATTGTTTTTGTTTTGTACTGCCTTTTCTTTTCTTTCAACTTAATTTTGCTTCCCTTCGTACTTAAATTTCTTCTTTCGAGTTATTTTAG

TTAACATAATTACACACTCGAGAATCAAATAGTTTTTTAAAAAAATTATAAAAGAAGGTTTGAAAATAGAATACACATTATATTCACAGAATCAATCGAA

CTACAAATGACTTCTAGAAAGAAATATATTTAGAAACTACTTTCACTATCAAAAAAGTTGTCTAATTGCAATGGAATTATCAAACGAAAGTTTTTTGTTT

GTACTTTCCGATGCTAATAACGATAAAATATTTCTGTCAAAAATTTATGATGTAATTACTGACAGATCTAAAATTCTTCACAAGACACTAGCTTACAAAA

TATATTTTTTTAGTAAATTGATCGGTGATAAGCACAACTTTAACATCGACGAAAAATTTGGTTTATGCAAATCAATTTTCTAGTGATGTTCAAGGGATCG

TTATAAGCAAATGCATATCAAATATTTAGAAAATTATTCAATGAAGTATGTATGTCTGATGTTTCTCTCTTCTTTTACACTTAATTTCATGTGAAGTTCA

TTGTAAAAAAAAAAAAACAAAAAAAAGGGGGTTTGAATTCGATATATTTTATAAAAAGATGCAATGCTAATTTTAAGGGTAATCACACCTTCTTGCAATT

ATCCCATTTTGATTATTTGGGTTCCACGCACGATAACAAGGTCTCTAATGCATATGGGCAACGAGGCAGGTACTACAAACATGGCTACACAGAGCTGTTC

TCTAAAAATTACTATCTATAAAAAAAATTCCTCACGAATATGAGAGAAAACGACCGCACTGTACGAAGTAAATAAATTGTCCCTCTATTTCATGTAAACA

GCTGGAGTTAATTTCTTGACACTGCGTCAATGTGAAGGTTGTATGCTGAACCAGCTAGTCGAAAGCCATTGGTTTCGGCGTCAACATTGGGACGGATATG

ATATTGTTCTAGCTTATTAGTTTCTGCAATCTATGGTTTTGGTTTGAGGAGTTCATGATTTCTTCTTGTGGAATTGATAATTAAGATTCCCTCTTAATTA

ATAATTAACTACCCGCATAGGGAATAAATGGAAATTTATACTGCTCACCAGCAATTATGAACTTAATGATTGATTGTCAAAAGCTTATTAGATCATTTGA

AAAGTTTCAACACTCAAATATATTCAAAATCTTTCACAAGAAAACAGCAAAGCTTAGCTAGGTCACGTAATTGAACTACAAAGGCTGGAAAGACAGGACG

ATTGTGTAAAAGGAAAAGAGATATGGTAGTAGATAACTCTATTTAATGAGAAAAGGTGCCATAATTTTCAATGGCTTCAACTGAAAGACATTTACTTCAA

AAGCTCAAAATTTAATACACAACCAAGAAAATGTACATGTCCCCACCAGCCTCCCCTACTCGGCTAGTCCCCTTTCCCTCCCTCCCTCTCCCTCCTCTCT

GCATTTCTTATTCACCACTTGTTTAAAATCTGCTCTCATAACCCAGCTGCCTCTATGAATTACAAACCCATAAAGAAGCTCTACAAAGTTTTTCATTTCT

CACCGCCGCCAAAGATCATTTAAACCAAACTTACATTTCTCTCTTAAAGCACATCGATCTTAGGCAAAAATTTAAAACAAACCAGCTGATGGGCAAGAAA

>PtOFP4

ATATTTTATTTTATTTGGGATACATTTCACTAACAAGATAGAAAAAAGTTGATTTTTTTCTTTGCACTAAAATTGCAACTACTTTCAGTTCTCAGAATGA

TTTACTTGAGATCAATTCCGCAACTATCTCATTTTATTAGGTTTGTTTATTTTGTGTATTTTCTAGATTTTTTCTTGTTTGTTTTAGAAAAAATATTTTT

CAGAAATATATTTTTATGTTTGTTTCCTCTAAAATATTTTACTAGAAAATTAGTTAATGAAAAAGTTTGAGATTTGAGTTTATTGTAAAATGATATTTTA

GACTATAAAAAATTATTTTTTGCCAATTTAAATTAGAGCTTTGATCTACTCCTTGAAGAATTATTTCTTCCAACCCACAGCACTATCTAGTCCAGCCCAT

GAATAGTAGAACTAATCAATTTCTATCAAGAAGGATTTTCTACAAGCTTGAATAATTTTCTCAACTCAAAATACATATGATTTAAAGAAAAAAAACGTTA

AGGTTTAATTCTAATTTTATGGAGGGTAATCAAAGTTGATAATAGAGAAATTTCATCAAGTTAAGTTCTACAAAAGGGGTTGAAGAAGAATGTTGTGTGG

TTGATTTTTTGCACTGTTTGGATAGATTTTTTCTGCAGAATTTGACACTCTTCTTTATCTATTTTTCCATCTTAACTCTTATGGTTTTTTAAAGCTCTAT

TCCCCCCCCCCTCATAATCTAAAGCTTAGGTTCAGCCTTTATAGAGAGACATTGAATATAATTTTTTTGAGTTTGTTGGACCTTATTTAAAAGTGTACAT

AATCAAGGAGGAGGAGTTATTGAAATCTTAGGAAATTTATTAAAGCTAATCCTTTTACACCAACTAAAAAATAATAAAACTTTAAAAACAAATGTTTGAT

TCTTGACAACAATATTAAATTTCAGATTGATTATATACTAAGTTCTTCAATAAGTATTTTTTATTTTTTTTATTTAAGTATATTTTCAATATATTGTTAG

AATGTATGCTATTAGTTAATTTTATTTTCTTATGCATTATTTTAATTAATATGCATGTTGTGATTGCATTTTAATTTTTTATGCAAGTTTATATATTTTA

TAGTAATGATACCATGATCTTATATATTAAATGATTATAAACTAAATATATGTATGCTCACTTTTCTATGTGACGTTTAATAATAATAATAATAATAATA

ATAAGATAGATCAATTGCAGCTCTCCAAGGTTAAATTTTAAGAATAAAAAAAGATGAAGAGATAATTTATTTGTGCAAAAATGCATGCAACAGTTTCTTT

ATTGAATTAAATTATATATAGTTAAAATTCCATCGAATTATAATGCCAAAAATATTCAAGTACATGAACTTTATTGCGTTATATGGTCGGGCTATGTATG

TATAATGAAGAAAGTAAATTGCTGGGAACGCAAGTTTTGTTAGAAGTCAAAGTCATTGAAAATCAAGAATATTATAATTATATAAAAAAAATTCAAAATA

CTTATTTTTATTCATTTAAAATGATTTTTTTTGGTACATTAAAAAGAAAAGAATAACTAACACATGTTTTTCATCAATGTGTATTTGACATATTTGATAT

TATAATGAAGAGTATGTATATATATATTTTTTGGTAACTCGGGGTATCTGGGCTAGCTTACGCGCACCACAACTAATCCCCGGATCCACTGAACACCCTG

CAAGCCCAGCAGGCAGGTAAGACACCGCGGGGGTGACAGGCGTGCACGCTGAGGCTCGAACCCAGGTGACAGAGGCAAGAAAACCTTACCTCTGCTGTTGGGCCACAAGCCTCGGTACAAGAGTATGTATATTTAATATTATGAGAGAGTATTTTACAAAAATACTTTTTAATTGATATATATATATATCAACACATTAA

AATTATCGGAAACAAACACTCGACACACTGCTCAACCTATCATGAACTCCGTGTGGGGGTAATTTGCATGCCAGCAAATCGGACAAAAAAGCTGAAAATC

>PtOFP5

AAAAAATAAATTTTTTTAAAAAAGTCAAAATGTATCTAATTTAAATTTTGATTAAATTGCAGATTGATAATGAGATCGTATATGTTTGAATTGATCAATA

TCCATTCAGTTTGATATAAAATTAAGACTGAGCGAAGTCTTGAGTTACTAAATTAATATGCGGTGCGAAGCTAAATTTAATAGCATTGATAAAATCCGAG

GGGTCTATATATTTTTTTAATCAAAAGAAATTTATAGTATTCAAACAAAAAAAATTAAATTCGGGACTAAGTTCAATTTGACTCAAAATTCAGGATAATT

TTGAATAATTTAAGGGTTTTTTTTATTATTATTCATTTAATGAACAATAGACTAAAGTCCGTGCCATTATAGCTTTACTTTAATGAAAATCTTGAATGAT

TCCGAATCTTTCATAATCATAGCGTCATATATTTCTTGAATTTGGGAGATTCGCTACTTTCTCATAGACATTAAACCGGATCTTTTAAGCATTCAATGGT

TACATGGTTGAAGATTTACCAGAGTGTTAAAGCTGGAAAGCAGCTTTTTCTACAGATTGGTGCTGGATATTGCTGCTTTTATAAGGGTAGAGAGGAAAAT

GCAAGGATCATGTGTTCTTCTATTAAAATAATAACTTTCTATATTTTACTAAATGAAAAGTTGTTTTTCACCATTCCCTACATTAAACTAGAGGAGTTTA

CTTGGAAATGAAGAACACGTTCGAGTTCTTCGTAGGTTTCAGTGTATGTTATCTTGTTTCGGTTGCCGAAGAAGCAGTGTCAATGACAAAGATTAGTTTA

GATTTGCCGTTTTTATTCACTAATCCTCTTATCATAATCAAAACTTGATTACGTTTTCAATACTAAAATTCAAAAATTTACCATCTTAATTAAGTGTCTT

CTATCGAAGTTCGTTATGTTTTAAACATGTAAATGCCAGGAGTTAGAAGTTTAGCATTAGATGTGTGTTTGATCTAAGGAATTGTTTGGGATTGAAATGG

TTGTTTTTTAAAGTATTTTTTAATTTAAAATATATTAAAACAATTTTAATTAAAAACTAATTTAAAATAATAAAAAAAATTAATCCAACCGCAACTCCAA

AACGATAGCTCGAAGATTGATGTGGCTAATAGTATGATTGTGGGTATAATTGTAAATGCAGTTGTAACTGTAACTAGAGAAAAAAAATTGCTTGAAATGT

AAGATATATGTATAAAATCATTTTAAAAAAATAGAGTGTAAGTAAAATTTAGAAGTTGAAAGTTGAAAATTTTAACCTTTGATTAAGGTTTTGTTTGATA

TGGTTTTTGTAAATTGATTTAAATTTCTTACCTATATAAAAATCATAAATAAGTTTGGTTAGAATTAATTATTAGCCTAAATAATAAAAAAAAACTTATT

ACAAAATTGAGTTAAAATTCTAATTTAATAAAAAATAAGATTTTTAACTTATGGTTTATGGATTCTAACTTTTATATATATTCTTCAAAAGAGTTGTTGT

ATTAAACATACTATCGTTTTAATATATTTTTGTTATCGGTTATTTATGAAAGAATAAATTGAGATTAATTAACATGATTCCTAGCTTTACTATGATGAAT

CAGTTCCCTTACTTTCGAAGCCTTACCTGTATGGGAAAAAGGTCAATAATCATCGTCAAGCATCTATCTTGTAATTATTTATGTGAGAATCTATCTTCTT

TATAGTTGTCCTCAAATCATGCTATGGTACCTACCTGGATTAAACTAGGAAGAAGAAAACAACTGTGACGTTACAGTATATAACATGACAGCTTACCAAT

GGGCCTTTGGAGCCATGTCTAACAGTTGATTTACACCATTCCACCATGGCAAATTTCCTGATCGAGAATAACTCGTGTACGGTCAAGATCATGCTGTGAT

ACATGAATACCAACAGACGAGTCGTAATATCATAATAGAGAGGGGGCCCATCATAAGGCATGCAGGATTTATAGAGAACCCTCCCTTCCCCTCCCTCCCT

>PtOFP6

AGATGGTACATACACCTTAAGGATAGGTTCTATTTATTAAGTGAGCATGAGTAGGTTTTCTATATGATCTCAAAAGTGTCTCAAATCTGCCTAGTGATAG

TCCTCGTAAGGACAATATGGGGTCATTGCAAGAAACAGTTAAGGCATGTATGCCTACCATTACTAAACAAGCACTCAGATCTGAGTTTGGTGTTTTACGA

GCCACAACCTTTACAAATAATCATATGGTAAACCATTAATCCCTCCACCTAACCAAAAAACATGTGTGCTCTAAAAAATTAAAGCATGTGATGCATGAAA

GCATTAATCCATAACTAATATACAATGCATGAGCTTTGTATATAAAATGCATGTTGACAATAAATGCATAGACCAACCAAACTAAAACACAACAATCAGA

CATACAACAAATCTTAGGCAACAAGTAAGTCCCAAGTGGAGTCTCTATTTTGTCGCACTTGTATGATATTGCAATAATCATCCTTCTGTATCAAGTTTTG

GGTGTATATTTTTGTTGGATTCGCATAATTTATCATCATTTAAACCAACAAGTTGAAGCTCAGATGACTTATCTTGCTGCTAAAACATAACAACTTAATG

TTGAGATTATCGAACTCTTCCGATTATACATAGAGTTAAAATCACAGATAAGTGGTACATGTGCTCCCTCTTATTGGCCCCACGGTCTCTCCTCCTACTC

ATGCTCAAATGAATTATATTTGAACTGATAAATTTTTAAATTTGTAATGAATATTTTTATTTTATTTTAATTATTTTCCACTCTATTTAATAGATTTTAA

AAATATATATATTTTTAAAACATTAGTGATGCTCTTACCGACAAATTAAGTTCATTAGTATATTCCGGAGAGTTGGAAAAGAATTATTGCAAATGTCAGT

GTCACTTTTTAATCACCAACTGTTTAATTCCGTTAGTAATATTAAAAAAATCATCGATGGAATTACATATAGTCTTTTTGTCGATGATATGCCATATTCA

CATAGGGATATACTGACGAAATGAATCGAGTAAATTTTTTGTTTAACGCGCTTATTTCATCTATAAATCCATCTATAATTATTTTACCAATGGAATTACA

GATTAAACAGGAATCATCGCGAAAAGTTTTTTTAATGAACAGTAATTATCTGTTAGCCTGTCGGTAAAAGTCGTCCTGACAGACTAAGAGGGTGCTTGGC

AGTGTGGTAGTGGTTGCTTTTTAGATAACTTTTTGTGCCGAAATGCATGCCAATGATTTTTATTTTATTTTTTAAAACTTATTTTTGACATCAGCACATC

AAAACGATCCAAAACGTACAAATCATATTAAATTTTAGCAAAAACAAATTAAAATTTTTTGGGAATGCGGTTTGCACCGCGTTCCCAAACATGTTGTAAG

TGTCTAACTACCGATAAAATATTCAGTAGTAAATTTAAAATTCTGGTAGTGTAATTAATTTAATGATTTATTCTACCCCTCAGCAATTATCATATACATA

CTTGTGGTAATTTATTAGACTATTTGAAAACTTTCAACATTCAAATATATTCAAGTTTTTACACAAGAAAACAACAAAGCTTAGCTAGGTCACGTAATTG

AACTACAAGAACCTAGAAAAGGAAGAGTTTGTGACAGAGAAATATAGCAGTAGATAACTCTATTTAATAATAATAATAATAAAAAAAAAAGTACGATAAT

ATTCAATGACTTCAACTGAACGACATTTACTTCAAAAGCCCAAAAATTTAATACACAAAAAAGAAAGTGTGATAGTCCCCACCAGCCTCTCCAACCAGCT

CCCCTTTCCCTCCTTCTCTACTCTCCTCTCTGCATTTATTCTCCTTAATTATTCATGACCACTTCTTCAAAATCTGCTCTCATAATCCTGCCTCTCTGAA

TTATAAACCCATAAAGAACCTTTCAAAAAATTTACATTCTCACCGCCAAAACTCTTTTGAACCAACTTATAACATTTCCCTCTTAAGCAATACATTTTAG

>PtOFP7

ACGAAAAAAAGTAAAAACTCGGCTCTTTTGAGGAGCTTTTCTGCGTTTTTTGCTACGACGATGAAGTCGAAGATCTCTTATAAAGACAGGTCCTTTGAGC

TTGAATCTGAAGAGTATTAGTAGTTTCTTGCATGGCCTGAAAAGCTTGGATCTGAAGACAACTAATGCTTTCATTTTCATCTGTAATGATTGATATCAGA

GTGTTGTTGAAGAAATGGAGCAGAATGGAGTCCTTTTATTGCTTCTAGCTAGTTCAAGGACATAATGTTGCTCCACTAACGGTTGGTGTACCAGAGGGAG

AGAGAGTTTGTTGAAGATGGATATAATAAGAGGTTAGTTAAGCCCTGTGTGCATCTCCTTGCTATTGTAGGGTTCATGTCCTCAAGGAGGAAAGTGGCAC

CCCATGAAGTGAGAGACTATTTTGGCAAGAATCCTGATATGGAGAAGAGTTCAATGGATGCTCATCATTTGGGCCAAAAGTTCCATTAAATTAAAAATGA

TCCTCATTTTCCAATTTGTGAATAATCTACCAACTTCATATGTGGCCTTCAGATTGCTAGCAAAGCCATGTAGTGGAAGCCATGTTTACCAAATATATAA

CCGGCAAAAACCTCTCGCTTATTGGCGCGAGTGATGCATGGGTTAGTAGCTTTTTCTTCTCTTTTATTTTTATAAAAATACTAATTTACTCCTAATCGAA

ATAAAGTATAACAAAAAGACCTAGAGAAAAATACTAAAATAATTTTTAATTCATCCTTTATTTATTTTTTTTACTTTAAGGACAATGAAGTAATTGTAAA

CAGTGCAAAAATAAAATTACGCACCTTTCATTTTATTTTATTTTATTGCCTTAAGGGTATCTATATAATTTAACTGTGAAAAAAAATATAAACTACTAAA

TTACCCCTAATTAATTTGCAAGTGACCTTTGAAATCCGTGAAAAATATCATTCTACCCCACCCACTAGCAAGGCAAAGACTTTGTATAGGAAAGGATAAA

ATAATAATTACTGTGGTTAGTAAAGCTAAAGTCCAAGACAACCTTGTCCTTGGTGGCTGGTGCTCTCTTTGGGCTAAAATCTAGCTCCTAAAGCCCCAAA

ACCATACACATTGAGACTCCAATTGCTTCTCACAAAAGCCATCCTCAATCTGGGCTGTGCTTCTTCTGGTTTCATTCACAGTCTAATTCTAGTATAGCCA

TGAAACCAACTTTTCCATCTAGTCTTGGCCTCTGAGTCCAGAAAAGTTGTTGGACACTAGCTTTATAGTTGCTGCTCTTACAATGGATGTCGAGGTGGCA

GCGCAATCAGTAACTCTTGCCAAAAAACCATTTGTTGAACGCTTGAATGCCATTAACTTCCAGTCCCCGGTAAAGAGAGAATGTCTTATATATATCAGAA

TAAGAAATTTCATACTTGACGAAGTTTTTTTTTTTCTTAAGGAATTTTTCAGAAGGTTGATGAAATAATTGTTGTGATCCTTAACATACATGATTCCTAA

ATTATTGCTAACTGTATTTGGTCCACAGTAGCACAGCCTAGTTCCTAAACTCAGTAGCCAGAATTAGGTGTGCCTCAGACAAAACCAGCATGTGATTGAC

CTCCCTTCACCTGTTGCTGTATCGGAATCCATCAGTTTGGAGAAATGCAATTTCATCCTCAGCTGGGAAGAGAGAAGTGAAAAAACCTATAAAGTGTTTG

GTAGGGGACCCGAAACTGAATGTGCAGCACCAGGCATGTGGGGCATTGTGAAATCAAGTTGAAAAGGCACGGAATATCATCATTTGTGAAACATGGAATC

ATGGGAAAGAACACAGCACTTAGTAGACATATGGGCCTTGATTAATGCTAAAATCATCATTACTCTCTCTTAACCCTTGATTTGGCTGAGCAAAAGATGA

GGCCCCAGATGAGGTGTTCTCACAAATCTTCATGTTCCAGCGATATATTGGCTACTGGCCACAGGAGTGTATATAAAATGGTACAAGTGATTCACAGACT

>PtOFP8

GGTTGTTGGAATTGGTTTTGTAAAACATGTAATAGATTTTTTAAGTTAAAATTAAATGTGAGATACACTTATTTTCTTGAGGTTAAAATAAGTTCAACAT

GACTTTCTAATTCAACATGAGTATATATTAACATGTATTTTTTTAAAAAATAAAACAAATTAAAAATTATTTAAAAAAATAAAACAAATTAAAAATTAAT

GAAAAAATGTTCTTAAAAAACTATTTATATAATAAAAATTTCAAATCCAAAATAATTTTGTGAATTGGATAACAAGCATATGGGTTCATGAGTTTAGATT

TAGATTTGGTTATGAAAAATATATTGCTAGTTCCTTTTTTTTTTATCAGTAATGTCTTTTAATTATTAAAATATTTTAGTAAATGAATCAATGCATTAAT

TATATTATATGATAGGAATTAATGTATTCATGAATTATGAAGATATTTTTTGTTAGAAAAACAAATACAAAGATATAAGAATAATATATTTCTAGGATAT

TTTTGATGTGAATTTCTATCATTCCCAAACATTAGGGTTTTCTCTTTTATAATTATCTTCATTTTTTTTATCCGAAGATTGTAACCAAATACTAACTAGA

TCACCTGAGCAATTAGCAACTCTTTACGACATAATAAATTAGTTGAATGCCAAGAACTTCCATATTCCTTGTTAATAGACTGCTTCTTTTCTCTCTTTTT

TTTTTAATTTTTTTTCTCAGAATATTAAACTTCATATTTTGTCAAGTTTTTCTCTTTATGAAATGTGGTCTAATTTAATTCTCTAGTATAAATAATTAAC

TAAGTTTGTTTTCTCAATTCTGTGATTTCACTCTTCACATCAACATTGGTTGTTAGGAACTTGCTTAAGGTCCCAGCAATTTTTCAGAAGATGGATGGCA

TAGTTGTTGTGATCCGTACATGATTTCTAAATTATGGAAATAAGATTCAGCTCTAGATTACCTATCTAATTCTTGATTACAAATTATTGCCAACTATTTT

TTTTTTTTGGTTTCTGTAGCACAGCCTAGCTATTGATTACAACGCAACTTAGAGATTACGCTCTGCTCAACAACGCAACACAGAAATTGTGTTATTTCTC

TATATTTTTTTGGCTTGCGCTAGTTTGCCAAATATTGCTTTTCGGCATGTGTTTGTGAAATAAAAAGAAACTCAAATTAATCTCTTTTCTTTTAACATGA

CCGGTGATTACCTCCCTTGGCTGTGTCCGATTCAGAATCCATCATTGATTACAACCATGGATGAGCATGAACCTCGCATGAATGTGAGTGGACAGACATT

TACTATCGAATAATTGCTCCACAGAGATGTTATCAATGAATTCGGCTAATTTACAGAGCCATTTCCCCCCCTGAAGTTTGGAGAAATGCAGTTTCATCTT

CACCTGGGAAACACAAGTGAAAGAACCTGTAAAGTGCTTGGTAGGGGACCAGAAACAGAAGGTGCAGCACCAGGCATGTGGGACAACTTGTGACATTAAG

CAGTTGGAAAGGCATGGAATATCATCATTTAAGAAACATGGAATCATGGGAAAGAACACAGCACTTAGTAGACATATGGGCCTTGATTAATGCTAAAATC

ATCATTACATTCTCTTAACCCTTGCTTTGGCTATGCAAAACATGAGGCCCCAGATGAGTAGTTTTGACAAATCTTCAAGTTGCAGCAAAATATTGGCTGC

TGGCCTCAGGAGTGTGTATAAAATGGTACAAGTGATTGACAGACTAAACTTTAGCCAACGTTTTCAAGCCCTTAGTTTCTTGTAGTAAAGTTGAATTTAA

GAGATCATTATGCATAACAAACCCTGATTTTGTTGGTGTAATCCAAAACAAAAGATCTGATTATGTAATTCTGCTTGTGGTCATGATGAAAAGTCATTTT

GATAATAGACTTTCTCAACACTCCTTGCTTTCATGCTGAACTTTTTTCTAGTGAGCCCCATTTATTCTACCTTTTATAGTCCAGACATTGTAGCTCTTTA

>PtOFP9

AAATGTTCGAGTACTTTCTTCTCACAACTTACTACAGAAACATAGAAGTTATACAAACCCTTCTCTTCGTTAAGAAATTGTCTATTCCGATTTCAAGTTC

TTGCTGGGAATTTATGCATTTATTGGATTTATAAAACTCAAGAATCATAAACAGACTAACTCTTGTTCACTGAAGGAAAGGTACCATTGTACTAACAATC

TACAACATGACAATCTTCTATCCAAAAAGGCAATTCAATACGGTGATAAAGCAAACAGATCTTCCTAAATTATCACCAGAGATACATGAATACTTTAAAC

AAGCATATTTGGCTTCATTGCATCCAGGTGCAGATAGAAGACAAGCAAAGAAAATGGCCTGAAAATCTTACCTTCTGCCTTCGGAGAAGCCTTGTATATG

TCAAAGCAATCTGCAGCTCTTGGGCTGCCCATAGAAGGAACTTCTTCTCCAACCTCATTGCATTGATTCCATGACTCTATTGCCAACCTTAAACCATCTC

TTCTCATTCCAGGATCGCCTAATGCTGATACATACTCATTTCCAGCTGTAGCATATAAGGCTCCTGAAAGAATAACCACGGCCAGAACAGGAAGAAAATA

AACTGGGTTCTTCTTTCCATCCATTGGTTAGAATAGAGGAAGTTTGATATCACACATGTACATAACTATATATAATATGATAATGAATTGAAGAGACTCA

GTAGAGATGTAGAATTCGAACTTGATTGATTGAGAACCAAGTACGAAAAAATTGCTCTAGACTTTGGTCTTACTTTCCAGTTGCGAGGAACATACTCTTC

CTCATAATGAAAGCTCTACAGATATTATTGTTAATTTCCTATGTGTAACTTCGGTCGGTACAAATTCCCGTTCATAGGGGGCTACTTATGCCTTTCTTAA

ACTAACTCTCGCACCAGAAAAGAAAGGGACAAACCAGAATTTTTAATGAAAAACAATATAAGCTAGGGATACAAAAGGACTTTTGGTTACATCTGTTTAA

AATCATATTCACAAAGTTAGAGAAATCACTGGAAAATAGTAAAAGTTCATCTTCAGCCTATGCTGTAAGGAAAGGTGCCGTTTCTAGCTAACTTCTGTAG

ACGTTAAAACCTACCCTAGCAAGTCTTCATGTATTCCAAAAGGACATTACAATAAAAAAGTTTATACTACTTCGAGTAAATACTTGAGGGTATACGTATA

GAGTTATTGAGCCGTTATTCTGCAGAACAAGCAGAGGAAAACCAAAATAGGGGATTCTGGCCCTTCTTGCTGATAATGATCACACATCGCATTACAGTAA

GAATCTGACAATGACCCTTCTTGCCTTTGATGGACCACGGGTTGGCTGCACATGCTAAACTTATTACAGGGCCAAAGTGGGACTGAAAGTGGAACAATTG

TCCTAGCCTGTGCAAGTTACATGAATGCTATTATGTCAAGTGTAATTGTAATTTGTAGCTCTGCAATGCAAATTTACTCCAAATCCTTCACTGTCTGTGA

TGCAAACCACACATTGACTGTAACAGTGGGTAAGGGCATTAGCATAACTAATTTGGTCTTAACAAGCAAACATTGATGTCTGCAGCCTTTCTAACTAGAA

TACCAGTTGTTGGTTTTCTTTAAAACAAAGTGATGCCTCTATCATATCTGGAGTAAGAGAAAAGTTTCTATATTCTGTTCTTGAAATGCAATCAATCCTT

TAGACGCAAAACTTGTTGACAATACAGGACCTCAACCTACTGCAGGTGTTTATCCAATGCAGCCAAGGATTTGGACCGATTTACTTTTGTTTGTGGGCCT

GAGGCTTACCAAACCCAACCATTCTTTCAGCCTTCATTCTTATCAAGTGCTAAGATCTTCTTTTTTTCCGAGGCTATTAGCCACTGTAGTGGTCTTTGAC

ATAAAAGCAAAAAGTCACATCTCCAGAAACCAAAAATAAGCAAAAGGTAAAAGAAGGAAACAGATAGATTTTAAAGAAAACGAAAGGAAATGATTAAAGA

>PtOFP10

AATTGCGCAATGTGCTTGGTGTTGTTTTTCTATTTTCACTCAAGTGGAAGCAAACACTATTAAGTAATTGTTTGATTGCTATGTTGAAACGATTGGATCC

CCTGTTTTGAAAACACAGATTCTAAAATTGTCTCTGTCTCCGTCCCTACCAAAAAAGAATTCATGCCATTTCTATATTTGTCTCTTGCATCCAAAAACAC

TAACCAAACCTCAAGAACCTTCGATATTAGCTAAGAAATAATGCTGTCAAATTTGTTGACATAAAACTATTCCATTGCTGAACGGACAGTTAGTAGCATG

CTTTAGGCTATGGAAGTTCCGCAAAGTGAAACAAACAATTCAAGGAAAGGCCAGTTTGATCTCCTCTCCGCCGCATTGCATTAATTCACTGAAGCTACCT

TGCTGGTGTTATTCAGTGTAGATGCAAATGCTCTAGCTATGAAAGTTTCTGCCCAAATCCTGCTCAGGTACTTGGAATACAGACTACAGAGTGATCAGAG

GTCGAAGATAGGTTGCTTAGTGGCTAACCGATTCGCCTTCCTCACCTAACGAAAAGTAGCCATTCTTGTGAACATTGCAATGAGTTATGAGTCGCTGGTT

CTTCCCTTTATCTGTCCTACATGTTCTGAAAGGTCAGGGAACAGTTGTCATTTGATTAGTCTATACTGCTTCTTTAATGGCAATGGCCTTTACCTTCGAT

GCTAATCATTGCGCCAACATGTGGTGTGTAGCGTTTAGATGACAATAAGTTGCTTTAAAGAGATAAGCATATCCATTAATTAATTCAGAACAAGATTGGT

TATTCATGGTGTACCACAATGATTATCACATGCTAAACATTTCCATTTGATCTTTGTTAACAAGGAGGAATCCATTAAAGAGATCGATTCCCCTGTCTAG

CTGTGAGAATCCATGTAAGATTTCTTCCATGATTGCATCAACCCTTCATCGATTCAGTTCATCTCATCATCTAGAAGTGGTTACAGAGGAGAATCCTGGC

TACATTATGAAGCAAAACTCGAGAATTAAAACATGCTTTGTCTCTCATCCACCCACAACCTTCCTCGATAATCATTATGTTAATTATCGCCATATGGACC

ACCGTCATCAGAATGTGGTGATGTAGTTCACTGTTTTTGCCTCAAAAATCGTCAGAACAGATCTAGAACTGGATCGCAGCTCTTTGATTACCATAACCTG

ACAACTAGATGCTACATGAATAAATCTTGCATGGCATTTATGTCGTCAGCCATCCCAAATCGTCACTTCCCTATTTCATTATCAGTATCCTAGGGCTAAG

CTTGCTGCGAACACGAATGTACACAATTAGGTAAAGGACTGGAGCTAGAACTTCCTAACGAGCTGTTCTTTCGAACTTTTGGAAGAGGAAAGCTAGGCCA

ACGGTTAATAATTGAGAGCCTTTATTGAGACTCGAGAGAGAGGGTGCTTTCTACCTTAACTAGCAAGGATGGCTTCTTTAAAGATGGGAGCATGTGGGTC

AAAAGGAATGTCTTTGCCCCATAATTTAATGGATAATCACTTTAACATCATCTTCAAATTCATGATTAGCCCTCCCCACCATGCCAACAACAGCCTTCGC

TTCAATCCTTGAAAATGCAGCAAGTACACCCGAACTGATTCGTACCAGGCACGTGACACCAACCAAGAATTCTGTCAAGGCAGTGCTTCCTTTCTTAATA

CAGTATCTTAATTTAATCCCATAGGCTTATTGGAACCTCATACTTTCAAAAGCATGCCACCTAACACTGCCATATCAAGGGAATTGTTAAAGGTCTAGTT

CACATCATAATCACTAGCTCAATTAAAAGACCAAATGTGCTTGCTTTTCTTTCTCAGACAACCAACCTACTAATCTCATAAGTTATGTGTACCTCTTTCT

ACATTCAAGACATAATCATGCCTGATCTGACCCACTTGCCAATTGTGCATAAATCATTTTGTTTTTGTGCAACTCTTCCTTCATAGCTCCCTGGTTCAGG

>PtOFP11

AAAAAAAGATTTTTGATCTTTTATTTTCTTCCTGTTTTTATGCTTTTCAAATATGAAAATCACTTGAAAATGAAAAATCAAGGAGATAATTAGAGATATG

ATAAAGTTATGGATTTAAATGGTAAAATAACTAACTTTAGAGATTTCGTTTCTTTCATTTTTCTGATATTAAATTTATTTATAAATCTTGAATTTCTCTT

TTTGCATACGATTTGGACTCTGCATATGTAATTTTGTTTTCAATCTGGGTTTGATAGAGACACTTAATATTTAAAAGAGGAAATAATTATATAAAAAAGC

AATTATTAATTGACATGATAAGGAAAAAAAAGAAGAAAAAACAGAAGATCGAGAGACCAGTTACATGAAAAAAGATGTTTAATTATTAACTACATTCATG

CTTCTTCGACATTGTTAGTTGATTTTACACTTATATATCGAATAGGTAGAGCAAAAAGCTAAAGCATGGAAAAATCTTAAATATTTTGAAAATCTTAGGT

TATGAAAAATTTGAGTTTAAGGATAATTTATAATTATCAGCTCACAATGAATTATCTTAAATTCTTTTTGGTTGCAGATTTAGATAGATTTATGATATTC

TATCCAAAAACTTAAATTAATTATCTTAATTTTCTTCTTAATTACTTAAGCCCCATTTCCTACTTTCTGTTTAACCTCACAAATCTTCAGATTACTCACA

GGAACCAAAACAAACCATGGCAAGCAAAAACACGAAATGTTCTCCAACGCAATCCGAGAAACTTTCCTTCATAACCAAGACAAAGAGCAATAGCTTTCCA

ACAGCCCTTTGTGAAGAAACATCAAATCCAAATGCCAGTTTGAAGCATACTTTAAGCTCCCCTTCACTATCCTATACATATACTTCTGTGAAGGATTTGA

TTCCTTGTGTCGAAAGCAACAGTCCTTTATCACAAGGTGGTTTTTGTACTATTCGATCAGCTGAGGATATAGCAATAAGCAATGTTCTGGTCCAGAAGGC

TGCATGGTTGTACTTACAACCTTTGCCTAAAACAAGAACGTCCTCCTCCTCCTTCGAGAACTTACTTTTGGGGTCACATTTGGAACAAGCTAATGAATAC

TTGTCCCAAATTCATCAACAGCAACATAGTTGGGAAGATTACACATGCCCTTGACATGGTACGAAGAGCAATTTATGTTGTCTAGCTAGTGACTGAGTTC

ATGAGCTGATTTTCTTGTCGTTGCTTTAGTGATAGTTGCTGGACAGTTTTTCAGCTGCTATGGTTGTGCATGGCAGTCGTTTTCTTGCAGGGAGGGTAGT

TCAAGTTGCTGTTTTCTTTTGTGTTCTTTTTGAAGTCTTATATAGTTGTCTTTATGTGGTTTGTGCTTGGCATCATCTTGTTGGCTTCACTGATCTGCTA

TGGTGGATCTTGTACATATCTTTAATTCTAGTTTTCACACAAGTATTGTGTTTCTCAACTATCATATAGAGTTAATTAAAATTAATGAAATGATCTATTT

ATTCATTTTTTTTATATAAAAAATTGTTTTGAGGTACTCATGATGATATGAAAAAATTCATGATAATTTTGGATCATTAATTAGTCTTTATAAAATTCAC

ATATATCATATTCTAGAATTGTTTAAGTTAGATTTTCTGTTTCTCAATTAAGTTATTGCAAAGAATATTATTATTATTATTATTATTATATATATATATA

TATATATATATATATATAAAAGGAAAGAAAATATCATATTTGATTCAAATTAATTACTTGTGAATTTTCTTTGAAGGCATTTTAGAAAATGCCAAAATCG

ATCCCTTTGTTCTTTTAGTCAAGAAGAATGATCTAAGAAAACCATCCTCTCTCAACAACTCTAAAGAGAACAACTCCTATCCAAAAGCAAAAACAACTCC

TCTCCCTCCCTCTCTCTTCTCCCTTCTCTCTTCTCTCTCTAGTCCCCAAATGCTTTAGAGTTTTAATCTTGAAAAGGAGTCTCACCATTAACACCTCCTA

>PtOFP12

AATAATCTGGACAGACAATTTGAAAGAAAAAGGTTTGTAAGATATTCAAGATTCAGCACAAGCTTTATGCTATTTGAATTTCAAATACCAAATGAATCCT

GCAAAAGTGCTCCCATAGTTTCTTGGTCCCCCTGCCTTTACATATAAATGGTAATGGGTGGGTTGGCATTCCCCGAATAAACCTAACATGGAAAAAGTCT

GCAAAATCAGGCCGTTCCTACCCGTCTATCTGGTTTTCCTAATCCTAGCTAGAGTAGGATATATCCAATTCAAACCATTTGTTGTGACTATGCGTTTCAA

CACAAAAAAAGGAAATTAATATCCATTCCAAAAAAGCTATATATGAAAGGGAATATAAATAATTGCTCAACTAGCAAAACGGGTCCGGAAAAAAACTATA

AATAATTTTGTAAAAAATATTTAATAAATATTATATTATTATATTTTATACATGTCTGTCTCGATAGTGGACTATTAAGCAATCTTTATTCTGTGCTTGA

AACCTTTTATTAAGTGGAGATAATTTTTATTTCATAAAATATATCTTATTTATTCTTAATAAGGTGTTTTTTCTTGGTTAAGTGAAGGTTTTTGATCATT

GTTCAAGTGGATTTAGCTTAAATCAGTTCTGTTAATTAGTAAATGTTTCATTAAGGTGGTAAATGTATTTGTTTTTGACATTATCATATATAAAGTTAAA

AGAAAAAATTTATGATTAAAAAAAGCTTTTTCAAAATATTAATTAAGAGTTTGAAATTAACATGAACATTTGTATAGTATCGATATGCACCATTCTTATC

TTTTCACCTTTTTACTTATTAAATTTTGATTTTCATATGTTTTTCATTTAAGTGATGTACAAATAATAATTTAATTTTTAATATTAATAAAATTATTGTC

TTTCTTAAAAGATACAGTATTCTCGAATGAAAATTCTTACGTGCATCAAATAAGGTAGTTTTCATTTTATTATTAAGAATTCAAAAATTAAATAAAAATA

AAATGTTAACACAAATAAAAACAACACAAGAAAGACAATAATTGTAAGTGATACAAAATTCTATTTAACAAAAAATCTCTAATTTAAAAACTTATTACTT

AATATTTTCTCTTCAACTAGCCTGCTTTTTAATTAAGAGTTTTTATAACTTCTTAAATAAATTAAAAAAATGATCTAAATAAATTAAAAAACTCAAAATT

AAAAGAAAAAACAATAAAAGTCCCAAACAAGCTAAAAAATAAAAAAATAATAAAAATAATAATAAATCTAGGTGGAACAACAATACCTTCAGCATAACTT

TGTCTCACCATTCTATAGGTATGAGCAATTAATTATAGGTCTTACCATTCTATAAATGTTCTTGGAAAGCTATTCTCGTAGCTCAATTATGACAAATTCA

TAATTAACTAATCAGAACCACTTGCTTTAGAATTTTACCTTATAAACTTATCTCTTTGAATAAAATCAGTGAATAAAATCAGATCATATGTTAATGAACT

TATTCTTCTCGATGACTTGAAAATTATCCAAATAAAAGAAAGAATTTAATCTAGACTATATAACACCTAATTAGATTGGTTCGGTAATATCCCTCTAAAA

AACACAAAATTCTGCGGGAGGAGGGCTAAAATTCTGCTGGAGGAGAGTGGTTTTTAAACATGGCAAAGGAAGTTCAATTCAAAATAAAGTACTTATGAAT

AATTTATTAATCATATATCAATAATTAAATAATAATTGACTCTTGAGGTGGCCTCCTCAATCCAACTTTTTCCAAGTCCAAACAAAGAAGACGACGAAAA

ACAGCAAAAAAGTTTTATGATTCATGGGTACGCTTTAACCTTCATTATTATTATTATTTAAAAAAAAAAAAAAACACTCCTACTTTCGGTTTCTCATGTA

TTTATATATCAGCCATCCTTATACCGCCTCTTCTCTTCCTTCAAACCTCCCTCTCCCTCTCTGCTCTCTCCCCTTCTTCTGAAATATCTCTCTTTCTCCC

>PtOFP13

TTTCCCCTCTTTTCTCTTCTTCTTCTTATTTCTCCCTTCTCTCACGGTTTGCTCTCTCAAATATTCAGATCTCTTCTTCTTCTTTTTTTTTTCTTCTTTC

TATTTATATATATCATCTCTTTATACAATTACTACAATACCCTTCATTTATTTTATTCCTACATCTTAAGCCTTCAAGGGCTTTCTAGCCTTTTCCTATC

CCTTTAATTCAAAACATTACAAAATCAAAAAATGATAAAACATCTATGCATTAAACCTCTAATGCAATGTAGATTCTTTGCTATAAAATCTAAAAATCTT

GTCCAGCTGGTTTGATCTAATCCAAACACTTTGATTGTTTCTCCAACTTCAAAGAGATAATAGGATTTATAATGATGATGATTTTCCTGAGATGATAGAC

TCTAAAGCTTTTAAAATAAAGATTTGTCACTTAGACCCAATAAAGCTGGAGCACATCCAGATTGGATTCATGAAAATAAGAGTTTCTAATCCAAAAATGG

GGTTCCCAATATCCATAATGTCAACATTTAGATCAAGAGATCGAACTCAATCTGGCTCATAACCCGCAGGCTTCAATCCTTCATTCACGATTGGATCTAC

ACCAAAAATAAGGTTTTTTTTAGTGTAAACAGCTTTCCATCATTGTAAAAAAAAAAAAAAAAAAAACAAGAAGAAGAAGAAGAAGGTTGACAGCTGGTTT

GATCTATCTATGGAACTTGGGTCGCCTGTTGAGCCTATTTTGCAGTTCATATATTCTTAAAAAAGAGGTGAATATATATAATTGTTATTTGGGTTCAATT

GGCTTACTTTTTGTGAACAATCTTCACACTCCGGCTAATACAGTTACCCTGCTGCTACTGAGCTGGATTTTTTCCTGGTTTCTTGCTTAGTGATGGGGTC

AAAGATCCAAAACGCGTGTGCATGAACTGAGGCAGAAAACTTCCTTCGTTTTATTTTATTTTTGTACTGTAGCTTGTGCATTCTTGTCCAGGTGTTCATC

AGATGATGAACTACGTACGTGACTGCAAATTGCAAGGTCCTCGAGTAGATTGTAGGGGTTTTTTTTTTTTTTTTTGCCATCTCAGCAAGGGCTCCAGTAC

TGAAAATGACACCCAAACTTGGAAAGCAGAACGTAAGAGTAATTGAAAAATGTTTGGGGGACCTGGCTGCTATTTGTTTTTTCGAGTTTGCGTCTGAGCG

GAATGAGTCAAGATAACTACGGCTGGATGCAACCCTTGTCCTTCGCAATATTATCTCAACAATAGAAAACAAGAGAAGGATTAGCAGACTCGAGAGAGCT

ACGGCCAGGCCAAGTCGGAAGCCATGATCCAAATACATATTGCTACCAAAAAGTTAAGATGTCAAGCATCTAGAAACTATTTTCTGAACAATTTTTTGGC

CCTGAGCAACAAGCATGGTCTCGATTGCTTCTTTCACGTATCTCTCAATGACATTTCCTAAAACTTTCTATCTCGTCTTCGAGATCCATGGAAGATAATA

GGAGGCATCTCTGAAAATGATGGCGGTTTATTCCAGCCATGTCTCTATTTTTTCAACAAAACATGTCTCTGTTTTTTCTACTGTAAAACAATGATCAGAC

ACCACCGGCTAGTAAACTCGCTTACAGGTTGTATTGAAAATTATTAATATATGGAATTGATCTATATTAGTAATGGTTCTATCTATTAAAAAAAAACTAT

TAGGTAATATATTGGGAGTGTGCTCTCTCATTTTGCATAACCATAGAAATCATCATGAAAATTATTCACAGCCTTCATGTAGCTTTCAGGCAAAAAGCAT

ACATCATCACTTGGATTTGGTCATTCTACCCAGGCGTGCAGATCCTTTAAATTAATACTTTTGAAACCCAGTGGGTGCAAGCTGTTGTCTTTCATCTTGA

ATGCCGGCCTCTTTCCATTATATATCCAACCTCTTATAGCTAAGCCTTAGCACGTTGTCTCAAAACTACAGACGTTCTCATGGTCATGAACTTCCACAAG

>PtOFP14

TATGCCAAGTGGTAAAGGAAAAAAAATAAATATCAAGGTTTTTTCTAAACATTATTGTATTAGGGTTTTTAGCTTTCTTTTTCTGTTGGTTAAGTTGTTA

ACATAAGTCCTCGAACTTCTAATTTGTCACTAAATTTGATCATTTTATGAGAATTTTACAATTTCTCATTCTACACTTGGGTAATCTTGTATTTGAATTG

TATAAATATTTAAAGGATTAGGACGTCATTATAAGAAATTAAAAGGTTGGAGGGCTTATATTAAAACATAACTTTCGAGAAGGGCCGAAAGTTAACTCTC

AAACAATTATCACAGTTCTTGTGCTCGAGAAATTATTGGGTAAACTTATTGACTACGTACTTCCTTATATACCTTTGTCTTCCGTCTTGTCATGATAGAT

GTCCCTGATAATCTCTCTTGTCCACTCTTAACTTCTTAATAGAGGCCCAATTAAGCCAAGCCCATTTAGGCATTTGGGCTTTATCAATGTTAGAAAAACT

AAAAGCCTGACATGTCGAGGGGCAAGCAAAACCATCTACTCTGCTCCAGGTTTTTTAAACTGTTTTTATTTTTTGAAACTTTATTAAAATAAATATTAAA

AAAAAAACTAATTTATAAAAAAACCATTTTTTTGCAAAAACACTTCTATTACCAAAACAAACGAGGCACAACCATGCTGGAGACAATATTGAGTGCAGAG

AGAAGCTTTAAGCTTCGCCAATGACCATGTGTCCCCCAAGAGGGCAGGGAATAAAGGGAAAGAGACACTGGCATGCAAACATCATTTTGTTTCCTTGGAT

CCCCACCATGTTTTTGAGAAGAGGAGCTTCATTGTATTGACTTTTTTTTTTCCTTTTTCCATTGATAAATTTGTTTAATCCTAACATAAAAAAAGCCAGG

CTCCTTTTTTCTTCCCTGTGCTCTAGAAGTGATACAGCATAATGCTAATTAAAAAACATGGAGATGACAGGAGCTGCTTTTGCTTCCTTTTCCACTGCTA

TATGAATTAACTCGATGACATCACCGTTCATGAGGTAAAGCCTGAATCTGATCGTGTAATAGCCTTTCAGAGTTGAAAGCATATTTCACTGCGACTGCTA

CACCATTGAGGTCTGTGCATGCCTTGAGCAATCTGCTTTACTTGCCTATCACAAACAAGACCCAAGATCTTCCCTGGATTTGAATTTACAGGCTCGATCT

CTCGCTGTTGAATTTGCATGATCATTCATCATTGATGCGGGGCAAGATCATAGGATGAGAGACTGATTAAAGTGCTTAATCACATAAAGAGCGGTCAATG

ATTAGCCAATGGCTCCTCGACGGAGAAGTTTTCTAAGGTCATGATTTCAAAATGCATTGATATCCATGTCAATGTATGCAATCCTACTTTGAGATACGAA

TTTTGTCTTTGCTTTTTTGACTTCCTAGCCACCGAAAGGAATTTAAATAATTTAAAAAGATTAAATAATCTATTTACTACCAGAAAGGCTAGTACTTTTA

TAGATTAAATTGAATTTAGATAAAAAGAAGGAGGCTCCACGTGTAGAATTAATTCCATACCATCAGAGTTCCCATACGCTAAAGAATCCGTCAGTGTACG

GTTGGATACACCGGATCAACATGATTATAGTCTCCATTTTTGAGAAATTCTCTCCTGGGATAGACGTAGCTAGCTAGCTTCCCTCCAAGAATTATTCAAG

AATACATGGAAAACGATTTGAATTTTTGAGAAATTCTCTCCTGGGATCAACATGATTATAGTCTCCGTTCAGAACGTAATACCAATCTGGAGATAGGTTT

AACAAGTATAAATTACTCCGTTTTTCATTTCCAAATCAACCCCAGTATTTAACCCTTTTTATTTAACAAAAACAAGTCAGTCTCCATTTCCCCAGCACAG

GAAAACCAACAAAAGTACCTGCTTGGTAGAAAAGGTACAATATATACGTCTCTTACATTCAGTGATTGCTTGGGACATGCATGTGTATTTTCTCTTTTTC

>PtOFP15

AAATCTACTTTAATTGCCAATTAATTCAAAGCAAAAATCAAAATCATTAATTGTGTGCTTGCGTTTTTTTTCTTTCTTTCCTTCCCTTAGTGTTTTTCCT

TGTCTAGCAGTGCTGAAAAAGTAATTCTCAAATTAGCATAGTACCTTGTGTTGCGCATATAGGACTTGCCCAACTAATAACTTTCTAGTTTTTGTTCTCT

CAAATAATTAACTCATGCAAGAGTTGATATGTTTTTTTTTATAAAGATACAATAGCATGAAATAAATCAAAATAATCTAATGACTAGAAAAAAAGAAGAT

AACATGGCAAGAAAAACAAGATAAGAGATAGAAAATAAAAAAAGAATAAGTCATTGAAGATACATCAGAGTTCCAGCGATGAAACTTCAACTTACTCTGA

AAAGATATCGATAAGACAAATTTATAAATATCATGAAAAACCAACTTTGAAACTAGTGATGAGATGATAATAAGTGAGCATGTTGTTGCCTTTAGACTCT

GCACGTGTGACTCACTACTTGCTTTGAAATTTGCTTTACATAGTGTCTATGGATTCATAATATCTTTTTAGTGGAATTTGGAGATTCATCATCGAAGCTT

CGGTATGTATCCGATACCTTATTTTTTTTTTCTTTCTTTTCTGGATTCATAATATCTTTTTAGTGGAATTTGAAGATTCATCATCGAAGCTTCGATATGT

ATCCGATACCTCATTTTTTTCTTTCTTTCTTTTCTATTTTCTTTTCTTTCTCTTTTTTTTTTTGCCATGTCCTCCTTTCTTTCTAGCCATTATATTATTT

TGACTTATTTCATGTGATTGGATCTTTATATATATATAAAAAAGCATGTTGACTCTTGCATAAGTGAATTACTTAAGAGAAAAAAAATTTGAAATGATAT

TTGTTAGTTGTGGCATGTGCTACAAAGTAAGTTTGTTTCCTTCCTTCAATTTCTTAAATCTGAACATTTTTAGAAATTTATTTGCTTCAAAGTACTGAAA

TCATATTTTAAATTTTTTCTAGAAAAGATTTTAATTGTAAGCTGATTCAAAAATGTCTTTGATCATTGAAAAAAAGTTGATCATTAGTTTAAACTCTTTT

TAAGACTTTTTATTTATAAATAAATTGAAGATTTTGGATTTGCAAAATAATTTAGACGTTATAACATTATAGAAACGTTAGATCAACAAACAATTAGAGT

TCCTTAATTTCCTCATACATCTGGATTATCTCCCGAAGAACAACAAAAGAAAAATAAGTCATTTTTCAATAATAAAGGATAGCTCAGTTGATCAGGTTTT

AGATTTACATTTCAATAATCACCAGCTCGAGTTCTTTTATGGTCATTGAAGATTTACTTGATCGTTAATTTCAGGGTCCTGTAAAATCAGTGGAGATACA

CATAAACTGACTCAAACACTAATAGTTACAAAAGAAGAGAAGTCATTCTTCCTTTTCTTAGCACACATGAAAACCAATGAAGATACCCTATTGATTGACA

TACATTTTTTTTTTTAATGTATTTATTTTTATATTTCAAAAATATTTTTTAAAAAATATAATATTTTTTATTTTAAATTAATATTTTTTTATATTTTTAT

ATTGTTTTGATATATTAATATTAAAAAATTAAAAAATAAAATATATTATATTTAATATATTTTTAAATAAAAAACACTTAAAAAAAACAAACACTACTAT

TAATTATTGCTCAAGTGAGCCTCCTTATCTCTTAATACATTTACTTCTTCGTTTGTCCATAATAATAATAATAATAATAATAATTACTTTCTTAAATTAT

ATTGGTCAATATTTAATTTTTAAGAGCAGATGGTGGTAAGTGGTAACAGTACATTTTCACCCTCTGGTTTCCTGCTCATTGAAATAGAAAGACAAAATTC

ATCTCACCCCCTGTATAAACCCTTCATCTTCCTCGAACAAACAGTTCAATGCTTTGAAACTATAGCAAATTAAGCACTTGAACCAAAACAAAAGACTACA

>PtOFP16

AAATATAAAACTAATTTATGAATACGTAGCCACACATCTTGTCTTATAGTTTACTTCCATATAAATTGTTTCTTAACTTTTTGATGTGAGATAACTATAT

ATAACCTATTTTTGCATGGATTGTTTCTTAACTTTTTGATGTAGGATAACTATAATACACTTCATGCATCACATTTATTTTTGTAACCTACATCCATATG

GACTGTTTCTTAATTTTTTAATGTGAGATAACTATATATAGCTTACATTCATATTGATTGTTTCTTAACTTTTCATTGCGGGATAACTTTATTACACTTC

CTGCCTCATATTTTTTTTTATAGCATACATCTATATGAATTGTTTCTTAATTTTTTGATGTGAGATAATTATATATAGCCTACATATACATGTGTTGTTT

CTTAACTTTTCGATTTGAGATAATTATACTATATTTCAAGCCTTATATTTTTTATAGCTAACATACATATAAATTATTTCTTAACTTTTTCAAGTAGGAT

ATTAAAAACTTCAAACATATATTTTTTTATTTTCTCGGGTTAACCTATGTGACCCGGGACCTGACCTCTTAGTTTGGTCAACCCTTAAGTTAAATTAATC

TGTCAAACTTGCTACCTGAATCATGAGATTGAGATAACCCCATAGAAAAAAAATCATAATAAATTACGAAGCTTAATTCCCAATAAATTCATTGTTGAAG

GATAAAATTGAAAAATTATTAATAGAAAAATATGACATAATAAAACAAACCGAGTCTACCAGGTTAACCCGCAAAACCCATAACCCGAGTCATGATACTA

GGATGACTTCGTAGACATCAAACTGAAACAGACCATAAAGTCTAATTCCTAATCAACACAATGTTGAAGGAGAAAATTAAAAAACAAAACTCGATTCCAC

CGAGTTAATCTACTAAACCTACAACACGAGTCATGAGACTATAATAACCACATAGAAAGCAAACTGCAACAAATCATGAAGCCCAATCTTGAATAAAACA

AATGTTGAAGGATGAAACTAAAAAAATAAATATATTTAAAGAACAAACAAAAAAAAAAGAAAAAAAATTATTGCAATGAATAGTAGGACACAAAAAAATT

ACCACAGTTAACCAGGGTTATTCTACAAAACCCATGACTCATGTCGATATAACCTCATAAAAAGTAAATAAAAAAACAACTTAAGTTAACTCGGGATAAC

ACGCCAAACTCATAACTTGGGTCATGAGGCTGGATAACCCAATAAAAAGAAAATTGAGATAAAATTATGAAGTCCAATTCTCAATAAATTCATTATTGAA

GGATGAAATTGAAAAAAATTTAATCTTAAAACGAGACAAAATAAAGTGACCAGAGTCTAATTGGGTTAACTTGTGACTCGGGTCATGAGACCGAGGTAAT

CTTGTAGAAAGAAAACTGAAACAAATCATGAAATCTAATTTCTAATCAACCCAATATTAAAAGATGAAATTTTAAAAAAATATATCAATTAAAAAAAAAA

CTAAATTCAACCAGGGTAACCAGCTAAACCCAATACCGACATAAGATTAGAATGACTAAATAAAAAACAAACCGTAACAAATTATAAAGTTCGATCCCTA

ATAAATTAAATGGTGAAGGATAAAATTAGAAGAGAAAAAATAAATTAAAATATATTGTTTTAATAAATAGTATTTTGTGAGATTATGTACGATAAAAACA

CCATCTTTTTAAAATTTTGTTTGTTAATATTAATGTTTATATTAAAATAATAATGTTAATATGAACAGGAAAGCTTAGCTACTCATACGTTGTGGGCGAA

CAAAATATGAGCGCCATTAAATTCGAGAAATAAAACACTGCTTTAAGGACTCCAAAACTCATGCTATATAAAAGGTCCACATTTACAGTCTTTTACTTCT

GTCTGGTCGCCGCTGAACTAAAAATACAAAAGACTAATTACACCATGCGCCATCAAGGCATTAACCATGATGGACCCTCCTCTCCATCACAATTAAAAGA

>PtOFP17

CAGCTCGACTTATGAATTGATTTACTAAACTACAGACCCAGCATATAGACAGAGGCAAGTTAGTACAAAAACTCAGATTTGTTTTTAAAAAATTATATTA

TTTTTTGTGTTTATTTTTTTAAAAAATATGAATTTATTTAGGTTAATTTACCTAATCCATGAAACATGCTAGTGAGTATTTGTTTTGGGCCCAACAAAAT

TGATTCGTTAAGCTCGAGAACAATTCGGTCATTGTGGCTCCTAGCTTTTTAAAACGTATATGGCCAGGGGAAGGGCCACGTAGCAGCTGCACAAGCACAC

CAATATTTCCAAAAGGAGAGATTTTAAAAAATTACATGTTTTACCTTAAATGAAATTAATTAGATCCTTCTCCATTTTAAATTCATTTCTTTGCTTTCTT

TAAAAAAAACCTTCATTCTTTTATTAATACTGAAATTAAGAAGAATTAAAGGATAAAAAGAGTTCTGATATTTATATTAATTTACTATTTTGAAATAGAA

GAAATATAGATAATAAAATTTTTATCTTTTTTATTTTGAAAATACAAAAAAATATTCTAGAATTGTTAAGAAGACATTTTATTTTATGTTTTTGTTTTCT

AACAAAAAAATATTTATATCTTTTTTATATCTATCAAGTATAAAAAGTAAAAATAAAGGAGATAAATAGGAAGATATGATATTTTTGTAACCTAGTAATT

AATTAATAAAAAACAAATCTTGTAACTCATAAAGGAAACTATAGACAATTCAGGGATTTAAATTGTAAAATAAATAACCAACGTGAGAGAGAGGGAGAGA

TATATATCGAGGTCAGTTTCACAATGATCGATAAGATAGTTTAATTATATATTAAGGATAATTTATATATTCCGAGGTTAAATAAAGAAAAAGGATGAGC

TCATACTAAATTATCTTAATTTTTTATGTTACGTGGATATTTATGCATGGTTGAATATGCATGGAATATTGTGATTTAAATTGAGAATATTCAAATTCTA

AACTTGAAATTAATAACAAAAAAAAAAATTAAATTAAAAGAATATACGATATTCTATATATCCAAGGTGATCTTTCCTAAGCCTCATTAATTTCCTACTT

TCTTTATTTTAACCTCAAAATTCTCCACATTTCTCAGAAGAACCAAAACAATGGCAAAGCAAAAACACCAAATGCTCTCCAACTCGATCCAAGAGATTTG

CCTTCATAGCCAAGACTAAAAGCAATTATAGCTTTCCAAGAGCGCTTCGTGAAGAACCATCGTATAGTACTGATCTAAGCTCTCCTTCGCTACCCTTTAC

GTACGTACACTGCAGTAAAGGACTTGATTTTCTGTGTCAAAAGCCATGGGATCATCAAGTCCTCTCTATCAAAAGGTGGTTTTAGTACTGTTCAATCAGC

TGAGCAGATAGCAATAAGCAATCTTCCAGTCCAAAAGGCTGCATGGGTTTATTTACAACCTACGCCTCAAACCATAATTAAGCACCACCACCACTGCTTC

AAGAAAATACCTTCGGGGTCACGTTTGGAACAAGTTAATCTCCTATGAATACTTGTCTGAAGTTCATCAACAGCCAGCAACATAGTTGGGAAGATTACAC

ATGCTCTCTTGATAGGACACGAAGAGCAATTTATGCTGTCTAGTTAGTCATATGTTTTAACTCTTTGAATTCTCATGGATAAGTCTTCGTTGACGTTAAT

TTCCAGCGAATCAGAATTCCTTGTGGCAATAATTTTAAGTTCATGTGCTGAATTTTGCGGTTTCTTTAGTAATTGTTGTTGAACAATTTCTCTGCTGCCA

TTATTGTGCATGGCAGTTTCTTAATCTCTAATCAGGGAGGATAGTTTTTTTTTTCCTTTTATGTTCTAGTTCCTTTTTTTTTGTGATTAATTTGTTCTAG

CCATCTTCTGATCATTGGCTTCACTGATCTTCTTAATCATAAAAAAAATCATATCTTCCCAAATCGTATCTGATCTTATTCTAAAAAAATAAAAATGGCC

>PtOFP18

CAGGAATTATTTCCGACAGCTGAAGCCGTCGGATCATTACAAGATCCTTAAATCCAACGTTGATGCTTTAAATCTTATCCAGGTGGGTCTCACGCTTTCC

GATGCAGAGGGGAACTTGCCCGATCTGGGAACTGGAAACCGGTTCATCTGGGAGTTCAATTTCAGGGATTTTGATGTGGAGCGTGATGCTCACGCTCCGG

ACTCGATTGAGTTGCTGAGGCGACAAGGGATTGATTTCGGGAGGAATAGAGAGGAGGGGGTTGACTCGGCGAGGTTTGCTGAGTTGATGATGTCGTCTGG

GCTTGTCTGCAACGAGTCGGTGAGTTGGGTTACTTTCCATAGCGCGTATGATTTTGGGTACTTGGTGAAGATTCTCACGGGCCGGGACTTGCCGTCTGGG

TTGGTGGAATTTTTGAGGGTGTTGAGGGTGTTTTTCGGGAATAAAATTTATGATGTGAAGCACATGATGCGGTTTTGTAAGAGTTTGTATGGCGGGTTGG

ACCGGGTGGCCAGGACACTGGATGTGAACCGGGCGGTTGGGAAATGTCACCAGGCTGGTTCGGATAGTTTGCTGACATGGCATGCTTTTCAAAAGATGAG

GGATGTGTTTTTTGTGAAAGATGGACCGGAGCAACATGCTGGTGTTTTGTATGGATTAGAGGTGCTTTGCTAGAAAATTACGTGCGTGATTTACGCATAG

ATTATCTGATTTGTTAAAATAGAAATTTGTAAATTAAGTTGATGAGACTTGGAAAATAATTAAAGAAATTCTCTTGAGGTATGCTACTATTAATTCGTTG

ATTATGGTTATTTTGATTCGTTGACTCGGTAAGTCAAGAAATAAAATGATTTTTTTTATTATTATTATTAAAAAAAAAGCATCCCCTGTTTGATCTTGCA

TTTCCCTTGACACCATTTGTCAAATGGTTGACAAGTCCACACTCCACGCTGTTTTCATTTTGAGTTTCTTCGGCCTTTTTTTCTGTTCTTTTTTATTTTT

CAAATTTCCAAATTTCTTCTACAGCTATGGTTTAGCTATTTGATGGACTTTGGGTCTCTTAGACGGGCTCCTTTATTATTGTTTAGTTAGGTCTGTTTTG

TAAGTGCAATGCTGAGCTTCAGCAGGCCCGTAATCATCTTAAGGTCTATTCATTTTTAAAGCCCATATTAATTACCTGTGCGTCTTTTGACTACAATGGC

AGAATGAAATGATCTCGTGAATGAAAATTTGAACCAGCACTGGTATAAAGAACTACCTTACAAGGTGGCTATTAAAGTGATCAGTGATGCTATGTATTCA

CTCATAAGTTCTTGAGTTTGAATCTTAAAATCAGAACTTAGAGAAATGCAGGTGGCTGATATAGGAAGATTTAATCTCCATTGATATGTAAACTAATGCC

TGACAGCGTAAACAATATTGGCTGTGAACCAAGGAGATCCTTAAAGGAATTTCAGAAGGCTTTATGATCATTTCAGATGTAGATAACTGTAATGGTTCTC

ATGTGTTCATGTTGTAAGATCCACCTAAAAGTAATTAAATGCTAGCAAAAACATTGTAAAAGCGGATTATGATGAATTATTGTCTTAAGATCATGTGGTC

GATCCACCCTCAGTTCATACATTTTTTGTTGACCTACCAAATGACAGTACATCTGGTAACTTATGATGACTGGCTATGCCAGCCAGCTCTTCACAGTTGC

CACCACATGTTGCTGCATTACTAGTTTCTGACTTGATTATACAGTACAAGAAAGTACTGGTCCATCAGACCAAGACAGTCATGCTCTGTTTTTTATTGAG

AAGTGGGATCTACTGTACAAATCTCTTTTGTGAGGTTGCAGTAATGCTCATCATGGAAAGCTCTAAATGATCTCCTGCTTTCCATCCAACCACAGCAAAA

GATGCATCTTTTACAGTTAACACACTTGATCCCTATATATGCAACTTGAGAATAGATATAGAGTGCATGAGTGAGATACAGAGACACAAAGGTCAAGAAA

>PtOFP19

TTTTTTATAATATATTTGCTTCGAATGAACAACATTTTTTTTCATGCTAAACAAATTTTTAATCTGGTCGCGGCGGCATAGCGCCGGGTAGACAGCTATA

TATTTGGTTAGTGTACTCGGTAGGAATTTGGGAATGTACGCTTTGAATCGATGAGTACCACGGTATACGATTCCTGCACTCGGATACCGATATTGTTGGT

AATTGATGAAACCAAGTGTCCGTATAGGACTAGGATGAGCCTAGGTGTTCTTTAACTTCGTGGTTCTTGTCTTCTCCATTACATAAATTAATATAAGACT

CTCCAATGTTGGTTGTGTCAGTACCAAATATAGTAAGGATCTTGTTGTGTTGGAACGTAGAAACACAGGTATAAAAAAGGTATTTGAGTTCTCTAATTGT

GCGAGTAGAAAATTAAAAATAAATTAGTGAGGGCTGTAAGCATTATTATTGCTCAGGTCTTTGGTCAATATTTGAATTTGATCATCAGAAAAGATAAATT

TCATGCAACCAAGATTGCAACTAATTAATTGCATTAAGGATTGCAACTAATTAATTGCATTAAGTATTTCTCTCCTTTCTTTTCTTTTGATTTTTTTCCT

TGTCATCAGAGCTTATATCAAGAGTCTCCTTGCAATCATCTAGGTAGTGACCCAATAATAAGAGTTTGGGACTAAGAAATTTGTTCTCTCTGTGGTCTTA

GGTTCGAGCCCTGTGGTTGCTCATATAATGACCACTGGAGGTTTACATGGTCATTAACTTTAGGGCCCGTGGGATTAGTCGAGGTGCACGCAAGCTGGCC

CGGACACCCACGTTAAACTAAAAAAAAAAAAGAGTCGCCTTGCACTTGTTTTTTTTCTTTAAATGTAATTAGGGAATTAACCATATATAAGATAATTTTT

TTTTTATTAATGTGTAGTGTTCGGACAAGCTTATATGTATTTTAACTAATCTTTATGGGCTCTAAAATTAACAATCAGATAAGTTTCTAGTAACTATTAA

TATTAGTAACTATAGAGATTAAAACTGAAATCATCCAAAATTTAATCTCAGGCACTTAATGCTGTAATAATTATTATATTTTTTTTTTAGTTTAACGTAG

GTGTTCGGGCCAGCTTTCGCGCACCTCGACTAATCCCACAGGCCCTGAAGTTAACGACCATGTAAACCTCCAGTGGCCATCATATGAGCAACCACATAGC

TTAAACCTGAAACCACATTAAAAAAAAAAAAAAACAAAGTCTCACTGGTTGAGACTGTCAATTATTCAGATGAGAGGATTGTGCAATATGTTTGTTGTCC

CAAAAAAAGATATAAACGAGGCGCTGATCATGTTGAACAGGGTACGACACTATATATACCATAAGAACTCTGAAGAAACACGTGCATGTGGTGATGTCTT

TTGGAGCATTTTAATCTCATATACATTCTACTCCACGTCATTTCATCTCATGAAAAAAATAAAAAAAACATTGTACCCCAATGCAACGGCAACTTGATGG

TTGGTAAACTGTGTTTCTGCAAAATAAAATAAAATTATATATATTATTTTTATATTTTTAGATTATTTTGATGTGATAATTTGAAAAATATTTTTTAAAA

ATAAAAATAAAATATTATTTTAATATAATTTAAATAATAAAATAATTTAAAAAATAATTATTACTACACTCTTAATACTCCTTACCCCACATCTCATAAG

ATTTTCTGCTTTAGGCATGCGCAAGACACAACTGAAATCTACATTAACCATACACAATATTTTCTTCAAGGTGTCCCCTTGTGTATAAGCCTAGATACAG

AAGCTTGGAATAAAAAAATTGTATGGATGGGACCGAGTAGAAAACATAAATAAATGTTACTTCGCTCACTCCTCCGCGCCTTGCACAGCAGCTCTCCGAC

CACATAAAATCCCTACCATTCAACCTTCCTTCCTCTTTATTCCCATTGCCACAGTAAACACATCATAATCTCTCTCAGTCTGATTCCATCAAGAGGAAAA

>PtOFP20

CACACCATCATGAAAATAATGTGAAAATATTGAAGGATATATGATATATATTGACGTTATTTGTGAATATGGCAATACTTATTTTTTATTTAAAAATATA

TTAAAATATTTTTTAATTTTTTTTAAAATTATTTTTGATATCAATACATCAAAACAACCTAAAAATATAAAATAAATTAATTTCAAACTAAAATAATTAA

TTTTTAAAAAATACTTCTTCAAACAAGTTTGCAATCATCCCAATAAACATCTAGGCTACTGCAATCATAAAACAAAATAATATTAAAAGCCCCACCCATT

AAAAAAAAATATGAAAAAATTTAAGGATATAAGTTTTTTATTTTGTTAAAAGATAATAGTTGTTTTTTAAAGTGTTTTATTTTTAAAAAATTATATTTGA

CAACAGCATATTAAAACAACACAAAAACATAAAAATAATTAATTTTAAACAAAAAAATTAAAATTTTTAAAAAACACTGCTTTTAACAAGTTTCCAAACA

TCCCAATAAAAAAATGTGAAAAAAATAAAGTATACAAGATGCAGAGGATATTTGTGATGTCATAACAATTTTTTTAAAAATATTTTTTATTTAAAAATAT

TTTAAATGATATATTTTTTATTTTTTAAAAGTTATTTTTTTATTTAAATATGTTAATCAAATGCAATCTAAACAACATTATCTAACAACAAAGTACTGTG

GGGTTTATTTATTATATTTATTTTTTTATTAATAACATAGAAGTATGGTGCCTCCAAAACGGCCCCAAATAGCAAGCCAAGTTAGTCTATTCAATTTTTA

GGTTATTGAAAGGACAGCCCAAATTTCACTTCCTGGTTTGTGAGGTCATTCTGATTTATTTATTTTATTTATTTATTTTATTTGACATCTCTATACAATT

TTATAGAAGTGTAACAATGAAGAATTACACAAAGTGGCCCATTTGTCCAAAAAAGGGTCTTTTTTCTTAATGCGTTTTCAGAGCCATTAATTAACAGACA

GATGTAGCGAGCAGGGAAAGAAAATAAAAGGAAATATAGTACTATTATTCCAATAAATGAGATACCCGAACCGATGTTCCGGGTTAATTCAAGACCGATT

GAGTTAAGGCCTATAAATTTTGTAAAAAATAAAGGGAGAAATTGACTTGTTGATCTGATAAAACATAATTAAAAAATTTAGACTCTTTTTAAAAAAATTT

CTTTAACCAATGTTTTTTAATTTCTTTTAAAAGTTCAGCTATATTTATCTAAATTAACATTTCAAACCCGTAACATGGTATTTTCTCGGAAGGAATATAA

TAACTATGATATGGATTCTATCTTTTTAATCTCACAGGTCTTGGCTCATAAATCCGTTGGCAAGGTCAAGTGGGCGTCATCCAACCGTTCTTGCTTCAAA

AGAGTCACATGAATCTTGCCTCCTATCCCATGATTTATTTTTTCAATTTCCTCATGAAGTTCTTAAATTTCCTAATGTACGTGTCTCTTTCGATTACTTT

AATAATCGAAACAGCGTTTTCTAGACCTTTTTTTGTATAATATATGATATCATCATGTGGTCCAAAACGAGAGAACAGCAGAGCTCAGTTCCACAATTAC

GAAACACAGCATGCTCAATTATGTTTCTCATTTGAGTAATTTTTATTCAATCCAGCTTGACTGTTTAAGGGAATCCAAGAACACTCACTTGAATCTAAGC

AGGCGCCATGTTCACACATCTTTGCATGCAGTAGTTTTGACAGAGAAATTGCAGTAACTTGGAAGAGATGTGTTGTGAAAGGGATAGGAAAACTTTAGGG

CACAGGAAAATCAACTGATCAAGGCCAAGGGAGCTCCATGTATTAGCAGCTTGTGAGCATAAAATATTAGCTCAACATTATAAAATTCATGCTGACAAAA

GCTGTGCTATGTTCAGTTGCTTTGAGGAGGCATTTAATATCATATTCAATTATGGAACCTAGAATTTAATACACTCATGACAGGCCCTACCAGCCCCATT

>PtOFP21

GTATGTTAGTAATGACAAAAAAGGGAGGTAATGGTGACTTATTTCTGGGCAGAGAACACTGGTTGGTGAGTGAGCAGTAGGGCAAAGTTGAAGAAGAGAC

ACAATTTGCCAGCAGAGGCTGGCAAATAAGGCATAGTTTTGGTGAAAGGCTATACGGAATCGGAAAGCAAATTATCTGTAATGGTGAATATTAATTAATT

GTTAGAAATTTGAGTTTTATATTTGTCTTTTAATTATTTTTTTTGCCCTTTTGAGATATCATTTTCTGGTGGTGAATAGGTTTTTTTTTAATTCATATTT

TTTCAGATTCATCTTTTAGAATTTATTTAATTGGAGATTTTTCTTATTTTTTAAAAAATACTTATATAGCTTGGACATTTTTTTTTTATGTTAGGATCTA

GTATAATATATACTTAGCAGTTGTGACTTCGCTTCTTATTCGAATTTAAAATGGTGATCTCAATATTATCAAAACTTGAATGTATATGTGAGGGAGAGAG

GGAGAGAGAGACCATATCAATACAAATTATTGAATGGAAAGACCAAATAAATAAATAAAAATTAAAGGGTTAATTAAGATGATTTTTTTATTTTTTACTT

TTTTGTTCGAGAGGAAGAGGAGAGGCTTAATGTTTTTTTTCCTTCCGTTATGAGCTACTGAAATATGTTTAATTAGGCCTAACCTTTGGAAAGGAAATGG

TAAATTAAGAATATCGTATTTGTTATAATCATTCATCTCTACATTATTGGTTCCATTTTTTTTTATCTTATGAATGGAAAATATTACTGTTGTTCCTTTT

TTTTTTTTAAGTACACAGAATATTAACCGTTGGCATTCAAGAGAAATTAACTACAAAACCAACATTAATTACCGTTAAATAATAAAAAAAAGCATTCATG

TGGCAGAAAAGTAATAAGCAAGATGTAAGAAATTATGATGAATGCTTTTATTCTATAATATAGATAGATAGATGCTACAAGGCATGCAATGAATCAAGGC

TGAGAGAGGGTACGTAATTAAGCAGTCAAAATTGACGGATCAGTTAAAAGAACAGATTATCTAAAATCTCTTGTGCATTTAAGATCTGGAAAATAAAGAG

AGAAAGGCATTAAATTCCTTGAATGTGATTGAAGCAACTGTAAGGTAATTTTGCATGCATCGCTGAGATTGTCAATTATTTGGATGAGAGGTACTGTCAT

ATCATATCATATCAGATCAGATCAGATCAGATCATGATGTTTGTTGGCCCAGAAAGATGTAAATGAGTAGTTGATCATGTTAAAAAAAATACAACAACAC

ACAGAGACACACACAGACCATAAGAATTCTGAGGAAACATGCGCATGTGGTGATGTCTTTTGCAGCAATTTAATCTCAGATCATATATACTCCACAACAT

TTCAGTGATGGTGTTAAATAATTACCCACTGCAACGGCAACGGCAACGGCAACGGCAACGAAACTACCCCCACATCACATAACATTTTCATACGTACGTA

CGTAGGCATGCACACAATACACAACTCAAATCTACATTACTCTACACAATATTTTAAGTCTAGAGATGTCCCTCTGTATATATATATATATATATATATA

TATACACATGTAATTATTAAATTTGACTTGACTGGAGACCTGACATAAAAATATTATACGAGTAAAGCATGGTAACTCGAGTAAATATTTTTTAAAAATA

AATTAATGTAATTTTGAGTTTTCTTTTTGGACTAATCAAAGTTATAAATCAAATGAGTGTTTAATTGAGTTACTTGATTAACTTGGATAATATTTTTTAA

AAAAAACCAAAACCCTATCTAAATTAGGTTTTAGATTGACCAACTACAACTAGACAAGTTTAATAATGGTGAGACTGCATAATCCTCGCTACAAAAGCCT

TGAATAAAGATAGGAGTGGAAGACAAAAATAAATGTTGCTTTTCTTACTCCTCCACAACCTTGCACTGTTGCTCTCTCTCTCTCTCTCACCAAATAAAAT

>PtOFP22

TGAAGATAGAATTATAAAAAAGTCACATGAATTTTGCCTCTGGTTTGCATTTTTTCTAATATTTCAGCCGTAGAAAAAACATATGTTCGTTTAAAGAAAT

GAAGATAGAAATCTAAAATGATTTTTTTTAGAAAATATAATCTTTAATTCCCTAGTGTGCCTCATTAAACCGTTCTTTTTTTTAAAAAAAAAAATCACAT

GAATTTTGCCTCATTTATTTTTTCAATCCCATTTCTCCTTTTCCAAATGAAGTTCTTGAATTTCCTAATGTATTTTGTCTCTTTCAATTACTGTAATAAT

TAAAAACAGGTTCCTCGACATAGTATATGATACCATCACGTGGTTCACGGCGAAAGAACAACCAAGCTTACCTCCTCAATTACGAAATATATCGTGCCTA

ATTAGGGCTTCTCATTCAAGTAAATTTTATTCAATCTAGTTTAGACTTGTTTGGAAACATAGTTAAAATTGTGTTTTTTTTAAATTTATTTATTTTTTGT

AAAAAATTATATTTTTATATTTTCAGATTGTTTTAATTTATTAATCTTAAAAAATAAAAATAAAATATTATTTTGATGTATTTCTAAGTGAAAAGTACTT

TTAACCACAACCGTTATCATAATCCCAAATAGACTTATCTGCTGAGAGAATCTAACTCTCTTCATAATTTGTTTAGCTAAAACAATCTTCTTTAATTTCT

TGAATAATTGGTACTTTAAGTGTCATTTTCAATTCGAGTTTAAAGAAAGAGCCTGTGAAAAACAATATTTTCTTTCACAACCCTCTAGCATGAGTATGAA

CAATCGATTTAAAGCTATGGTACGAGAAATAGTATAAAAAAGTTATTCTCGATACGCAATATTGAGAATTATTCTAACTAGTTTATTTTTTTTATAAAAA

ATCATTTGATTAATTTAGGATAAGAGTTTAAAGTGTTTTTCAAGAACACTTGGGATGCGTGTTTGTTTTTAAGATTCAGTTTGAATTTTAAAGTGTTTTT

TAAATTATATTTCACTTGAGAAAACATTTAATTGATGTGTTTTTAAGTATTTTTTTTATGATTTTAATGTATTAATGTCAAAAATTAAAAAAAATATAAA

CAATATCATTTTAATATATTTTTAATTAAAAAACACTTTTACCACACCGCATTATCAAATATACACTTTCTTCAATTTAAAAAATGAGCACAACACTTTT

AATTAAAGTTATTAAATCGAGCTCGGTCTAAAACTTGGGCTATAAGTTAGCTCAGATTGACATGAATTAATTTAAGATAAAGTAATTTTATTTTTAATTT

TTTATCATTTTAAAAAAAACTTAAAATCAAACCTAATGTTGATTGATCAGTCATCTGATTTTTTTAAAATTCATTTATACTAGATCTCAGGTTGGCCTAT

AATGCAAAAACTGAGTTTTATAACAATACTTTTTATAGAAAATTAACAGGAGCTTGGAAAAGGTCAGGGGTTCGACCCCTCCTTGTGTATGGAGAACATT

TTTTGGGGAGCACTTTACCCCTCTGTGGGCCGACCCGGTGCGAGCATGGATTAGTCTGGACCAGCGTCCAAGACACCGCGTGGTTTATACCAAAAAAAAA

AAAAAACAGGAGCTTGGAAGAGATGGGTTATGAAAGGGATAGTAAAATTATGGGACAGAAAAACATCAACAAAACAATTCACGGGAGCTCCATTTATTAT

AGGAAGCTTTTGAACGTAAAAAAGTTGTGTTATGTTTAGTTGCTGTGAGGAGACATTACTTAATATCACATTCAATTATGCAACTCAGAATTTAATACTC

ACATGATCACAGGCCCTACCTGTCCCATTTCCTTTTCATCTTCCCCTACTCCTCCTCGCATCTTCTTTAAATATAACTCTCACAACTTCCTCTGATCACA

TTAATAGCAAACCAAAAGGGCCATAGACATTCATTGATCGCGAGACACCATTTAAGCCTATACATCATCTCTTTCTAGTGTTTGAGATCAAGAGAGCAAG

>PtOFP23

CTAGAATATTAATTCCATGTATAAATTACACTCTAGTCCTTAATTTCTAAAACTTATTTACAATGAAGTCACACTTGATTAATCATTTCATTTTACTTTC

TGACCAATAGTTCTTATGTGTATGACCCATTAGATTTCCTAATAAGTTGACCCAAATATAAGTCACATCCTAAATAAAATAGGATTAAAATAATTAATTT

ATTATCAATTCAATAATTGATTGATTTATATTTAAAGATCAAGTATAATGTCTAGCAACCTGTCATGATCCTCTAAATATTAGGAAAGTTATAAGTGGTT

TGACTTAAACTTTTAGTGACCAGTTTCTCAGTATAATTATCATCACTTTCTTAGTGTAATTATCATCCCTTCATCAACAATGTTTCAATTATTTTTGTTC

TTAACACCATAGTCATTGATTCTTTGAATAAGATTTGAAACTCTTTTCAAATCTTATTTCCCCTTACACAAGGATTTACATTTAATACTATTTGAGAACA

TATGAAATATTTTTTCTAATTCATCTAGGGTGATGAATCCTCTATTGATTACTTAAATACATTCATATAGTTCATGTTATACCCAATATTTATCTATTTG

CTACCCTTGATTAGGATAACGTGTAGCAGGATCAAAACATAACACACCCTGTATAAAATAACTTAGTGATATCAAGTTTAAGTATCACTTATACAACTAT

CATGTGAGTCTTTACATAGACACAAGTGATCTCTCCATATGAAATTCTCATGTGGGTCAATTTAGTGTACATATCATCCAACAAGCATCTACATATTAGT

TCTAGTTGTTTCTTATACATTAACTTATGACAATAACTACTTCCTTTTATAAATAAAAGAAGATAATATACATTAGTCTTAACAACTCTAACTAATGTTC

AGTTTTAATAGAGCATTGAGCAGAAATATTTAGAAACAATACTTTGACGCAATAGGAATCTCATATTTATAAAAACTTTATAATTTCCTTTACAAATAAT

TTTTGTCCCAAAAATTTTATTTTTACTCTAGATTTATTAATCAATTATTATATTCAATAATAAAATATTGAATAAACATTAAAAATAAATAAATTTTTAT

TAATAAATTAAATATATTTATATGAACAAATTCAATATCACTTATAATTCATCGATTTGCTACCTTATATATACACTAACAATAGCACCTTTTATGGCAA

CAGGAAAAAACAGATATAAAGAAATTAATATCAATAAAAAGAAGGACAAAACAAGTGTTTTGTCCATCGATGCTAGTGGATGCTTTAGTAGTATTGGTGT

TGCTGGTGAAGAAATCGAAACTCTACTTTCTTCAAGAAGTTTCTCGTATGATTCTTCCTGTGAATTCAGCCATTCAATGGATAGAATAGCTGGGAAATCA

GATTACAGAACTTTTAATAGACCTACAGGAAACAAAGTGAGCAAGCTAAATAAAATTAAAAAAAACTAGGACGCCAAGTTTCATTGAACAAGTGGAAAAG

ATCAAAGACTTTGTCGTCCCTGGAGATGATGCAAGCTTCTGTGTTAAAAGAGAAGGAAATTAGGGCTGATGGAATTTGAATGCAGAGGACCGTTGGTTTT

ACTGTGTTTTAACCTCAAAACGCAGCGTATCATTTAATTTCTGTCTTCAGAAAAGTTAAAGGAGAGGAGGCCACATGCGGTATTTGAATGAGAATTGTTG

TTGTTTTTTAAAATATTTTTTATTTAAAAATTTATTAAAATAATATATTTTTTTATTTTTTAAAAATTATTTTTTAATATTAATATATCAAAACAATCTA

AAATTACTAAAACATATTAATTTTAAATTAAAAAAAATTTCTTAACTCTGTTTCTCTAATATAATCAACTGGCATTGGAAACTCTCCATTCAAAACATTG

CAACAGCTTGGAAGAAATCCAAGATGGCACCATTTCAATTACACTTGCTCCAGGAGATTACTTCTCCGGTGAGTTCATCTGTTTTGAAGCGGATGATAGC

>PtOFP24

CGAAACTGAATTAATTTATTAAATAAAATAAAAATATTAAAATTTAAATATCATCAAAACTCTGATATCATATTAAAAAATTAATTATTCAATTTACTAA

GCTGACTGAGATACTATTCTTATTCTATAGTTATAACATGGAGGGGGGGGGGGGGAAAAGGGAATGAAAGATGGCATAAAGGACACTAAACTATCTTTGC

TAAATCCAAAGAAGATAGATCAATTATAATGTTTGTAAGAATAGAAATACGACTGAAGGAGGGCCATGAACACAAGAATTTATAAAGAAAGAAAAGAAGG

GACGGCGGCTTAGGAAATGGGAGACACAGAGACAAAATAGGAGAGCATGCGCGCAAGGCTGATTATTAGAATGAAGATCGATGGTAATCAAATCAGAAAT

CAAAGAAATTAAAATTTGATCAAGAGCTTAATTAGCAATTAATTAGATTAATCAGTGTATATATATATATATATATAAAAGGAGTTCGGAGTTTGGAGTT

TGGAGTTTGGAGTTTGGAGAGGAAGGGAGACGTGCAGCAAGCAAGCATTGGTGCCCATGCGTGATGTGATTGGTCGGTCTTACTTTGATGTTTGCTTTTG

AGCCGCACGTGAAAGACTAATGGCTTGTTTCTGTTCATCTCTCCCTGTCCCTTCACCCTCCTTCTTACCACTAGGGTTTTTTTTTATATAAGAACAATTT

GGACTTTAATTACATCCAACTATTTTTATTTTTTATTTTTACCTGTGCTGTGTAGCGTGCTGAATTTCATTTCTTTATTCTTTAATATTAAAAAAATTAT

AAAAACATTAGGAAGTAAGAAGTTCAATGCATGCTAATATAAATACTTTTAAAATATTTAATCGATAACATGTTGGAAGAAGTATTTTTTTTAAATAAAA

TATTAAAACAAATTAGAAAGAGCAATTATAATAAATCAAATATTAAAGATAAAAAATATTAAGGCGACACTAAGATATTATTCTCAAATAATATGATTCT

TAATTTAATAGTATAAAAAGGACGACACTAATTTAATAAAAAAACAAAGAAAATTATGGTAAATCTCCTAAATTTAACTTAATATTTCTAAATTATAACT

TGTGAAATTTTATATCGGTGCGTAATTGAGAATGTTAAATCCTAACTAATTTAATGCTGAAAGATAAATATATAAAAAAAATTGCAATCCATAAAAACTC

ATATCCGGGTTTAATCAACAAAAAAAAAACCTCTAATAAATCTATATTAGCGGTTGTCACAACAACTAGGATAATATTCCCAAATAATATGAAAGAAGCA

AAAGATAAGTATAGTTAATTTAATAATATCCAAAATTCATTGATTCACGAAAGGGCAAAGTCCAGATAACAAATAACATCCACACTTACAAGGATTTATT

CTTCAGGGATATTGATTTCTTTGTATCCTATCTCTTTGGGAAAAAAAGAGTTTATTAATTAATCTATGCTCAGCATAGTATAATCTAAGATATATCTGCA

ATATTTTTTCCACGGGATTTAGGTTCTGTTAATTGAATAGCGAAGTTAAGAAATTCATGCAGAAATATCTCCTACGTTCTTTTTCATATGAAAGCATTGA

AAAGTATTTTCATTGCTATTTGAGAGTGACAAGACTACGGCACCAAGAACAGCAAAGCCTATGGGATTGAAATTACCTTCCTTCCGAATTAAGTATTGAA

ATTGGTTGTACATGATTTTAGGTTTGTGCTTTCATGTAAAGAAGAAATATGCAGTATGGACCAAGACAATGAATCAACTCGAGAGTACTGCTATAACTGC

CATTCACATGGATTCTCACATGGCAGGCACCCGCCTGGCATATCACAGAATATGCAAGACTGCATTGATCATACGGGCTGCTTCTACATTAATGTTCTTT

GGGTATTAATGGTTTGGAGTACTGGAGCTCCTCCAGCCAACAAATGAACATGACAGCACACATGGAAACCCATTGTTCATCTTTCGTGCACTCATCATAT

>PtOFP25

TATTCGTTTGCCTTTTATTTATTTTTTTCGGATGAATGGGATAATCTTTAATATGCATGTAATATAAATAAATGTGTATGTTGATTATATGATGAGTACA

ACTAGTTAATTTCTTAAATATTTATATATGTTGCATTATTTTCCGAGTATTCATCAATTCTTGAATTTGCACTCGCGTTCTGTTGAACTAAACTCTATTT

GATATGATATACATTGCTTTAATAATCATGCAAATATCTATTATGTTTTGATAGCGGACTTATCAGTTGCTTCATACTAACGAAGAGTAGTTAGCCTATG

TGCATATAGTATTCTGTAAACTTGGCTGGTGAGAGAGGCACCAACCTTTCACGACTGATCCTCCCATAATAGTAACCTTCAGGTGTCATCTGATTTTTAA

CATGTATTCCACTACTTTATTTTAATATGCTTAGTATGTATATTTATATCAAGATATATATACTTGCAAACTATAAATATCTAATATGTATGTTAAATGT

TATTTTCTCACTGAGTTGGTTGAACTCATCCTTTTATATTTTTAATATTATTTCAGGTTCTTAATTTCTGCTAGCAGATGGATTTTGTTGAATGTTTTTA

CTTCTTCAATTTTGATTGGTATACCTTATTTGTTCCACGAGACTTTCTTTTTAGTTTTGATTCGATATTTCAAATGCTCCACTAATACACTATTTTAATT

AAGTTTGAATTTTGACAATATAATAAATATTATGAATTATTTATTTTGTTTTATTCTAATGATGAAATATTTTGAAAGTTATGAAATGCTGTGAATTTTA

TCTTGATATAGTAGAGAGAGTACGTTTAGCCCAGGTTAGAGTCGGCTAAAGTTGAATAATACCTTGAACAAAATTATAAATAAAGTGTTTTAAAATATTT

AAGAAATATTTTTTGAATTATTTTTTATTTATGTCTTAAAAGTGTGGTTTTGAGCAATCTTCATTTTGTATTTAGAGTTTTTAATTGAGTGAGGGGTATT

TTTCATTTCATAAATAGCGACAAATATATCTTAATTATTTTTTAGACTTTTTTTTGTTAAGTGGGGATCTTGGATCTTTGCCCAAGTTGCCCATGTACTC

TGGTCTAGGTGTATAAAATAATAGTGCTTGATGATACCAAAATTTGTCAGATTATAGATTGCTTTAAAAAATAAAGGGTATTAATTTATCAAATAAATCT

AAAAACAAAAATTTCAATTTATATCAACAAGAATTTGTTAAACAATATTGTTTCAACAAATAGAAATGTTTGTTAATTGACAAGATTACACATACAAACT

TGATATAGTGGCCGTGCATATGATAACTTTGTTTACTTAAATAGATATCATTTCCAAAAATTGAATTACTGTTTTCCCCTGACGTCTCTTTCTCCTCCAT

TTGTTTGTGCGTCAAGCTGGCCTGATAGCAGAAACTTCCGGTAAGGATTTTCCCAAAAGTCACGAATGGAAATCACTCAAATCAAAGATTTATTAACAGG

AAGGTATGGCGCCCCAAATACATATCGCAAACACTGAATCAATCCATGACTCAAGCCATTAACGTCGAAATGAAGCAAGAAAGATGATTTTATTACCATC

TTTAGATTCACAATTACTGATTTAAACACATAAATATCAATCACAGTAAAACTCAGCAGCCCATAAAATAAATCAAATATTGGTCAAACCCTAAATAATC

TTGTAACCATATATGCATACTCAAAATCTTGCATGGAAGGTCAATGGTAAAGAACATTAGCATTTAATTACTGTCAGTTTTTTGAACAGTAGCATTTAAT

TACCGTCAGTTTTTCTTCTTGTCAAATTCTTTTACCCTACGTTTATTTTTTCCTGGTGGTTTTTTTTTATTATTATTATTATTATTATTATTAATGTTAC

CTTCCATAATTTTCTCATTTATAAACCACCACCATCTCCATTTTCCCATTAAACCCTAACCCTCGTCACATATTTTTTCTTGCCAAAAAACAAAAAAAAA

>PtOFP26

AAAAAAAATATTAAATCCGGTTAAGTCCATGATTTATATCACAAGTTTGACAAGTTAAGCCACAACACCCAAACTATTATTTTTTCTTCTTTCTTCTTGT

TTAATGCATTTCTTGTCACTCAATTTTTTTTTTAAAAAAGATACAATTACTATTTATTTTTAATATATTTTCTATTTTGTTTTGCAAGATTATCCTTGCG

GGTTTCGTAGGTCAACCTTGGTTGACTTATGTAAATCAAATATATTTTAATCTCAATATTAAATAAAATATCACCTAGTATGCAATTGATTTTGAATTCG

GAGTCTTTTTTTTTTAGAAAACATGTTAGTGATGCATGGACATTTATTTGGCCTTAAATAAAAATATTATGCTCCGACCCACTACATAGTACATGCCGAT

TCTATTATTGTTTGCTTAAATAAAGAATTTATTCTTAAAAACAAAGTTATTAGACAAAATTGGATGCATTGCCCGTGTTGTGAGATCAAATATTTTGATT

TACATTTATATTTATATTTTTTGAGTTTAATCTTTAGTTATTGTTAATAAATTTTTTATGTTTTATTTGCACAGGTAATTGTTTTATTAACAAATAATTT

ATTCAAAGAAACAAAGTTATTAAACATAAATAAATACGCGACCATGTTAGACCAAATATCTTGATATACATTTATTTCTTGATTTTTTTAGTTTAGTCTT

TACTTATCATCAACACTTTTTTTTTACATTTATCAACACAAATAATTTTTTATTTATTTAATTAAAAGCATTCATAAACTTTTTTATTCTTTTATTAAGA

ATTTTTTTCATGGATAAAAATGTGTTGAAAAAGCCTCTCTCTCTCTCTCTCTCAATATATATATATATATATATATATATATATATAATAATAATAATAA

TAATAATAATAATAAAATTACTCACCCTCCGCGCGGGTCAAATATGCAGTTATATCTAATTAATCTCACTTGCAAAATCACCACAAATTTTAACTTCAAC

ATATGTATTTTGTTCATTAAAAACTTGAGCATTTTAATTGGTAGCCATACTTGAGATAGTAGAGGATTCTAAACAGTCAGGTGTAGATCTGCTATATTTA

GATAAGAATTTACCGTAGACATATTATATGGTTGGCCTAGCGAATGCATTTATGAACGACACATTGTTTGCATTTTTTTTTTTAAATAAGTCATCTGATC

CTTTAAGGCCCACGCACAGAGCCATTCCTTTCATAGGCTCGTGTGTGTCTAGGCTCTTTTTTTAGGTTTTGTTTTTTTTTTTTTTTTAAATTTTTTATTT

AGTATTTGGATTAAATATAATTTTGTTTTTTAAAAAAATTTAAACCCGGTTAAGTTCATGATCTAGATCACAAGTTAGACAAGTTAAGCCACAATGCCCA

AACTATTATTTTTTCTTGTTTAATGCATTTCTTGCCCCTTAATTTTTTTCTATTTTTTTTTTGGGTTTTGTAGGTCAATCTTGACTCGAGAGGAGGTCTT

GTCGCTTCAAATCATAAAAATAGCCTAAAAAGGGCATACTTGTTTATCTTACCGTTCGATTAAGCTCAAAATTTTTAAAGTATTTATAGAGGTTATTTTC

TCTTTGATAACCCTTTTAGCACTGAGTGGATTGTCAGATGTTGAGATACCAAAAATTCCACTCCGAGCCCTCGATTTGGGTTGATTTTGTTATTTTCCTT

TATTGTTTTGGATTTGATAATTATTTTCTTTGTACTTTAAGATTTTTATTTTGTTTTATAAGATATGGTTTCTAATTTAATTAAGGTTTTGTAAACCTGT

AATGAGACAAATTTCATGAGTGTTACTTTTTAGAGAAAATAAAGTTATGAATTTGGATTTTATATTTACAAACTTTTTCGCTTATTAAATTTCTAAGTTT

TAAGCTTGTTTATTATCTTTAGCTTTGGATGCATCGATAAGTTTGGGGAAAAGGAGATTACTTTAAGGACTCCAGCAGCCCTGCAATACATATAAATTGT

>PtOFP27

AAAAAAAGTGTTTCTATAAGAATTTTTCGTGTTAGGAGCAGCTGAACAAGTGTCATCAAGGTGTTTATAAATGTTATAATGTCGTGCTTCACAATCATAT

ATATTCAAATGTTGTGGTTCTAAACCACGATATATAATTAAATATTACGGTTTTGAACCACAACATCTTTAAACTATTATATTTTGCTAATATTTTTTCA

TTGTTTGTTATTTCATCATTTATTTGTATTTTGTGCGCCGAAAGGACTTTGATTTTCACGAAATGAAATACCCAATTTTGTCTTCATACCAGCAAAAACA

CGTTTCCAACTAAAAGCCACCACCAATATGTTCAAAGCGAATGTGCAAGTGGAATGAGCAGTTCTATATATGTTCTCACAGTTGTTTATTTGATATAATC

ACATTTCTGATAAAATACATGCTTATGTGCTCTGCTTTGTTTACTGAATTGGCATTGCTGTGAGCATTAGTTTCAGCACATGAATAATTAATGCTTAGTT

CCCGCTGCATCAGATTTGATACTTCAATGAAAAAAGCGTGCACTCAAACCCTAACTTCTTAATTCCCATCTATTTATTTATTTATTTATCTGATCAATGG

AATATTTAAAAGTAAATTGAGAATCACACCAGCAAACAAAACTCTCGGTTAACTAACAAATCCATTGTAACCCCATTTCTCTACCACTCTTCCAGCTATA

AATGCAGTTAGCTCACTTCTTCTTCTTCTTCTTCTTCTTTTGTTTCTTCTGTCATTTTTTTCATGCACTATTTTTTTACCGCTAGGTACTGTGTGCCTAT

GATTTAAGACTAGGACAGGTTTGGAACCCAATTTTGTCTCTACCGCCCTTGCTCTATGTCTCACTTATCAGACTTGACTAACAAGAACTCTCAAAATTAA

CTCCTCTTTGTGGTAAATTCGAGCTGGATACTGCCAGGCCAAGTTTTTAGAAATGAACCTTAAAGGCCGAGCCATTTGCATTGGTCCAGGGCAAAAGCAG

AGCAGCAAACCTTTACTTTAATCTATTGCGAAGTTTGTACGATTCTGAATCTTTCATAATCATAACTTCATATATTTCTTGAAAATGGGGGATTTTCTAC

TTTCTCCAATACGTTAAACCGAATCTCTTAAGTATTCATAGATTGACCAGAGTGTTAGGCTGGAAAGCAAAATTTTCTACAGGTTGGTGGGTATTGCTGG

CATTGTAAGGGTAGAGATGAAAGTATCAGGATTATGTGTGCTTCCATTAAAATAACTTTCACATTTTATTGAGTGAAAATAGTTTTTCACCATTCCCTAC

ACTAACCCAGGGAGTTTACTTAAAAAGAAAATGTTCCATGCTAAAACATATTCGAGTTCTTCATAGATTTCAGTGTATGTTGTTATATTGTTTCATTTGC

CAAAGAAGCATTGTCAACGGCAGAGATTAGCCTAGTTTTGTCCCTTTTCTTCTTTAACCTAGTCCTCTTATCATAAACAGAACTTGATCGTCGTTTGGCA

GGTAAAATTAAATTTTACCATCTTAATTGATGACGTCTGTAAAAGTTGATTATTTTATGAACATGTCAAGTGCCAAAAGTTAGAAGTTTGGCACTGGGAC

TGTTGTATTATCTTTTATAAGAGTGAAATGCCTGCTTAATTAAATACGATGCTCAAGCTTTCTTATGCAGTATTAGTTCCCTTACATTCGAAGTCTGACC

TGTACATGGAAAAAGGCCATTAATCATCTTCAAGCATATTTTCATAATTAATTTATGATTCCAGCTTCGTTACAGTTGTCTTCAAAGCATGGCATAGTAC

CTCCTTGGATTAAACTAGAAAGAAGAAAACAATGGTGACGTTACTAAACTACTTTCTTCATAAAAGATGCAGCTTATCAGATGGCCTTTAGAACCAAGTC

AAACATTTGATTTACACAATCCCAATATGACAATATTTATCTGATCTCACACCAGGAAAAATAAAAATAATTATCTGATAAAGAATAATTAGTGTACGGT

>PtOFP28

CTCTTTCAGGTTCTGTGGTGAAGTAATAAGAGTATCTTTGATGAGAAATGTTGGGTTTTTGCGATGTTACAGTTCCTGGAGGTGGTTTTTTCTTGGAAGC

TCGAGAGGTGTAATGCTTTCTGCTTTTGCTCATGTCCTTGAGCTTGTAAAACCAGGCGTTTGGTATCATATCTGATAATCTAAACCTGTAATTACCCATT

TTTCTTTCCCTCTCTGAGATTTGTCTGAGTTGGTTTACCTTTCCCTCTCTTTGATTACTGAAAAGATAAAAAGAGCTTACAAAGTTTGGGTTATAAAAGG

TAAAGAATAGATGGGGGCTCACTAGAAAAAAGTTCAGCATGAAAGCAGGTGTGTTCCTCTTACATCATAACAACTAACAGAATTACAAATCAGAATTTTT

TTTTTTTGGATTAGACAAACATAATCAGGATTTGTTATGCATAATGATCTCCAAAATTCAACTTTACTAAAAGAAACTGAGGGCCTGAAAACATTGGCTA

AAGTTTAGTCTGTGAATCACTTGTACCATTTTATATACACTCCTGTGGCCAGTAGCCAATATATCGCTGGAACATGAAGATTTGTGAGAACACCTCATCT

GGGGCCTCATCTTTTGCTCAGCCAAATCAAGGGTTAAGAGAGAGTAATGATGATTTTAGCATTAATCAAGGCCCATATGTCTACTAAGTGCTGTGTTCTT

TCCCATGATTCCATGTTTCACAAATGATGATATTCCGTGCCTTTTCAACTTGATTTCACAATGCCCCACATGCCTGGTGCTGCACATTCAGTTTCGGGTC

CCCTACCAAACACTTTATAGGTTTTTTCACTTCTCTCTTCCCAGCTGAGGATGAAATTGCATTTCTCCAAACTGATGGATTCCGATACAGCAACAGGTGA

AGGGAGGTCAATCACATGCTGGTTTTGTCTGAGGCACACCTAATTCTGGCTACTGAGTTTAGGAACTAGGCTGTGCTACTGTGGACCAAATACAGTTAGC

AATAATTTAGGAATCATGTATGTTAAGGATCACAACAATTATTTCATCAACCTTCTGAAAAATTCCTTAAGAAAAAAAAAAACTTCGTCAAGTATGAAAT

TTCTTATTCTGATATATATAAGACATTCTCTCTTTACCGGGGACTGGAAGTTAATGGCATTCAAGCGTTCAACAAATGGTTTTTTGGCAAGAGTTACTGA

TTGCGCTGCCACCTCGACATCCATTGTAAGAGCAGCAACTATAAAGCTAGTGTCCAACAACTTTTCTGGACTCAGAGGCCAAGACTAGATGGAAAAGTTG

GTTTCATGGCTATACTAGAATTAGACTGTGAATGAAACCAGAAGAAGCACAGCCCAGATTGAGGATGGCTTTTGTGAGAAGCAATTGGAGTCTCAATGTG

TATGGTTTTGGGGCTTTAGGAGCTAGATTTTAGCCCAAAGAGAGCACCAGCCACCAAGGACAAGGTTGTCTTGGACTTTAGCTTTACTAACCACAGTAAT

TATTATTTTATCCTTTCCTATACAAAGTCTTTGCCTTGCTAGTGGGTGGGGTAGAATGATATTTTTCACGGATTTCAAAGGTCACTTGCAAATTAATTAG

GGGTAATTTAGTAGTTTATATTTTTTTTCACAGTTAAATTATATAGATACCCTTAAGGCAATAAAATAAAATAAAATGAAAGGTGCGTAATTTTATTTTT

GCACTGTTTACAATTACTTCATTGTCCTTAAAGTAAAAAAAATAAATAAAGGATGAATTAAAAATTATTTTAGTATTTTTCTCTAGGTCTTTTTGTTATA

CTTTATTTCGATTAGGAGTAAATTAGTATTTTTATAAAAATAAAAGAGAAGAAAAAGCTACTAACCCATGCATCACTCGCGCCAATAAGCGAGAGGTTTT

TGCCGGTTATATATTTGGTAAACATGGCTTCCACTACATGGCTTTGCTAGCAATCTGAAGGCCACATATGAAGTTGGTAGATTATTCACAAATTGGAAAA

>PtOFP29

AAATTAGCCGAATTCATTGATAACATCTCTGTGGAGCAATTATTCGATAGTAAATGTCTGTCCACTCACATTCATGCGAGGTTCATGCTCATCCATGGTT

GTAATCAATGATGGATTCTGAATCGGACACAGCCAAGGGAGGTAATCACCGGTCATGTTAAAAGAAAAGAGATTAATTTGAGTTTCTTTTTATTTCACAA

ACACATGCCGAAAAGCAATATTTGGCAAACTAGCGCAAGCCAAAAAAATATAGAGAAATAACACAATTTCTGTGTTGCGTTGTTGAGCAGAGCGTAATCT

CTAAGTTGCGTTGTAATCAATAGCTAGGCTGTGCTACAGAAACCAAAAAAAAAAAATAGTTGGCAATAATTTGTAATCAAGAATTAGATAGGTAATCTAG

AGCTGAATCTTATTTCCATAATTTAGAAATCATGTACGGATCACAACAACTATGCCATCCATCTTCTGAAAAATTGCTGGGACCTTAAGCAAGTTCCTAA

CAACCAATGTTGATGTGAAGAGTGAAATCACAGAATTGAGAAAACAAACTTAGTTAATTATTTATACTAGAGAATTAAATTAGACCACATTTCATAAAGA

GAAAAACTTGACAAAATATGAAGTTTAATATTCTGAGAAAAAAAATTAAAAAAAAAAGAGAGAAAAGAAGCAGTCTATTAACAAGGAATATGGAAGTTCT

TGGCATTCAACTAATTTATTATGTCGTAAAGAGTTGCTAATTGCTCAGGTGATCTAGTTAGTATTTGGTTACAATCTTCGGATAAAAAAAATGAAGATAA

TTATAAAAGAGAAAACCCTAATGTTTGGGAATGATAGAAATTCACATCAAAAATATCCTAGAAATATATTATTCTTATATCTTTGTATTTGTTTTTCTAA

CAAAAAATATCTTCATAATTCATGAATACATTAATTCCTATCATATAATATAATTAATGCATTGATTCATTTACTAAAATATTTTAATAATTAAAAGACA

TTACTGATAAAAAAAAAAGGAACTAGCAATATATTTTTCATAACCAAATCTAAATCTAAACTCATGAACCCATATGCTTGTTATCCAATTCACAAAATTA

TTTTGGATTTGAAATTTTTATTATATAAATAGTTTTTTAAGAACATTTTTTCATTAATTTTTAATTTGTTTTATTTTTTTAAATAATTTTTAATTTGTTT

TATTTTTTAAAAAAATACATGTTAATATATACTCATGTTGAATTAGAAAGTCATGTTGAACTTATTTTAACCTCAAGAAAATAAGTGTATCTCACATTTA

ATTTTAACTTAAAAAATCTATTACATGTTTTACAAAACCAATTCCAACAACCATGGACTTCGAATGAATCTAGAAAAGATACAACCCAAATCGAGGATGG

GTTTTGTGAGTTAAGCAATTCGGGTCTCAATGTGAATGGTTATGGGGCTCTAGGAGCTTGAGTTTAGCCCAAAGAGAGCACCAGTCACTACGGTTGCTTT

AGACTTGAGCTTTATAAACCATGGAGGGTACTGTTTTATAATTGTTGACCAAGATGAGGTTACATATTTGGTAAACATGCCTTCCACTTCTGGCCTTGCT

AGCCATCTGAAAACCACGATGGGATCATATTAGTTGGTAGATTATTCACAAATGGAAGACTAGGACCCACAAAATGGAAGACTAGGACCCCTTTTAATTT

AATGGAACTTTGCCTCCAATGATAAACATCTCCATACGACTCTTCTCCACATCAGGATTCTTGTCAAAATATACTCACTTCATGGTGTGCCACTCTCCTC

CTTCAGGACATGAACCCTACTATAACAAAGAGAAGCACAAAGGGCATAACAAACCTCTTACTGTATCCATTTTCTGCAAACTCTCTCTCTGCAATGATAT

GCCTTTCCTACCTCTTTCCTTAAACTAATTAGAAGCAATAAAAGCCAGCATCCTGCTCCATTTCTTCAACAACAATCTAACTGCAATCATTAAAGATTAA

>PtOFP30

AAGTTTTTTCTTTTCTTTTTAATTATCCCTAATAAAAAATAAACATCAGTTTAAGAAAATAATCTGGACAGACAATTTGAAAGAAAAAGGTTTGTAAGAT

ATTCAAGATTCAGCACAAGCTTTATGCTATTTGAATTTCAAATAGCAAATGAATCCTGCAAAAGTGCTCCCATAGTTTCTTGGTCCCCCTGCCTTTACAT

ATAAATGGTAATGGGTGGGTTGGCATTCCCCGAATAAACCTAACATGGAAAAAGTCTGCAAAATCAGGCCGTTCCTACCCGTCTATCTGGTTTTCCTAAT

CCTAGCTAGAGTAGGATATATCCAATTCGAACCATTTGTTGTGACTATGCGTTTCAACACAAAAAAAAAGGAAATATCCATTCCAAAAAAGCTATATATG

AAAGGGAATATAAATAATTGCTCAACTAGCAAAACGGGTCCGGAAAAAAACTATAAATAATTTTGTAAAAAATATTTAATAAATATTATATTATTATATT

TTATACATGTCTGTCTCGATAGTGGACTATTAAGCAATCTTTATTCTGTGCTTGAAACCTTTTATTAAGTGGAGATAATTTTTATTTCATAAAATATATC

TTATTTATTCTTAATAAGGTGTTTTTTCTTGGTTAAGTGAAGGTTTTTGATCATTGTTCAAGTGGATTTAGCTTAAATCAGTTCTGTTAATTAGTAAATG

TTTCATTAAGGTGGTAAATGTATTTGTTTTTGACATTATCATATATAAAGTTAAAAGAAAAAAGTTATGATTAAAAAGCTTTTTCAAAATATTAATTAAG

AGTTTGAAATTAACATGAACATCTGTATAGTATCGATATGCACCATTCTTATCTTTTCACCTTTTTACTTATTAAATTTTGATTTTCATATGTTTTTCAT

TTAAGTGATGTACAAATAATAATTTAATTTTTAATATTAATAAAATTATTGTCTTTCTTAAAAGATACAGTATTCTCGAATGAAAATTCTTACGTGCATC

AAATAAGGTAGTTTTCATTTTATTATTAAGAATTCAAAAATTAAATAAAAATAAAATGTTAACACAAATAAAAACAACACAAGAAAGACAATAATTGTAA

ATGATACAAAATTCTATTTAACAAAAAATCTCTAATTTAAAAACTTATTACTTAATATTTTCTCTTCAACTAGCCTGCTTTTTAATTAAGAGTTTTTATA

ACTTCTTAAATAAATTAAAAAAATGATATAAATAAATTAAAAAACTCAAAATTAAAAGAAAAAACAATAAAAGTCCCAAACAAACTAGAAAATAAAAAAA

TAATAAAAATAATAATAAATCTAGGTGGAACAACAATACCTTCAGCATAACTTTGTCTCACCATTCTATAGGTATGAGCAATTAATTATAGGTCTTACCA

TTCTATAAATGTTCTTGGAAAGCTATTCTCGTAGCTCAATTATGACAAATTCATAATTAACTAATCAGAACCACTTGCTTTAGAATTTTACCTTATAAAC

TTATCTCTTTGAATAAAATCAGTGAATAAAATCAGATCATATGTTAATGAACTTATTCTTCTCGATGACTTGAAAATTATCCAAATAAAAGAAAGAATTT

AATCTAGACTATATAACACCTAATTAAATTGGTTCGGTAATATCCCTCTAAAAAACACAAAATTCTGCGGGAGGAGGGCTAAAATTCTGCTGGAGGAGAG

TGGTTTTTAAACATGGCAAAGGAAGTTCAATTCAAAATAAAGTACTTATGAATAATTTATTAATCATATATCAATAATTAAATAATAATTGACTCTTGAG

GTGGCCTCCTCAATCCAACTTTTTCCAAGTCCAAACAAAGAAGACGACGAAAAACAGCAAAAAAGTTTTATGATTCATGGGTACGCTTTAACCTTCATTA

TTATTATTATTTTAAAAAAAAAAAAAACACTCCTACTTTCGGTTTCTCATGTATTTATATATCAGCCATCCTTATACCGCCTCTTCTCTTCCTTCAAACC
